# ﻿Surprisingly high genetic divergence of the mitochondrial DNA barcode fragment (COI) within Central European woodlice species (Crustacea, Isopoda, Oniscidea)

**DOI:** 10.3897/zookeys.1082.69851

**Published:** 2022-01-20

**Authors:** Michael J. Raupach, Björn Rulik, Jörg Spelda

**Affiliations:** 1 Sektion Hemiptera, Bavarian State Collection of Zoology (SNSB – ZSM), Münchhausenstraße 21, 81247 München, Germany Sektion Hemiptera, Bavarian State Collection of Zoology München Germany; 2 Department Arthropoda, Zoologisches Forschungsmuseum Alexander Koenig, Adenauerallee 160, 53113 Bonn, Germany Zoologisches Forschungsmuseum Alexander Koenig Bonn Germany; 3 Bavarian State Collection of Zoology (SNSB – ZSM), Münchhausenstraße 21, 81247 München, Germany Bavarian State Collection of Zoology München Germany

**Keywords:** Asellota, cytochrome *c* oxidase subunit I (COI), freshwater, German Barcode of Life (GBoL), mitochondrial DNA, molecular specimen identification, *Platyarthrushoffmannseggii*

## Abstract

DNA barcoding has become the most popular approach for species identification in recent years. As part of the German Barcode of Life project, the first DNA barcode library for terrestrial and freshwater isopods from Germany is presented. The analyzed barcode library included 38 terrestrial (78% of the documented species of Germany) and five freshwater (63%) species. A total of 513 new barcodes was generated and 518 DNA barcodes were analyzed. This analysis revealed surprisingly high intraspecific genetic distances for numerous species, with a maximum of 29.4% for *Platyarthrushoffmannseggii* Brandt, 1833. The number of BINs per species ranged from one (32 species, 68%) to a maximum of six for *Trachelipusrathkii* (Brandt, 1833). In spite of such high intraspecific variability, interspecific distances with values between 12.6% and 29.8% allowed a valid species assignment of all analyzed isopods. The observed high intraspecific distances presumably result from phylogeographic events, *Wolbachia* infections, atypical mitochondrial DNAs, heteroplasmy, or various combinations of these factors. Our study represents the first step in generating an extensive reference library of DNA barcodes for terrestrial and freshwater isopods for future molecular biodiversity assessment studies.

## ﻿Introduction

Isopods are a highly diverse group of invertebrates, with more than 10,300 species described to date (Boyko et al. 2008; Poore 2012). Most of these peracarid crustaceans are free-living, but a number of marine species represent bizarre ectoparasites that infest crustacean and fish species (e.g., Raupach and Thatje 2006; Williams and Boyko 2012; Hadfield et al. 2014; Smit et al. 2014). Isopods range in body length from 0.5 mm (members of the family Microcerberidae) up to 500 mm (species of the famous giant deep-sea isopod genus *Bathynomus* Milne-Edwards, 1879) (McClain et al. 2015). With more than 4,500 known marine species to date, isopods can be found from all shorelines of the world down to the abyssal depths of the oceans where asellote isopods have undergone a massive radiation and represent the dominant taxon (e.g., Wilson and Hessler 1987; Wägele 1989; Raupach et al. 2004, 2009). Approximately 900 isopod species colonized freshwater habitats including lakes, rivers, underground waters, and even thermal springs (e.g., Verovnik et al. 2005; Wilson 2008).

**Figure 1.**
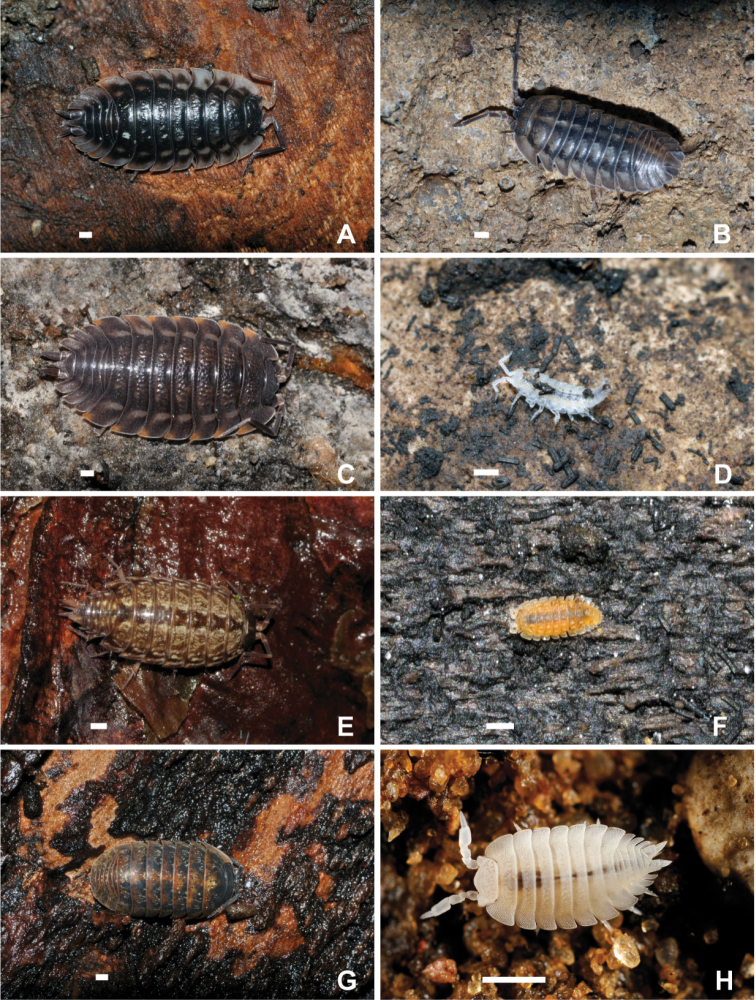
Various woodlouse species of Germany **A***Oniscusasellus* Linnaeus, 1758 **B***Armadillidiumnasatum* Budde-Lund, 1885 **C***Trachelipusratzeburgii* (Brandt, 1833) **D***Mesonicusalpicola* (Heller, 1858) **E***Philosciamuscorum* (Scopoli, 1763) **F***Haplophthalmusmariae* Strouhal, 1953 **G***Armadillidiumopacum* (C. Koch, 1841) **H***Platyarthrushoffmannseggii* Brandt, 1833. Scale bar: 1 mm. Photograph credits: **A–G** Jörg Spelda **H** Armin Rose.

Isopods are, however, not restricted to the aquatic realms only. One group, the Oniscidea or woodlice, are the most successful group of crustaceans that invaded the land by far. Without doubt, these animals represent the most familiar and well-known group of isopods to humans. In contrast to other amphibious crustaceans, e.g., land crabs of the family Geocarcinidae or terrestrial hermit crabs of the genus *Coenobita* Latreille, 1829, no developmental stage (egg, juvenile, etc.) of the Oniscidea requires free water and all biological activities are conducted on land (e.g., Broly et al. 2013). The Oniscidea have evolved a number of unique adaptations, such as the water conducting system, various forms of pleopodal lungs and the cotyledons in the marsupium (e.g., Sfenthourakis and Taiti 2015). Based on the dorsal surface of their exoskeleton, various other morphological traits as well as ecological strategies and behavior, woodlice can be roughly categorized in three main groups (Schmalfuss 1984; Hornung 2011): i) the runners, characterized with an elongate, slightly convex body and long pereopods (e.g., *Philoscia* Latreille, 1804), ii) the clingers, with a flat broad body and short but strong pereopods (e.g., *Platyarthrus* Brandt, 1833), and iii) the rollers, with a highly convex body able to roll up into a ball (pill bugs) (e.g., *Armadillidium* Brandt, 1833) (Fig. 1). Whereas their dispersion ability is rather limited, woodlice are found in almost all biomes of the world except the poles and high mountain ranges (Hornung 2011; Sfenthourakis and Taiti 2015). A hot spot of woodlice diversity is located in the Mediterranean region (Sfendourakis and Taiti 2015), and some species have been introduced to other parts of the world by humans in the past, e.g., to North America (Jass and Klausmeier 2000; Singer et al. 2012; Hornung et al. 2015) and other regions (e.g., Gruner 1966; Slabber and Chown 2002; Karasawa and Nakata 2018). Furthermore, oniscid isopods are amongst the most common and species-rich components of cave-dwelling animal groups with high numbers of troglobitic species (Sfenthourakis and Taiti 2015). In some ecosystems, e.g., European forests, woodlice perform a central role in the decomposition, being largely phytosaprophagous and often occur in very high population densities (e.g., Dias and Hassall 2005; Gongalsky et al. 2005; Hättenschwiler et al. 2005; David 2014; Špaldoňová and Frouz 2014), but also act as important prey for a broad range of predatory arthropods (Raupach 2015). Until now, more than 3,700 species of oniscid isopods have been described worldwide (Schmidt 2008; Sfenthourakis and Taiti 2015). For Germany, 49 species of terrestrial and eight species of freshwater isopods are reported so far (Grünwald 2016).

Since its beginning almost 15 years ago, the concept of DNA barcoding for species identification has revolutionized biodiversity research (Valentini et al. 2009; Cristescu 2014). For many groups of animals, an approximate 650 base pair (bp) frag­ment of the mitochondrial cytochrome *c* oxidase subunit I (COI) gene was selected as marker of choice (Hebert et al. 2003a). The efficiency of DNA barcoding is based on a simple assumption: each species will most likely have similar DNA barcode sequences representing their intraspecific variability whereas the genetic variation between species exceeds the variation within species (Hebert et al. 2003a, 2003b). In this context, the German Barcode of Life initiative (GBoL; www.bolgermany.de) aims at capturing the genetic diversity of animals, fungi, and plants of Germany. Various comprehensive barcode libraries of arthropods, e.g., marine crustaceans (Raupach et al. 2015), spiders (Astrin et al. 2016), and myriapods (Spelda et al. 2011), have been generated in the past. In terms of isopods, most DNA barcoding studies focused on marine species so far (e.g., Khalaji-Pirbalouty and Raupach 2014, 2016; Raupach et al. 2015; ; Brix et al. 2018; Chew et al. 2018; Kakui et al. 2019), whereas for terrestrial and freshwater taxa almost no studies do exist (Asmyhr and Cooper 2012; Zimmermann et al. 2015, 2018a, 2018b). However, no comprehensive DNA barcode reference library has been published for these taxa until now.

In this study we present the first DNA barcode library of terrestrial and freshwater isopods with a focus on Central European species, with a specific emphasis on the Oniscidea. The analyzed barcode library includes 38 terrestrial (78% of the known species of Germany) and five freshwater (63%) species. In summary, 513 new barcodes were generated and a total number of 518 DNA barcodes was analyzed.

## ﻿Materials and methods

### ﻿Sampling of specimens

Samples used for this study were collected between 2000 and 2018 by pitfall traps, sieves, or by hand. Specimens were stored in ethanol (96%) and identified by two of the authors (MJR, JS) using a combination of keys provided in Schmölzer (1964), Gruner (1966), Hopkin (1991), and Berg and Wijnhoven (1997). In total, 513 new DNA barcodes of 46 species were generated. For our analysis we also included five DNA barcodes of the sea slater *Ligiaoceanica* (Linnaeus, 1767) as part of a previous study (Raupach et al. 2010), generating a total data set of 518 DNA barcodes from 46 species. Five of the analyzed species, *Armadillidiumalbum* Dollfus, 1887 (*n* = 1, Spain), *Armadillidiumgranulatum* Brandt, 1833 (*n* = 2, France), *Ligiaitalica* Fabricius, 1798 (*n* = 2, Italy), *Porcellionidessexfasciatus* (Budde-Lund, 1885) (*n* = 4, Mallorca, Spain), and *Tylosponticus* Grebnitzky, 1874 (*n* = 1, Spain) are not recorded from Germany so far but were included for comparison. The number of analyzed specimens per species ranged from one (5 species) to a maximum of 57 for *Porcellioscaber* Latreille, 1804. Most isopods were collected in Germany (*n* = 458, 88.3%), whereas some specimens from other countries were included: Austria (3, 0.6%), Denmark (4, 0.8%), France (3, 0.6%), Italy (3, 0.6%), Luxembourg (38, 7.3%), Spain (6, 1.2%), and Switzerland (3, 0.6%).

### ﻿DNA barcode amplification, sequencing, and data depository

Laboratory operations were carried out either at the Canadian Center for DNA Barcoding (CCDB), University of Guelph, following standardized protocols for COI amplification and sequencing (Ivanova et al. 2006; deWaard et al. 2008), the molecular lab rooms of the German Centre of Marine Biodiversity Research (DZMB), Senckenberg am Meer, in Wilhelmshaven, the working group Systematics and Evolutionary Biology at the Carl von Ossietzky University Oldenburg, or the Zoologisches Forschungsmuseum Alexander Koenig (ZFMK), Bonn, all located in Germany. Photographs were taken for each studied isopod before molecular work was performed. One or two legs of one body side were removed for the subsequent DNA extraction. For some very small isopods with a body length < 3 mm, e.g., specimens of *Haplopthalmus* Schöbl, 1860 or *Jaera* Leach, 1814, partial or complete specimens were used for DNA extraction. In the case of own molecular studies, DNA was extracted using the QIAmp Tissue Kit (Qiagen GmbH, Hilden, Germany) or NucleoSpin Tissue Kit (Macherey-Nagel, Düren, Germany), following the extraction protocol. Detailed information of used primers, PCR amplification and sequencing protocols are given in previous publications (see Raupach et al. 2015; Astrin et al. 2016). All purified PCR products were cycle-sequenced and sequenced in both directions at a contract sequencing facility (GATC, Konstanz, Germany), using the same primers as used in PCR. Double stranded sequences were assembled and checked for putative mitochondrial pseudogenes (numts) by analyzing the presence of stop codons, frameshifts as well as double peaks in chromatograms with the Geneious version 8.1.9 software package (Biomatters, Auckland, New Zealand) (Kearse et al. 2012). For sequence verification, BLAST searches (nBLAST, search set: others, program selection: megablast) were performed to confirm the identity of all new sequences as isopod sequences based on already published sequences (high identity values, very low E-values) (Zhang et al. 2000; Morgulis et al. 2008).

Comprehensive voucher information, taxonomic classifications, photos, DNA barcode sequences, used primer pairs and trace files including their quality are publicly accessible through the public data set “DS-BISCE” (Dataset ID: dx.doi.org/10.5883/DS-BISCE) on the Barcode of Life Data Systems (BOLD; www.boldsystems.org) (Ratnasingham and Hebert 2007). Parallel to this, all new barcode data were deposited in GenBank (accession numbers MN810569–MN810873, MT521085–MT521292).

### ﻿DNA barcode analysis

Following a standardized approach of DNA barcode analysis, the BOLD workbench was used to calculate the nucleotide composition of the sequences and distributions of Kimura-2-parameter distances (K2P; Kimura 1980) within and between species (align sequences: BOLD aligner; ambiguous base/gap handling: pairwise deletion). All barcodes became subject of the Barcode Index Number (BIN) system as it is implemented in BOLD (2020–06–05). In doing so, DNA barcodes are clustered in order to produce operational taxonomic units that closely correspond to species (Ratnasingham and Hebert 2013). Using the given default settings, a recommended threshold of 2.2% was applied for a rough differentiation of intraspecific and interspecific K2P distances (Ratnasingham and Hebert 2013). It should be noted, however, that the BIN assignments on BOLD may change due to the addition of new sequences. Therefore, individual BINs can be split or merged in the light of new data (Ratnasingham and Hebert 2013).

A neighbor-joining cluster analysis (NJ; Saitou and Nei 1987) was performed for all studied species for a graphical representation of the genetic differences between sequences and clusters of sequences using MEGA 10.0.5 (Kumar et al. 2018). Again, the K2P model was chosen as the model for sequence evolution for comparison purposes with previous studies. For validation, non-parametric bootstrap support values were obtained by resampling and analyzing 1,000 replicates (Felsenstein 1985). All analyses were based on an alignment of all studied barcode sequences that was generated using MUSCLE (Edgar 2004) implemented in MEGA 10.0.5. It should be explicitly noted that this analysis is not intended to be phyloge­netic. Instead of this, the shown topology represents a graphical visualization of DNA barcode divergences/distances and putative species cluster.

## ﻿Results

We analyzed 518 DNA barcode sequences of 46 isopod species. A list of species is presented in the supporting information (Suppl. material 1). Fragment lengths of the analyzed DNA barcodes ranged from 407 to 658 bp. As previously shown for arthropods, the DNA barcode region was characterized by a high AT-content: average sequence compositions were A = 24.6%, C = 18.1%, G = 21.5%, and T = 35.8%. Fourteen (30.4%) species had intraspecific distances > 2.2%, with a maximum of 29.4% for *Platyarthrushoffmannseggii* Brandt, 1833. Interspecific distances within the analyzed taxa had values between 12.6% (*Armadillidiumgranulatum* Brandt, 1833; *Armadillidiumversicolor* Stein, 1859) and 29.8% (*Jaerasarsi* Valkanov, 1936; *Armadillidiumnasatum* Budde-Lund, 1885). In total, 76 BINs were found. The number of BINs per species ranged from one (32 species, 68%) to a maximum of six (*Trachelipusrathkii* (Brandt, 1833)). No BIN sharing between species was observed. The NJ analyses revealed non-overlapping clusters with bootstrap support values > 95% for 39 species (95%) with more than one studied specimen (Fig. 2). A more detailed topology of all analyzed specimens is presented in the supporting information (Suppl. material 2).

**Figure 2.**
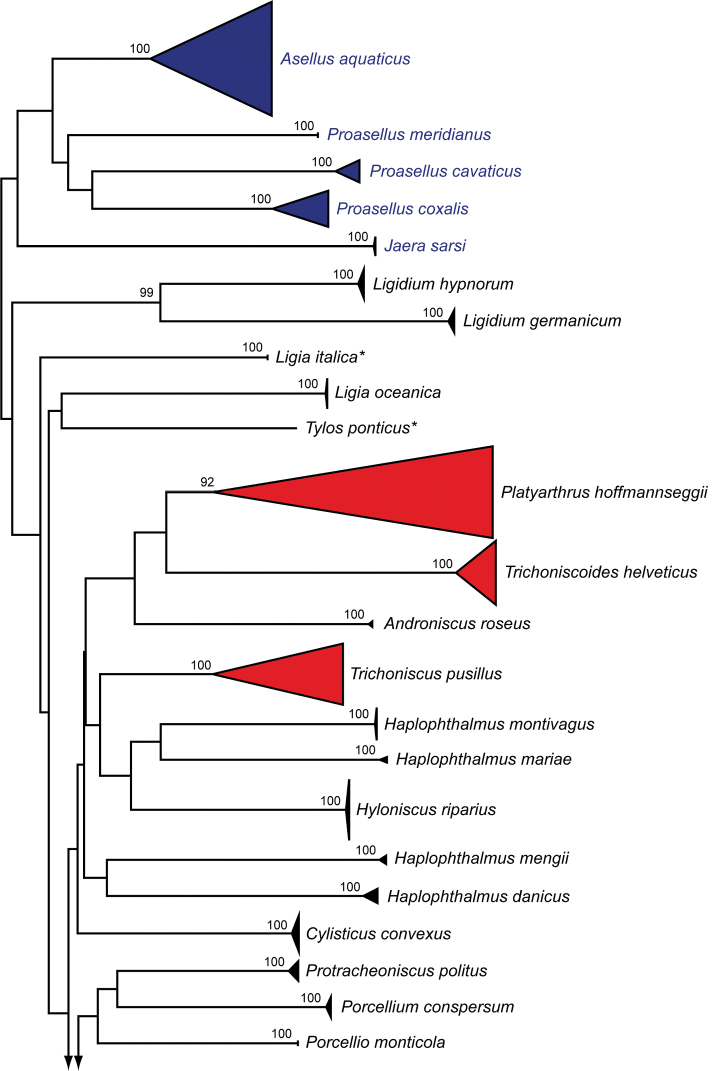
Neighbor-joining (NJ) topology of the analyzed isopod species based on Kimura 2-parameter distances. Triangles show the relative number of individual’s sampled (height) and sequence divergence (width). Red triangles highlight terrestrial species with intraspecific maximum pairwise distances > 2.2%, whereas dark blue triangles indicate freshwater species with such distances. Numbers next to nodes represent non-parametric bootstrap values > 90% (1,000 replicates). Asterisks indicate species not recorded in Germany.

**Figure 2.**
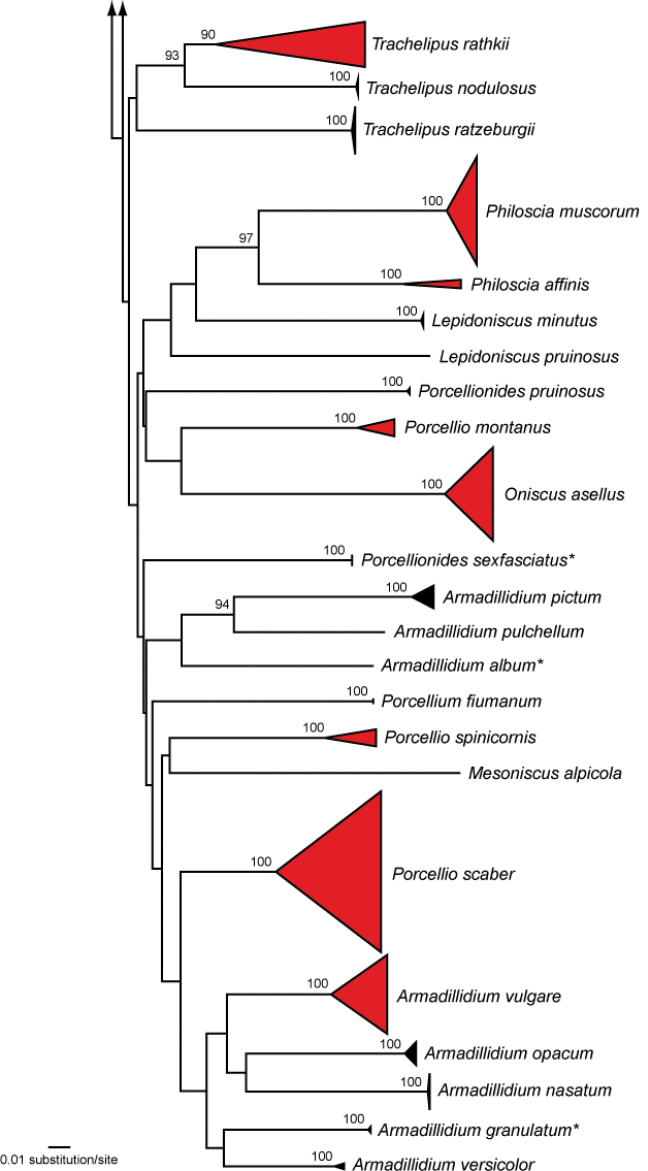
Continued.

## ﻿Discussion

Our study revealed very high intraspecific distances for numerous woodlice species (Tab. 1), including abundant and well-known species as *Porcellioscaber* Latreille, 1804 (maximum intraspecific distances (ISD): 12.16) or *Trachelipusrathkii* (Brandt, 1833) (max ISD: 13.47). Intraspecific distance values higher than 2.2% were also shown for three of the five analyzed freshwater species (Tab. 1). The observed high variability can be caused by a number of different factors and will be discussed in the following.

**Table 1.** Molecular distances based on the Kimura 2-parameter model of the analyzed specimens of the analyzed isopod species with intraspecific distances > 2.2% using the BOLD work bench. ISD = intraspecific distance. BINs are based on the barcode analysis from 05–06–2020. See methods for explanation of basis.

Species	*n*	BINs	Mean ISD	Max ISD
*Armadillidiumvulgare* (Latreille, 1804)	28	AAE6611, AAH4108, AAH4111, AAU1529	3.76	6.44
*Asellusaquaticus* (Linnaeus, 1758)	41	ACF1266, AEC4774, AAA1970	4.25	13.37
*Oniscusasellus* (Linnaeus, 1758)	33	ADM8743, ADM8116, ADK9123	2.12	5.63
*Philosciaaffinis* Verhoeff, 1908	3	ADM8125, AAY1058	3.63	5.44
*Philosciamuscorum* (Scopoli, 1763)	38	AAH4103, AAH4104	0.3	2.98
*Platyarthrushoffmannseggii* Brandt, 1833	33	AAV8050, AAV8051, ADK9658	9.4	29.35
*Porcelliomontanus* Budde-Lund, 1885	6	ADR0694, ADM7742	1.26	3.81
*Porcellioscaber* Latreille, 1804	57	AAC3755, AAZ0248, ABA5892, ADK8850, ADM8147	2.58	12.16
*Porcelliospinicornis* Say, 1818	6	ADF7011, ADI3596	3.01	5.13
*Proaselluscavaticus* (Leydig, 1871)	8	ADX3790, ADW6988, ADX4659	1.61	2.95
*Proaselluscoxalis* (Dollfus, 1892)	13	ACI1746, ACH7545	2.81	5.78
*Trachelipusrathkii* (Brandt, 1833)	16	AAH4102, ADK8699, ADK8533, ADM8087, ADM8088, ADF6188	6.89	16.59
*Trichoniscoideshelveticus* (Carl, 1908)	23	ADM7247, ADM7248, ADM7249	1.07	5.46
*Trichoniscuspusillus* Brandt, 1833	22	AAN7523, AAZ1993	6.8	13.47

**Figure 3.**
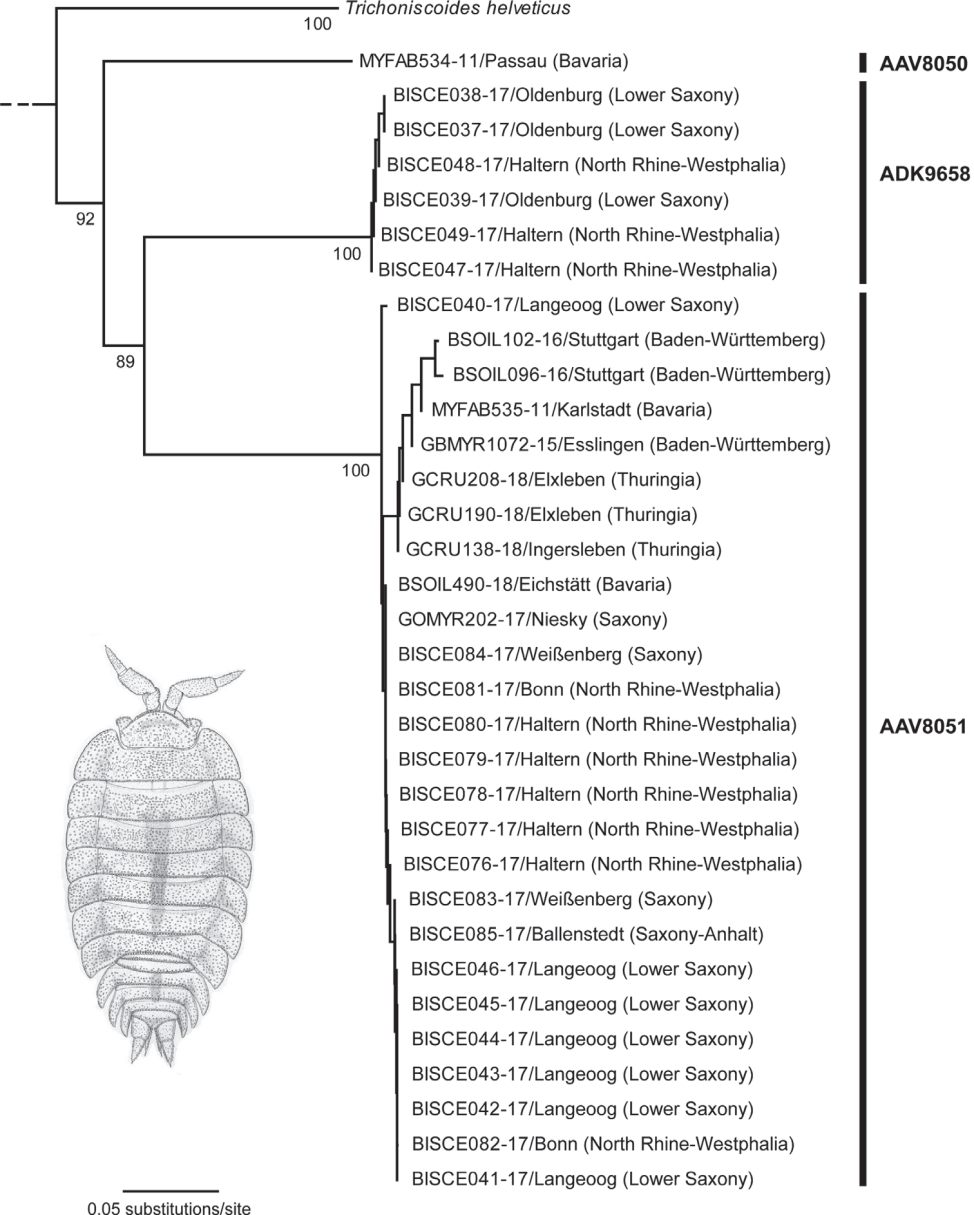
Subtree of the Neighbor-joining topology based on Kimura 2-parameter distances of all analyzed specimens of *Platyarthrushoffmannseggii* Brandt, 1833 and nearest neighbor. Branches with specimen ID-number from BOLD and sample localities. Numbers next to internal nodes are non-parametric bootstrap values (in %) with values higher than 80. BIN values are based on the barcode analysis from 05-06-2020. The isopod drawing by Christian Schmidt was obtained from Raupach (2005).

First, phylogeographic events may generate different haplotypes and distinct mitochondrial lineages. In the case of European woodlice species, numerous studies showed complex phylogeographic patterns correlated with high variability of the studied mitochondrial markers including COI, e.g., for species of the genus *Alpioniscus* Racovitza, 1908 (Bedek et al. 2019), the common sea slater *Ligiaoceanica* (Linneaus, 1767) (Raupach et al. 2014), *Ligidium* spp. (Klossa-Kilia et al. 2005), the common woodlouse *Oniscusasellus* Linnaeus, 1758 (Bilton et al. 1999), *Orthometopon* spp. (Poulakakis and Sfenthourakis 2008), *Helleriabrevicornis* Ebner, 1868 (Gentile et al. 2010), or two species of the genus *Trachelipus* Budde-Lund, 1908 (Parmakelis et al. 2008). Similar results have been also demonstrated for freshwater isopods of the genus *Asellus* Geoffroy, 1762 (Verovnik et al. 2004; Verovnik et al. 2005; Sworobowicz et al. 2015; Pérez-Moreno et al. 2017) and *Proasellus* Dudich, 1925 (Ketmaier 2002; Eme et al. 2013; Kilikowska et al. 2013). Our data set revealed extremely high intraspecific distance values for the myrmecophilous isopod *Platyarthrushoffmannseggii* Brandt, 1833 (*n* = 33), with a maximum value of 29.4% (Tab. 1). It is a small, white, and blind oniscid isopod that is widely-distributed in Europe and strictly associated with various ant species (e.g., Mathes and Strouhal 1954; Gruner 1966; Parmentier et al. 2017). A few other species are found in the Mediterranean region, e.g., *Platyarthrusschoebli* Budde-Lund, 1879 (Garci and Cruz 1986), which can be easily differentiated from *Platyarthrushoffmannseggii*. The NJ topology revealed three distinct lineages associated with three BINs (Fig. 3), but no clear correlation of the analyzed specimens to specific sampling regions. Furthermore, we found no ant-host-specific correlation of the observed lineages. For some other species distinct lineages were also detected, but no conspicuous substructures were revealed (see Suppl. material 3).

Second, the presence of the inherited alpha-proteobacteria *Wolbachia* Hertig, 1936 can affect the mitochondrial variability in arthropods (e.g., Hurst and Jiggins 2005; Werren et al. 2008; Correa and Ballard 2016). These endosymbionts are transmitted vertically through host eggs and alter the biology of their host in various ways, including the induction of reproductive manipulations, such as feminization, parthenogenesis, male killing and sperm-egg incompatibility (Werren et al. 2008). If a population is infected by *Wolbachia*, patterns of mitochondrial polymorphisms will be altered by natural selection that acts on these symbionts, either increasing or decreasing the frequency distribution of haplotypes within a population (Hurst and Jiggins 2005). Previous studies documented high infection rates of *Wolbachia* within many terrestrial as well as freshwater isopod species (e.g., Bouchon et al. 1998; Rigaud et al. 1999; Cordaux et al. 2012), including numerous species that have been analyzed in this study, e.g., *Platyarthrushoffmannseggii*, *Porcellioscaber*, and *Trachelipusrathkii*. However, it is very difficult to distinguish demographic variation from symbiont-induced effects of mitochondrial variability (see Hurst and Jiggins 2005).

Third, the amplification and sequencing of nuclear mitochondrial pseudogenes (numts) can obscure the true mitochondrial variability within a species (Bensasson et al. 2001). Numts are nonfunctional copies of mitochondrial DNA in the nuclear genome. As consequence of reduced selection pressure, nucleotide substitutions and insertions as well as deletions may introduce stop codons and shifts in the reading frame of these inactive copies (Buhay 2009; Schizas 2012). Various studies documented such numts for a number of different crustacean taxa (e.g., Buhay 2009; Baeza and Fuentes 2013; Kim et al. 2013). For isopods, however, numts have not been reported so far, and a careful inspection of our COI sequences revealed no double peaks and translation without stop codons.

Fourth, many oniscid species, e.g., *Armadillidiumvulgare* (Latreille, 1804), *Cylisticusconvexus* (De Geer, 1778), or *Philosciamuscorum* (Scopoli, 1763), are characterized by atypical mitochondrial DNA structures that are composed of linear monomers and circular dimers, generating different mitochondrial lineages (Doublet et al. 2012, 2013). There is also a possible link between such atypical mitochondrial DNAs and heteroplasmy (i.e., the mixture of mtDNA genotypes within an organism) which has been documented for various woodlice in the past (Doublet et al. 2008, 2012). However, only a few studies are available until now, and most details still remain unclear.

Finally, distinct mitochondrial lineages that correlate with high genetic distances can give evidence for the existence of currently overseen cryptic species. Considering the previous discussed aspects, however, additional morphological and/or nuclear DNA sequence data are essential for a verification of truly distinct lineages. For freshwater and terrestrial isopods, a few studies demonstrated such integrative taxonomic approaches (McGaughran et al. 2005; Santamaria et al. 2017; Santamaria 2019). In terms of the analyzed taxa, no previous studies discussed the existence of cryptic species, and all specimens were checked and determined carefully before molecular works started.

Based on the given data we are currently unable to clarify the reasons of the observed high intraspecific variability within some of the analyzed species in detail. We suggest, however, that the detected high distances result from i) phylogeographic effects, ii) *Wolbachia* infections, iii) atypical mitochondrial DNAs and/or heteroplasmy, or, most likely, iv) various combinations of these phenomena in many cases. More specimens from different geographic regions as well as additional nuclear markers should be analyzed to verify this in detail. Despite these high intraspecific distances and multiple BINs for some species, however, high interspecific distances in combination with monophyletic lineages allow a correct determination of all studied taxa.

## ﻿Conclusions

The development of new sequencing technologies changed biological science significantly. As a consequence, DNA-based approaches have become more and more popu­lar for the assessment of biodiversity and identification of specimens. Parallel analysis of thousands of specimens, bulk samples (metabarcoding) or environmental DNA (eDNA) will become routinely used techniques in modern species diversity assessment studies (e.g., Shokralla et al. 2012; Moriniere et al. 2016; Brauckmann et al. 2019; Hardulak et al. 2020). Whereas hypervariable regions of nuclear rRNA genes or other mitochondrial gene fragments may represent useful markers for such studies (e.g., Mohrbeck et al. 2015; Gillet et al. 2018; Lopez-Escardo et al. 2018), COI has become the most popular and efficient marker of choice (e.g., Andujar et al. 2018; Curry et al. 2018; Brauckmann et al. 2019; Hausmann et al. 2020). All these approaches, however, rely highly on comprehensive on-line reference libraries of correctly identified specimens (e.g., Brandon-Mong et al. 2015; Creer et al. 2016; Staats et al. 2016). Ideally, such libraries include sequence data of a species` complete distribution range that can provide additional information of phylogeographic substructures that are well-known for many species (e.g., Gentile et al. 2010; Raupach et al. 2014; Paill et al. 2021).

The necessity of DNA barcode reference libraries is also important for the modern molecular-based analysis of soil biodiversity (Taberlet et al. 2012; Orgiazzi et al. 2014; Rota et al. 2020). Reference libraries have been already published for a variety of typical soil-inhabiting taxa, e.g., earthworms (Porco et al. 2013; Pansu et al. 2015; Sun et al. 2018), mites (Young et al. 2012; Young et al. 2019), springtails (Hogg and Hebert 2004; Porco et al. 2013), spiders (Astrin et al. 2016), myriapods (Spelda et al. 2011) and ground beetles (Raupach et al. 2016; Raupach et al. 2018). In our present study we lay the foundations for a comprehensive DNA barcode data set for terrestrial and freshwater isopods of Central Europe.

## Supplementary materials

Supplementary material 1 Barcode analysis using the BOLD workbench Data type Data table Explanation note Molecular distances based on the Kimura 2-parameter model of the analyzed specimens of the analyzed isopod species. Divergence values were calculated for all studied sequences, using the Nearest Neighbor Summary implemented in the Barcode Gap Analysis tool provided by the Barcode of Life Data System (BOLD). Align sequencing option: BOLD aligner (amino acid based HMM), ambiguous base/gap handling: pairwise deletion. ISD = intraspecific distance. BINs are based on the barcode analysis from 05–06–2020. Asterisks indicate species not recorded from Germany. Species pairs with intraspecific distances > 2.2% are marked in bold. https://binary.pensoft.net/file/636211This dataset is made available under the Open Database License (http://opendatacommons.org/licenses/odbl/1.0/). The Open Database License (ODbL) is a license agreement intended to allow users to freely share, modify, and use this Dataset while maintaining this same freedom for others, provided that the original source and author(s) are credited.Michael J. Raupach, Björn Rulik, Jörg Spelda

Supplementary material 2 Neighbor-joining topology Data type Neighbor-joining topology Explanation note Neighbor-joining phylogram of all analyzed isopod specimen based on Kimura 2-parameter distances. Individuals are classified using ID numbers from BOLD and species name. Numbers next to nodes represent non-parametric bootstrap values (1,000 replicates, in %). https://binary.pensoft.net/file/636212This dataset is made available under the Open Database License (http://opendatacommons.org/licenses/odbl/1.0/). The Open Database License (ODbL) is a license agreement intended to allow users to freely share, modify, and use this Dataset while maintaining this same freedom for others, provided that the original source and author(s) are credited.Michael J. Raupach, Björn Rulik, Jörg Spelda

Supplementary material 3 Neighbor-joining topology of the BOLD workbench including BIN analysis Data type Neighbor-joining topology Explanation note Neighbor-joining phylogram of all analyzed isopod specimen based on Kimura 2-parameter distances using the BOLD workbench from 07–06–2020. Individuals are classified using ID numbers from BOLD and species name. Furthermore, geographic information and BIN numbers are provided for each specimen. https://binary.pensoft.net/file/636213This dataset is made available under the Open Database License (http://opendatacommons.org/licenses/odbl/1.0/). The Open Database License (ODbL) is a license agreement intended to allow users to freely share, modify, and use this Dataset while maintaining this same freedom for others, provided that the original source and author(s) are credited.Michael J. Raupach, Björn Rulik, Jörg Spelda

## References

[B1] Andujar C Arribas P Yu DW Vogler AP Emerson BC (2018) Why the COI barcode should be the community DNA metabarcode for the Metazoa. Molecular Ecology 27: 3968–3975. 10.1111/mec.14844 30129071

[B2] Asmyhr MG Cooper SJB (2012) Difficulties barcoding in the dark: the case of crustacean stygofauna from eastern Australia. Invertebrate Systematics 26: 583–591. 10.1071/IS12032

[B3] Astrin JJ Höfer H Spelda J Holstein J Bayer S Hendrich L Huber BA Kielhorn K-H Krammer H-J Lemke M Monje JC Morinière J Rulik B Petersen M Janssen H Muster C (2016) Towards a DNA barcode reference database for spiders and harvestmen of Germany. PLoS ONE 11: e0162624. 10.1371/journal.pone.0162624 PMC5040438 27681175

[B4] Baeza JA Fuentes MS (2013) Exploring phylogenetic informativeness and nuclear copies of mitochondrial DNA (numts) in three commonly used mitochondrial genes: mitochondrial phylogeny of peppermint, cleaner, and semi-terrestrial shrimps (Caridea: *Lysmata*, *Exhippolysmata*, and *Merguia*). Zoological Journal of the Linnean Society 168: 699–722. 10.1111/zoj.12044

[B5] Bedek J Taiti S Bilhandžija H Ristori E Baratti M (2019) Molecular and taxonomic analyses in troglobiotic Alpioniscus (Illyrionethes) species from the Dinaric Karst (Isopoda: Trichoniscidae). Zoological Journal of the Linnean Society 187: 539–584. 10.1093/zoolinnean/zlz056

[B6] Bensasson D Zhang D-X Hartl DL Hewitt GM (2001) Mitochondrial pseudogenes: evolution`s misplaced witnesses. Trends in Ecology and Evolution 16: 314–321. 10.1016/S0169-5347(01)02151-6 11369110

[B7] Berg MP Wijnhoven H (1997) Landpissebedden. Een tabel voor de landpissebedden (Crustacea; Oniscidea) van Nederland en België. Wetenschappelijke Mededelingen van de KNNV 221: 1–80. [In Dutch]

[B8] Bilton DT Goode D Mallet J (1999) Genetic differentiation and natural hybridization between two morphological forms of the common woodlouse, *Oniscusasellus* Linnaeus 1758. Heredity 82: 462–469. 10.1038/sj.hdy.6885170 10383665

[B9] Bouchon D Rigaud T Juchault P (1998) Evidence for widespread *Wolbachia* infection in isopod crustaceans: molecular identification and host feminization. Proceedings of the Royal Society Series B: Biological Sciences 265: 1081–1090. 10.1098/rspb.1998.0402 PMC1689171 9684374

[B10] Boyko CB Bruce NL Hadfield KA Merrin KL Ota Y Poore GCB Taiti S Schotte M Wilson GDF (2008 onwards) World marine, freshwater and terrestrial isopod crustaceans database. http://www.marinespecies.org/isopoda [Accessed 2020–06–05]

[B11] Brandon-Mong GJ Gan HM Sing KW Lee PS Lim PE Wilson JJ (2015) DNA metabarcod­ing of insects and allies: an evaluation of primers and pipelines. Bulletin of Entomological Research 105: 717–727. 10.1017/S0007485315000681 26344799

[B12] Brauckmann TWA Ivanova NV Prosser SWJ Elbrecht V Steinke D Ratnasingham S deWaard JR Sones JE Zakharov EV Hebert PDN (2019) Metabarcoding a diverse arthropod mock community. Molecular Ecology Resources 19: 711–727. 10.1111/1755-0998.13008 30779309 PMC6850013

[B13] Brix S Bober S Tschesche C Kihara T-C Driskell A Jennings RM (2018) Molecular species delimitation and its implications for species descriptions using desmosomatid and nannoniscid isopods from the VEMA fracture zone as example taxa. Deep Sea Research Part II: Topical Studies in Oceanography 148: 180–207. 10.1016/j.dsr2.2018.02.004

[B14] Broly P Deville P Maillet S (2013) The origin of terrestrial isopods (Crustacea: Isopoda: Oniscidea). Evolution and Ecology 27: 461–476. 10.1007/s10682-012-9625-8

[B15] Buhay JE (2009) “COI-like” Sequences are becoming problematic in molecular systematic and DNA Barcoding studies. Journal of Crustacean Biology 29: 96–110. 10.1651/08-3020.1

[B16] Chew M Rahim AbA binti Mohd Yusof NY (2018) A new species of *Eisothistos* (Isopoda, Cymothoida) and first molecular data on six species of Anthuroidea from the Penninsular Malaysia. Zoosystematics and Evolution 94: 73–81. 10.3897/zse.94.23000

[B17] Cordaux R Pichon S Hatira HBA Doublet V Grève P Marcadé I Braquart-Varnier C Souty-Grosset C Charfi-Cheikhrouha F Bouchon D (2012) Widespread *Wolbachia* infection in terrestrial isopods and other crustaceans. In: Štrus J Taiti S Sfenthourakis S (Eds) Advances in Terrestrial Isopod Biology.ZooKeys 176: 123–131. 10.3897/zookeys.176.2284 PMC3335409 22536103

[B18] Correa CC Ballard JWO (2016) *Wolbachia* associations with insects: winning or losing against a master manipulator. Frontiers in Ecology and Evolution 3: e153. 10.3389/fevo.2015.00153

[B19] Creer S Deiner K Frey S Porazinska D Taberlet P Thomas WK Potter C Bik HM (2016) The ecologist`s filed guide to sequence-based identification of biodiversity. Methods in Ecology and Evolution 7: 1008–1018. 10.1111/2041-210X.12574

[B20] Cristescu ME (2014) From barcoding single individuals to metabarcoding biological communities: towards an integrative approach to the study of global biodiversity. Trends in Ecology and Evolution 29: 566–571. 10.1016/j.tree.2014.08.001 25175416

[B21] Curry CJ Gibson JF Shokralla S Hajibabaei M Baird DJ (2018) Identifying North American freshwater invertebrates using DNA barcodes: Are existing COI sequence libraries fit for purpose? Freshwater Science 37: 178–189. 10.1086/696613

[B22] David JF (2014) The role of litter-feeding macroarthropods in decomposition processes: a reappraisal of common views. Soil Biology and Biochemistry 76: 109–118. 10.1016/j.soilbio.2014.05.009

[B23] deWaard JR Ivanova NV Hajibabaei M Hebert PDN (2008) Assembling DNA barcodes: analytical protocols. In: Martin C (Ed.) Methods in Molecular Biology: Environmental Genetics.Humana Press, Totowa, 275–293. 10.1007/978-1-59745-548-0_15 18642605

[B24] Dias N Hassall M (2005) Food, feeding and growth rates of peracarid macro-decomposers in a Ria Formosa salt marsh, southern Portugal. Journal of Experimental Marine Biology and Ecology 325: 84–94. 10.1016/j.jembe.2005.04.017

[B25] Doublet V Souty-Grosset C Bouchon D Cordaux R Marcadé I (2008) A thirty million year-old inherited heteroplasmy. PLoS ONE 3: e2938. 10.1371/journal.pone.0002938 PMC2491557 18698356

[B26] Doublet V Raimond R Grandjean F Lafitte A Souty-Grosset C Marcadé I (2012) Widespread atypical mitochondrial DNA structure in isopods (Crustacea, Peracarida) related to a constitutive heteroplasmy in terrestrial species. Genetics 55: 234–244. 10.1139/G2012-008 22376074

[B27] Doublet V Helleu Q Raimond R Souty-Grosset C Marcadé I (2013) Inverted repeats and genome architecture conversions of terrestrial isopods mitochondrial DNA. Journal of Molecular Evolution 77: 107–118. 10.1007/s00239-013-9587-7 24068302

[B28] Edgar RC (2004) MUSCLE: a multiple sequence alignment method with reduced time and space complexity. BMC Bioinformatics 5: e113. 10.1186/1471-2105-5-113 PMC517706 15318951

[B29] Eme D Malard F Konecny-Dupré L Lefébure T Douady CJ (2013) Bayesian phylogeographic inferences reveal contrasting colonization dynamics among European groundwater isopods. Molecular Ecology 22: 5685–5699. 10.1111/mec.12520 24102689

[B30] Felsenstein J (1985) Confidence limits on phylogenies: an approach using the bootstrap. Evolution 39: 783–791. 10.2307/2408678 28561359

[B31] Garcia L Cruz A (1986) Els isopodes terrestres (Crustacea: Isopoda: Oniscidea) de les illes Balears: catalog d`especies. Boletin de la Sociedad de Historia Natural de Baleares 39: 77–99.

[B32] Gentile G Campanaro A Carosi M Sbordoni V Argano R (2010) Phylogeography of *Helleriabrevicornis* Ebner 1868 (Crustacea, Oniscidea): Old and recent differentiations of an ancient lineage. Molecular Phylogenetics and Evolution 54: 640–646. 10.1016/j.ympev.2009.10.005 19853050

[B33] Gillet B Cottet M Destanque T Kue K Descioux S Chanudet V Hughes S (2018) Direct fishing and eDNA metabarcoding for biomonitoring during a 3-year survey significantly improves number of fish detected around a South East Asian reservoir. PLoS ONE 13: e0208592. 10.1371/journal.pone.0208592. PMC6292600 30543655

[B34] Gongalsky KB Savin FA Pokarzhevskii AD Filimonova ZV (2005) Spatial distribution of isopods in an oak-beech forest. European Journal of Soil Biology 41: 117–122. 10.1016/j.ejsobi.2005.09.012

[B35] Gruner HE (1966) 53. Teil: Krebstiere oder Crustacea V. Isopoda 2. Lieferung. In: Die Tierwelt Deutschlands und der angrenzenden Meeresteile, begründet von Professor Dr. Friedrich Dahl. VEB Gustav Fischer Verlag Jena, 1–380. [In German]

[B36] Grünwald M (2016) Rote Liste und Gesamtartenliste der Landasseln und Wasserasseln (Isopoda: Oniscidea et Asellota) Deutschlands, 1. Fassung, Stand November 2011. In: Bundesamt für Naturschutz (BfN) (Hrsg.): Rote Liste gefährdeter Tiere, Pflanzen und Pilze Deutschlands; Band 4: Wirbellose Tiere (Teil 2). Naturschutz und Biologische Vielfalt 70(4): 349–363. [In German]

[B37] Hadfield KA Sikkel PC Smit NJ (2014) New records of fish parasitic isopods of the gill-attaching genus *Mothocya* Costa, in Hope 1851 from the Virgin Islands, Carribean, with description of a new species. ZooKeys 439: 109–125. 10.3897/zookeys.439.8093 PMC4195937 25317058

[B38] Hardulak LA Moriniere J Hausmann A Hendrich L Schmidt S Doczkal D Müller J Hebert PDN Haszprunar G (2020) DNA metabarcoding for biodiversity monitoring in a national park: Screening for invasive and pest species. Molecular Ecology Resources 20: 1542–1557. 10.1111/1755-0998.13212 32559020

[B39] Hättenschwiler S Tiunov AV Scheu S (2005) Biodiversity and litter decomposition in terrestrial ecosystems. Annual Review of Ecology, Evolution, and Systematics 36: 191–218. 10.1146/annurev.ecolsys.36.112904.151932

[B40] Hausmann A Segerer A Greifenstein T Knubben J Moriniere J Bozicevic V Doczkal D Günther A Ulrich W Habel JC (2020) Towards a standardized quantitative and qualitative insect monitoring scheme. Ecology and Evolution 10: 4009–4020. 10.1002/ece3.6166 32489627 PMC7244892

[B41] Hebert PDN Cywinska A Ball SL deWaard JR (2003a) Biological identifications through DNA barcodes. Proceedings of the Royal Society of London Series B: Biological Sciences 270: 313–321. 10.1098/rspb.2002.2218 12614582 PMC1691236

[B42] Hebert PDN Ratnasingham S deWaard JR (2003b) Barcoding animal life: cytochrome *c* oxidase subunit 1 divergences among closely related species. Proceedings of the Royal Society of London Series B: Biological Sciences 270: S96–S99. 10.1098/rsbl.2003.0025 PMC1698023 12952648

[B43] Hogg ID Hebert PDN (2004) Biological identification of springtails (Hexapoda: Collembola) from the Canadian Arctic, using mitochondrial DNA barcodes. Canadian Journal of Zoology 82: 749–754. 10.1139/z04-041

[B44] Hopkin S (1991) A key to the woodlice of Britain and Ireland. Field Studies 7: 599–650.

[B45] Hornung E (2011) Evolutionary adaptation of oniscidean isopods to terrestrial life: structure, physiology and behavior. Terrestrial Arthropod Reviews 4: 95–130. 10.1163/187498311X576262

[B46] Hornung E Szlavecz K Dombos M (2015) Demography of some non-native isopods (Crustacea, Isopoda, Oniscidea) in a Mid-Atlantic forest, USA. In: Taiti S Hornung E Štrus J Bouchon D (Eds) Trends in Terrestrial Isopod Biology.ZooKeys 515: 127–143. 10.3897/zookeys.515.9403 PMC4525040 26261445

[B47] Hurst GDD Jiggins FM (2005) Problems with mitochondrial DNA as a marker in population, phylogeographic and phylogenetic studies: the effects of inherited symbionts. Proceedings of the Royal Society Series B: Biological Sciences 272: 1525–1534. 10.1098/rspb.2005.3056 PMC1559843 16048766

[B48] Ivanova NV deWaard JR Hebert PDN (2006) An inexpensive, automation-friendly protocol for recovering high-quality DNA. Molecular Ecology Notes 6: 998–1002. 10.1111/j.1471-8286.2006.01428.x

[B49] Jass J Klausmeier B (2000) Endemics and immigrants: North American terrestrial isopods (Isopoda, Oniscidea) North of Mexico. Crustaceana 73: 771–799. 10.1163/156854000504804

[B50] Kakui K Shimomura M Kimura S Kimura T (2019) Topotype-based DNA barcode of the parasitic *Pseudionenephropsi* (Bopyridae), with a supplementary morphological description. Species Diversity 24: 103–108. 10.12782/specdiv.24.103

[B51] Kamilari M Klossa-Kilia E Kilias G Sfenthourakis S (2014) Old Aegean palaeoevents driving the diversification of an endemic isopod species (Oniscidea, Trachelipodidae). Zoologica Scripta 43: 379–392. 10.1111/zsc.12060

[B52] Karasawa S Nakata K (2018) Invasion stages and potential distributions of seven exotic terrestrial isopods in Japan. BioRisk 13: 53–76. 10.3897/biorisk.13.23514

[B53] Kearse M Moir R Wilson A Sone-Havas S Cheung M Sturrock S Buxton S Cooper A Markowitz S Duran C Thierer T Ashton B Meintjes P Drummond A (2012) Geneious Basic: an integrated and extendable desktop software platform for the organization and analysis of sequence data. Bioinformatics 15: 1647–1649. 10.1093/bioinformatics/bts199 PMC3371832 22543367

[B54] Ketmaier V (2002) Isolation by distance, gene flow and phylogeography in the *Proaselluscoxalis*-group (Crustacea, Isopoda) in Central Italy: allozyme data. Aquatic Sciences 64: 66–75. 10.1007/s00027-002-8055-z

[B55] Khalaji-Pirbalouty V Raupach MJ (2014) A new species of *Cymodoce* Leach, 1814 (Crustacea: Isopoda: Sphaeromatidae) from the Persian Gulf based on morphological and molecular characteristics, with a redescription of *Cymodocetribullis* from Queensland. Zootaxa 3826: 230–254. 10.11646/zootaxa.3826.1.7 24990044

[B56] Khalaji-Pirbalouty V Raupach MJ (2016) DNA barcoding and morphological studies confirm the occurrence of three *Atarbolana* (Crustacea: Isopoda: Cirolanidae) species along the coastal zone of the Persian Gulf and Gulf of Oman. Zootaxa 4200: 153–173. 10.11646/zootaxa.4200.1.7 27988644

[B57] Kilikowska A Wysocka A Burzyński A Kostoski G Rychlińska J Sell J (2013) Patterns of genetic differentiation and population history of endemic isopods (Asellidae) from ancient Lake Ohrid: combining allozyme and mtDNA data. Central European Journal of Biology 8: 854–875. 10.2478/s11535-013-0204-y

[B58] Kim S-J Lee KY Ju S-J (2013) Nuclear mitochondrial pseudogenes in *Austinograeaalayseae* hydrothermal vent crabs (Crustacea: Bythograeidae): effects on DNA barcoding. Molecular Ecology Resources 13: 781–787. 10.1111/1755-0998.12119 23663201

[B59] Kimura M (1980) A simple method for estimating evolutionary rates of base substitutions through comparative studies of nucleotide sequences. Journal of Molecular Evolution 16: 111–120. 10.1007/BF01731581 7463489

[B60] Klossa-Kilia E Kilias G Sfenthourakis S (2005) Increased genetic diversity in Greek populations of the genus *Ligidium* (Crustacea: Isopoda: Oniscidea) revealed by RFLP analysis of mtDNA segments. Contributions to Zoology 74: 255–264. 10.1163/18759866-0740304003

[B61] Kumar S Stecher G Li M Knyaz C Tamura K (2018) MEGA X: Molecular Evolutionary Genetics Analysis across computing platforms. Molecular Biology and Evolution 35: 1547–1549. 10.1093/molbev/msy096 29722887 PMC5967553

[B62] Linsenmair KE (1984) Comparative studies on the social behavior of the desert isopod *Hemilepistusreaumuri* and of a *Porcellio* species. Symposia of the Zoological Society of London 53: 423–453.

[B63] Lopez-Escardo D Paps J de Vargas C Massana R Ruiz-Trillo I del Campo J (2018) Metabarcoding analysis on European coastal samples reveals new molecular metazoan diversity. Scientific Reports 8: e9106. 10.1038/s41598-018-27509-8 PMC6002407 29904074

[B64] Mathes I Strouhal H (1954) Zur Ökologie and Biologie der Ameisenassel *Platyarthrushoffmannseggii* Brandt. Zeitschrift für Morphologie und Ökologie der Tiere 43: 82–93. 10.1007/BF00446232 [In German]

[B65] McClain CR Balk MA Benfield MC Branch TA Chen C Cosgrove J Dove ADM Gaskins LC Helm RR Hochberg FG Lee FB Marshall A McMurray SE Schanche C Stone SN Thaler AD (2015) Sizing ocean giants: patterns of intraspecific size variation in marine megafauna. PeerJ 3: e715. 10.7717/peerj.715 PMC4304853 25649000

[B66] McGaughran A Hogg ID Stevens MI Chadderton WL Winterbourn MJ (2005) Genetic divergence of three freshwater isopod species from southern New Zealand. Journal of Biogeography 33: 23–30. 10.1111/j.1365-2699.2005.01338.x

[B67] Mohrbeck I Raupach MJ Martinez Arbizu P Knebelsberger T Laakmann S (2015) High-throughput sequencing – the key to rapid biodiversity assessment of marine Metazoa? PLoS ONE 10: e0140342. 10.1371/journal.pone.0140342 PMC4610693 26479071

[B68] Morgulis M Coulouris G Rayselis Y Madden TL Agarwala R Schäffer AA (2008) Database indexing for production MegaBLAST searches. Bioinformatics 24: 1757–1764. 10.1093/bioinformatics/btn322 18567917 PMC2696921

[B69] Moriniere J de Arauja BC Lam AW Hausmann A Balke M Schmidt S Hendrich L Doczkal D Farthmann B Arvidsson S Haszprunar G (2016) Species identification in Malaise trap samples by DNA barcoding based on NGS technologies and a scoring matrix. Public Library of Science. PLoS ONE 11: e0155597 10.1371/journal.pone.0155497 PMC4871420 27191722

[B70] Orgiazzi A Dunbar MB Panagos P de Groot GA Lemanceau P (2015) Soil biodiversity and DNA barcodes: opportunities and challenges. Soil Biology and Biochemistry 80: 244–250. 10.1016/j.soilbio.2014.10.014

[B71] Paill W Koblmüller S Friess T Gereben-Krenn B-A Mairhuber C Raupach MJ Zangl L (2021) Relicts from glacial times: The ground beetle *Pterostichusadstrictus* Eschscholtz, 1823 (Coleoptera: Carabidae) in the Austrian Alps. Insects 12: e84. 10.3390/insects12010084 PMC7835791 33478160

[B72] Pansu J De Danieli S Puissant J Gonzalez J-M Gielly L Cordonnier T Zinger L Brun J-J Choler P Taberlet P Cécillon L (2015) Landscape-scale distribution patterns of earthworms inferred from soil DNA. Soil Biology and Chemistry 83: 100–105. 10.1016/j.soilbio.2015.01.004

[B73] Parmakelis A Klossa-Kilia E Kilias G Triantis KA Sfenthourakis S (2008) Increased molecular divergence of two endemic *Trachelipus* (Isopoda, Oniscidea) species from Greece reveals patterns not congruent with current taxonomy. Biological Journal of the Linnean Society 95: 361–370. 10.1111/j.1095-8312.2008.01054.x

[B74] Parmentier T Vanderheyden A Dekoninck W Wenseleers T (2017) Body size in the ant-associated isopod *Platyarthrushoffmannseggii* is host-dependent. Biological Journal of the Linnean Society 121: 305–311. 10.1093/biolinnean/blw052

[B75] Pérez-Moreno JL Balázs G Wilkins B Herczeg G Bracken-Grissom HD (2017) The role of isolation on contrasting phylogeographic patterns in two cave crustaceans. BMC Evolutionary Biology 17: e247. 10.1186/s12862-017-1094-9 PMC5721366 29216829

[B76] Porco D Decaёns T Derharveng L James SW Skarżyński D Erséus C Butt KR Richard B Hebert PDN (2013) Biological invasions in soil: DNA barcoding as a monitoring tool in a multiple taxa survey targeting European earthworms and springtails in North America. Biological Invasions 15: 899–910. 10.1007/s10530-012-0338-2

[B77] Poulakakis N Sfenthourakis S (2008) Molecular phylogeny and phylogeography of the Greek populations of the genus *Orthometopon* (Isopoda, Oniscidea) based on mitochondrial DNA sequences. Zoological Journal of the Linnean Society 152: 707–715. 10.1111/j.1096-3642.2007.00378.x

[B78] Ratnasingham S Hebert PDN (2007) BOLD: The Barcode of Life Data Systems. Molecular Ecology Notes 7: 355–364. 10.1111/j.1471-8286.2007.01678.x 18784790 PMC1890991

[B79] Ratnasingham S Hebert PDN (2013) A DNA-based registry for all animal species: the Barcode Index Number (BIN) system. PLoS ONE 8: e66213. 10.1371/journal.pone.0066213 PMC3704603 23861743

[B80] Raupach MJ (2005) Die Bedeutung von Landasseln als Beutetiere für Insekten und andere Arthropoden. Entomologie heute 17: 3–12. [In German]

[B81] Raupach MJ Held C Wägele JW (2004) Multiple colonization of the deep sea by the Asellota (Crustacea: Peracarida: Isopoda). Deep-Sea Research II – Topical Studies in Oceanography 51: 1787–1795. 10.1016/j.dsr2.2004.06.035

[B82] Raupach MJ Thatje S (2006) New records of the rare shrimp parasite *Zonophryxusquinquedens* Barnard, 1913 (Crustacea, Isopoda, Dajidae): ecological and phylogenetic implications. Polar Biology 29: 439–443. 10.1007/s00300-005-0069-2

[B83] Raupach MJ Mayer C Malyutina M Wägele JW (2009) Multiple origins of deep-sea Asellota (Crustacea: Isopoda) from shallow waters revealed by molecular data. Proceedings of the Royal Society of London Series B 276: 799–808. 10.1098/rspb.2008.1063 19033145 PMC2664356

[B84] Raupach MJ Bininda-Emonds ORP Knebelsberger T Laakmann S Pfaener J Leese F (2014) Phylogeographic analysis of *Ligiaoceanica* (Crustacea: Isopoda) reveals two deeply divergent mitochondrial lineages. Biological Journal of the Linnean Society 112: 16–30. 10.1111/bij.12254

[B85] Raupach MJ Barco A Steinke D Beermann J Laakmann S Mohrbeck I Neumann H Kihara TC Pointner K Radulovici A Segelken-Voigt A Weese C Knebelsberger T (2015) The application of DNA barcodes for the identification of marine crustaceans from the North Sea and adjacent regions. PLoS ONE 10: e0139421. 10.1371/journal.pone.0139421 PMC4587929 26417993

[B86] Raupach MJ Hannig K Moriniére J Hendrich L (2016) A DNA barcode library for ground beetles (Insecta: Coleoptera: Carabidae) of Germany: The genus *Bembidion* Latreille, 1802 and allied taxa. ZooKeys 592: 121–141. 10.3897/zookeys.592.8316 PMC4926639 27408547

[B87] Raupach MJ Hannig K Moriniére J Hendrich L (2018) A DNA barcode library for ground beetles of Germany: The genus *Amara* Bonelli, 1810 (Insecta: Coleoptera: Carabidae). ZooKeys 759: 57–80. 10.3897/zookeys.759.24129 PMC5968077 29853775

[B88] Rigaud T Moreau J Juchault P (1999) *Wolbachia* infection in the terrestrial isopod *Oniscusasellus*: sex ratio distortion and effect on fecundity. Heredity 83: 469–475. 10.1038/sj.hdy.6885990 10583549

[B89] Rota N Canedoli C Ferre C Ficetola GF Guerrieri A Padoa-Schioppa E (2020) Evaluation of soil biodiversity in Alpine habitats through eDNA metabarcoding and relationships with environmental features. Forests 11: e738. https://10.3390/f11070738

[B90] Saitou N Nei M (1987) The neighbor-joining method: a new method for reconstructing phylogenetic trees. Molecular Biology and Evolution 4: 406–425. 3447015 10.1093/oxfordjournals.molbev.a040454

[B91] Santamaria CA Bluemel JK Bunbury N Curran M (2017) Cryptic biodiversity and phylogeographic patterns of Seychellois *Ligia* isopods. PeerJ 5: e3894. 10.7717/peerj.3894 PMC5633021 29018626

[B92] Santamaria CA (2019) Molecular taxonomy of endemic coastal *Ligia* isopods from the Hawaiian Islands: re-description of *L.hawaiensis* and description of seven novel cryptic species. PeerJ 7: e7531. 10.7717/peerj.7531 PMC6698373 31435494

[B93] Schizas NV (2012) Misconceptions regarding nuclear mitochondrial pseudogenes (numts) may obscure detection of mitochondrial novelties. Aquatic Biology 17: 91–96. 10.3354/ab00478

[B94] Schmalfuss H (1984) Eco-morphological strategies in terrestrial isopods. Symposia of the Zoological Society of London 53: 49–63.

[B95] Schmidt C (2008) Phylogeny of the terrestrial Isopoda (Oniscidea): a review. Arthropod Systematics & Phylogeny 66: 191–226.

[B96] Schmölzer K (1964) Ordnung Isopoda (Landasseln). In: Franz H (Ed.) Bestimmungsbücher zur Bodenfauna Europas, Lieferung 4/5.Akademie-Verlag, Berlin, 1–486. [In German]

[B97] Sfenthourakis S Taiti S (2015) Patterns of taxonomic diversity among terrestrial isopods. In: Taiti S Hornung E Štrus J Bouchon D (Eds) Trends in Terrestrial Isopod Biology.ZooKeys 515: 13–25. 10.3897/zookeys.515.9332 PMC4525032 26261437

[B98] Shokralla S Spall JL Gibson JF Hajibabaei M (2012) Next-generation sequencing technologies for environmental DNA research. Molecular Ecology 21: 1794–1805. 10.1111/j.1365-294X.2012.05538.x 22486820

[B99] Singer C Bello NM Snyder BA (2012) Characterizing prevalence and ecological impact of non-native terrestrial isopods (Isopoda, Oniscidea) in tallgrass prairie. Crustaceana 85: 1499–1511. 10.1163/15685403-00003126

[B100] Slabber S Chwon SL (2002) The first record of a terrestrial crustacean, *Porcellioscaber* (Isopoda, Porcellionidae), from sub-Antarctic Marion Island. Polar Biology 25: 855–858. 10.1007/s00300-002-0420-9

[B101] Smit NJ Bruce NL Hadfield KA (2014) Global diversity of fish parasitic isopod crustaceans of the family Cymothoidae. International Journal for Parasitology: Parasites and Wildlife 3: 188–197. 10.1016/j.ijppaw.2014.03.004 25180163 PMC4145142

[B102] Špaldoňová A Frouz J (2014) The role of *Armadillidiumvulgare* (Isopoda: Oniscidea) in litter decomposition and soil organic matter stabilization. Applied Soil Ecology 83: 186–192. 10.1016/j.apsoil.2014.04.012

[B103] Spelda J Reip HS Oliveira-Biener U Melzer RR (2011) Barcoding Fauna Bavarica: Myriapoda – a new contribution to DNA-based identifications of centipedes and millipedes. In: Mesibov R Short M (Eds) Proceedings of the 15th International Congress of Myriapodology, 18–22 July 2011, Brisbane, Australia.ZooKeys 156: 123–139. 10.3897/zookeys.156.2176 PMC3253575 22303099

[B104] Staats M Arulandhu AJ Gravendeel B Horst-Jensen A Scholtens I Peelen T Prins TW Kok E (2016) Advances in DNA metabarcoding for food and wildlife forensic species identifi­cation. Analytical and Bioanalytical Chemistry 408: 4615–4630. 10.1007/s00216-016-9595-8 27178552 PMC4909793

[B105] Sun X Bedos A Deharveng L (2018) Unusually low genetic divergence at COI barcode locus between two species of intertidal *Thalassaphorura* (Collembola: Onychiuridae). PeerJ 6: e5021. 10.7717/peerj.5021 PMC6011825 29938135

[B106] Sworobowicz L Grabowski M Mamos T Burzyński A Kilikowska A Sell J Wysocka A (2015) Revisiting the phylogeography of *Asellusaquaticus* in Europe: insights into cryptic diversity and spatiotemporal diversification. Freshwater Biology 60: 1824–1840. 10.1111/fwb.12613

[B107] Taberlet P Coissac E Pompanon F Brochmann C Willerslev E (2012) Towards next-generation biodiversity assessment using DNA metabarcoding. Molecular Ecology 21: 2045–2050. 10.1111/j.1365-294X.2012.05470.x 22486824

[B108] Valentini A Pompanon F Taberlet P (2009) DNA barcoding for ecologists. Trends in Ecology and Evolution 24: 110–117. 10.1016/j.tree.2008.09.011 19100655

[B109] Verovnik R Sket B Trontelj P (2004) Phylogeography of subterranean and surface populations of water lice *Asellusaquaticus* (Crustacea: Isopoda). Molecular Ecology 13: 1519–1532. 10.1111/j.1365-294X.2004.02171.x 15140095

[B110] Verovnik R Sket B Trontelj P (2005) The colonization of Europe by the freshwater crustacean *Asellusaquaticus* (Crustacea; Isopoda) proceeded from ancient refugia and was directed by habitat connectivity. Molecular Ecology 14: 4355–4369. 10.1111/j.1365-294X.2005.02745.x 16313598

[B111] Wägele JW (1989) Evolution und phylogenetisches System der Isopoda: Stand der Forschung und neue Erkenntnisse. Zoologica 140: 1–262. [In German]

[B112] Werren JH Baldo L Clark ME (2008) *Wolbachia*: master manipulators of invertebrate biology. Nature Reviews 6: 741–751. 10.1038/nrmicro1969 18794912

[B113] Williams JD Boyko CB (2012) The global diversity of parasitic isopods associated with crustacean hosts (Isopoda: Bopyroidea and Cryptoniscoidea). PLoS ONE 7: e35350. 10.1371/journal.pone.0035350 PMC3338838 22558143

[B114] Wilson GDF Hessler RR (1987) Speciation in the deep sea. Annual Review of Ecology and Systematics 18: 185–207. 10.1146/annurev.es.18.110187.001153

[B115] Wilson GDF (2008) Global diversity of isopod crustaceans (Crustacea; Isopoda) in freshwater. Hydrobiologia 595: 231–240. 10.1007/s10750-007-9019-z

[B116] Young MR Behan-Pelletier VM Hebert PDN (2012) Revealing the hyperdiverse mite fauna of subarctic Canada through DNA Barcoding. PLoS ONE 7: e48755. 10.1371/journal.pone.0048755 PMC3487733 23133656

[B117] Young MR Moraza ML Ueckermann E Heylen D Baardsen LF Lima-Barbero JF Gal S Gavish-Regev Gottlieb Y Roy L Recht E El Adouzi M Palevsky E (2019) Linking morphological and molecular taxonomy for the identification of poultry house, soil, and nest dwelling mites in the Western Palearctic. Scientific Reports 9: e5784. 10.1038/s41598-019-41958-9 PMC6453913 30962473

[B118] Zhang Z Schwartz S Wagner L Miller W (2000) A greedy algorithm for aligning DNA sequences. Journal of Computational Biology 7: 203–214. 10.1089/10665270050081478 10890397

[B119] Zimmermann BL Campos-Filho IS Deprá M Araujo PB (2015) Taxonomy and molecular phylogeny of the Neotropical genus *Atlantoscia* (Oniscidea, Philosciidae): DNA barcoding and description of two new species. Zoological Journal of the Linnean Society 174: 702–717. 10.1111/zoj.12256

[B120] Zimmermann BL Campos-Filho IS Araujo PB (2018a) Integrative taxonomy reveals a new genus and new species of Philosciidae (Crustacea: Isopoda: Oniscidea) from the Neotropical region. Canadian Journal of Zoology 96: 473–485. 10.1139/cjz-2017-0289

[B121] Zimmermann BL Campos-Filho IS Cardoso GM Santos S Aguiar JO Araujo PB (2018b) Two new species of *Atlantoscia* Ferrara & Taiti, 1981 (Isopoda: Oniscidea: Philosciidae) from southern Brazil. Zootaxa 4482: 551–565. 10.11646/zootaxa.4482.3.7 30313814

