# Supplementary material for: ﻿Surprisingly high genetic divergence of the mitochondrial DNA barcode fragment (COI) within Central European woodlice species (Crustacea, Isopoda, Oniscidea)
Source: Zookeys. 2022 Jan 20;1082:103–25. doi: 10.3897/zookeys.1082.69851 (PMC8794987; doi:10.3897/zookeys.1082.69851)
Supplement: Supplementary material 1 — Barcode analysis using the BOLD workbench [file zookeys-1082-103-s001.docx]

| Suborder | Family | Species | Habitat^1^ | *n* | BIN | Mean ISD | Max. ISD | Nearest species (NS) | Distance to NS |
| --- | --- | --- | --- | --- | --- | --- | --- | --- | --- |
| Asellota |  |  |  |  |  |  |  |  |  |
|  | **Asellidae** |  |  |  |  |  |  |  |  |
|  |  | *Asellus aquaticus* (Linnaeus, 1758) | F | 41 | ACF1266, AEC4774, AAA1970 | **4.25** | **13.37** | *Proasellus coxalis* | 22.78 |
|  |  | *Proasellus cavaticus* (Leydig, 1871) | F | 8 | ADX3790, ADW6988, ADX4659 | 1.61 | **2.95** | *Asellus aquaticus* | 24.61 |
|  |  | *Proasellus coxalis* (Dollfus, 1892) ^4^ | F | 13 | ACI1746, ACH7545 | **2.81** | **5.78** | *Asellus aquaticus* | 22.78 |
|  |  | *Proasellus meridianus* (Racovitza, 1919) ^4^ | F | 2 | ACH8161 | 0 | 0 | *Asellus aquaticus* | 25.04 |
|  | **Janiridae** |  |  |  |  |  |  |  |  |
|  |  | *Jaera sarsi* Valkanov, 1936^3,4^ | F | 7 | ADK8576 | 0.06 | 0.15 | *Armadillidium nasatum* | 29.76 |
| Oniscidea |  |  |  |  |  |  |  |  |  |
|  | **Armadillidiidae** |  |  |  |  |  |  |  |  |
|  |  | *Armadillidium album* Dollfus, 1887 | T | 1 | ADK8705 | n. a. | n. a. | *Armadillidium pictum* | 16.89 |
|  |  | *Armadillidium granulatum* Brandt, 1833 | T | 2 | ADK9815 | 0.18 | 0.18 | *Armadillidium versicolor* | 12.57 |
|  |  | *Armadillidium nasatum* Budde-Lund, 1885 | T | 13 | AAU8053 | 0.02 | 0.15 | *Armadillidium opacum* | 16.83 |
|  |  | *Armadillidium opacum* (C. Koch, 1841) | T | 8 | AAV4460 | 0.45 | 1.01 | *Armadillidium vulgare* | 14.78 |
|  |  | *Armadillidium pictum* Brandt, 1833 | T | 8 | AAV9852 | 1.35 | 2.18 | *Armadillidium album* | 16.89 |
|  |  | *Armadillidium pulchellum* (Zenker, 1798) | T | 1 | ADF8224 | n. a. | n. a. | *Armadillidium album* | 18.37 |
|  |  | *Armadillidium versicolor* Stein, 1859^4^ | T | 2 | AAV9853 | 0.77 | 0.77 | *Armadillidium granulatum* | 12.57 |
|  |  | *Armadillidium vulgare* (Latreille, 1804) | T | 28 | AAE6611, AAH4108, AAH4111, AAU1529 | **3.76** | **6.44** | *Armadillidium opacum* | 14.78 |
|  | **Cylisticidae** |  |  |  |  |  |  |  |  |
|  |  | *Cylisticus convexus* (De Geer, 1778) | T | 13 | AAU8054 | 0.35 | 0.77 | *Armadillidium vulgare* | 20.59 |
|  | **Ligiidae** |  |  |  |  |  |  |  |  |
|  |  | *Ligia italica* Fabricius, 1798* | T | 2 | ACQ8250 | 0 | 0 | *Porcellionides sexfasciatus* | 20.94 |
|  |  | *Ligia oceanica* (Linnaeus, 1767) | T | 11 | AAJ2795 | 0.03 | 0.15 | *Trachelipus rathkii* | 23.84 |
|  |  | *Ligidium germanicum* Verhoeff, 1901 | T | 8 | AAE0735 | 0.24 | 0.62 | *Ligidium hypnorum* | 24.78 |
|  |  | *Ligidium hypnorum* (Cuvier, 1792) | T | 13 | AAF5619 | 0.33 | 0.77 | *Ligidium germanicum* | 24.78 |
|  | **Mesoniscidae** |  |  |  |  |  |  |  |  |
|  |  | *Mesoniscus alpicola* (Heller, 1858) | T | 1 | ACT8509 | n. a. | n. a. | *Armadillidium vulgare* | 23.01 |
|  | **Oniscidae** |  |  |  |  |  |  |  |  |
|  |  | *Oniscus asellus* Linnaeus, 1758 | T | 33 | ADM8743, ADM8116, ADK9123 | 2.12 | **5.63** | *Porcellio montanus* | 24.2 |
|  | **Philosciidae** |  |  |  |  |  |  |  |  |
|  |  | *Lepidoniscus minutus* (C. Koch, 1838) | T | 4 | AAV7783 | 0.08 | 0.16 | *Armadillidium vulgare* | 20.41 |
|  |  | *Lepidoniscus pruinosus* (Carl, 1908) | T | 1 | ADF6888 | n. a. | n. a. | *Armadillidium vulgare* | 22.39 |
|  |  | *Philoscia affinis* Verhoeff, 1908 | T | 3 | ADM8125, AAY1058 | **3.63** | **5.44** | *Philoscia muscorum* | 19.64 |
|  |  | *Philoscia muscorum* (Scopoli, 1763) | T | 38 | AAH4103, AAH4104 | 0.3 | **2.98** | *Philoscia affinis* | 19.64 |
|  | **Platyarthridae** |  |  |  |  |  |  |  |  |
|  |  | *Platyarthrus hoffmannseggii* Brandt, 1833 | T | 33 | AAV8050, AAV8051, ADK9658 | **9.4** | **29.35** | *Trachelipus nodulosus* | 28.7 |
|  | **Porcellionidae** |  |  |  |  |  |  |  |  |
|  |  | *Porcellio montanus* Budde-Lund, 1885 | T | 6 | ADR0694, ADM7742 | 1.26 | **3.81** | *Porcellio spinicornis* | 21.15 |
|  |  | *Porcellio monticola* Lereboullet, 1853 | T | 2 | ADF5454 | 0 | 0 | *Porcellio spinicornis* | 18.71 |
|  |  | *Porcellio scaber* Latreille, 1804 | T | 57 | AAC3755, AAZ0248, ABA5892, ADK8850, ADM8147 | **2.58** | **12.16** | *Armadillidium versicolor* | 15.58 |
|  |  | *Porcellio spinicornis* Say, 1818 | T | 6 | ADF7011, ADI3596 | **3.01** | **5.13** | *Porcellio scaber* | 18.52 |
|  |  | *Porcellionides pruinosus* (Brandt, 1833) | T | 2 | AAH4110 | 0.15 | 0.15 | *Trachelipus rathkii* | 22.09 |
|  |  | *Porcellionides sexfasciatus* (Budde-Lund, 1885)* | T | 4 | ADK9664 | 0 | 0 | *Porcellio spinicornis* | 18.91 |
|  |  | *Porcellium conspersum* (C. Koch, 1841) | T | 7 | AAV7038 | 0.22 | 0.61 | *Porcellium fiumanum sal.* | 18.88 |
|  |  | *Porcellium fiumanum* (Verhoeff, 1901) | T | 2 | AAV7039 | 0 | 0 | *Porcellium conspersum* | 18.88 |
|  |  | *Protracheoniscus politus* (C. Koch, 1841) | T | 7 | AAX8613 | 0.26 | 1.08 | *Porcellio monticola* | 18.86 |
|  | **Trachelipodidae** |  |  |  |  |  |  |  |  |
|  |  | *Trachelipus nodulosus* (C. Koch, 1838) | T | 5 | ADM7095 | 0.06 | 0.15 | *Trachelipus rathkii* | 15.3 |
|  |  | *Trachelipus rathkii* (Brandt, 1833) | T | 16 | AAH4102, ADK8699, ADK8533, ADM8087, ADM8088, ADF6188 | **6.89** | **16.59** | *Trachelipus nodulosus* | 15.3 |
|  |  | *Trachelipus ratzeburgii* (Brandt, 1833) | T | 17 | AAV6691 | 0.07 | 0.31 | *Trichoniscus pusillus* | 18.7 |
|  | **Trichoniscidae** |  |  |  |  |  |  |  |  |
|  |  | *Androniscus roseus* (C. Koch, 1838) | T | 2 | ADE9981 | 0.31 | 0.31 | *Protracheoniscus politus* | 24.93 |
|  |  | *Haplophthalmus danicus* Budde-Lund, 1880 | T | 6 | AAU3256 | 1.06 | 1.71 | *Haplophthalmus montivagus* | 23.63 |
|  |  | *Haplophthalmus mariae* (Strouhal, 1953)^4^ | T | 2 | ADQ8421 | 0 | 0 | *Haplophthalmus montivagus* | 21.17 |
|  |  | *Haplophthalmus mengii* Zaddach, 1844 | T | 3 | ADM7489 | 0.31 | 0.46 | *Haplophthalmus montivagus* | 23.7 |
|  |  | *Haplophthalmus montivagus* Verhoeff, 1941 | T | 12 | AAY2109 | 0.1 | 0.46 | *Haplophthalmus mariae* | 21.17 |
|  |  | *Hyloniscus riparius* (C. Koch, 1838) | T | 22 | AAV6495 | 0.11 | 0.46 | *Armadillidium album* | 21.45 |
|  |  | *Trichoniscoides helveticus* (Carl, 1908) | T | 23 | ADM7247, ADM7248, ADM7249 | 1.07 | **5.46** | *Androniscus roseus* | 27.14 |
|  |  | *Trichoniscus pusillus* Brandt, 1833 | T | 22 | AAN7523, AAZ1993 | **6.8** | **13.47** | *Trachelipus ratzeburgii* | 18.7 |
|  | **Tylidae** |  |  |  |  |  |  |  |  |
|  |  | *Tylos ponticus* Grebnitzky, 1874* | T | 1 | ADK9948 | n. a. | n. a. | *Cylisticus convexus* | 24.27 |

**Remarks**

1: Habitat classification: F = Freshwater, T = Terrestrial.

2: Astrisks (*) indicate species not recorded for Germany so far.

3: Following Tobias et al. (2005), the species name *Jaera istri* Veuille, 1979 was treated as younger synonym of *Jaera sarsi* Valkanov, 1936.

4: Neozoic species for Germany (see Grünwald 2016).

**References**

Grünwald M (2016) Rote Liste und Gesamtartenliste der Landasseln und Wasserasseln (Isopoda: Oniscidea et Asellota) Deutschlands, 1. Fassung, Stand November 2011. In: Bundesamt für Naturschutz (BfN) (Hrsg.): Rote Liste gefährdeter Tiere, Pflanzen und Pilze Deutschlands; Band 4: Wirbellose Tiere (Teil 2). Naturschutz und Biologische Vielfalt 70 (4): 349-363. In German.

Tobias W, Wegmann A, Bernerth H (2005) *Jaera istri* oder *Jaera sarsi*? – Zum taxonomischen Status der “Donauassel” (Isopoda, Asellota: Janiridae). Faunistisch-ökologische Untersuchungen des Forschungsinstitutes Senckenberg im hessischen Main. Wiesbaden. Landesamt für Umwelt und Geologie: 5–14. In German.

Valkanov A (1936) Notizen über die Brackwässer Bulgariens. II. Versuch einer hydrographischen und biologischen Erforschung derselben. Godishnik na Sofiiskiyauniversitet. Fiziko-matematicheski fakultet 42: 209-341. In Bulgarian.

Veuille M (1979) L´evolution du genera *Jaera* Leach (Isopodes; Asellotes) et ses rapports avec l´histoire de la Méditerranée. Bijdragen tot de Dierkunde 49 : 195-217. In French.
