# Supplementary figures and images for: ﻿Surprisingly high genetic divergence of the mitochondrial DNA barcode fragment (COI) within Central European woodlice species (Crustacea, Isopoda, Oniscidea)
Source: Zookeys. 2022 Jan 20;1082:103–25. doi: 10.3897/zookeys.1082.69851 (PMC8794987; doi:10.3897/zookeys.1082.69851)

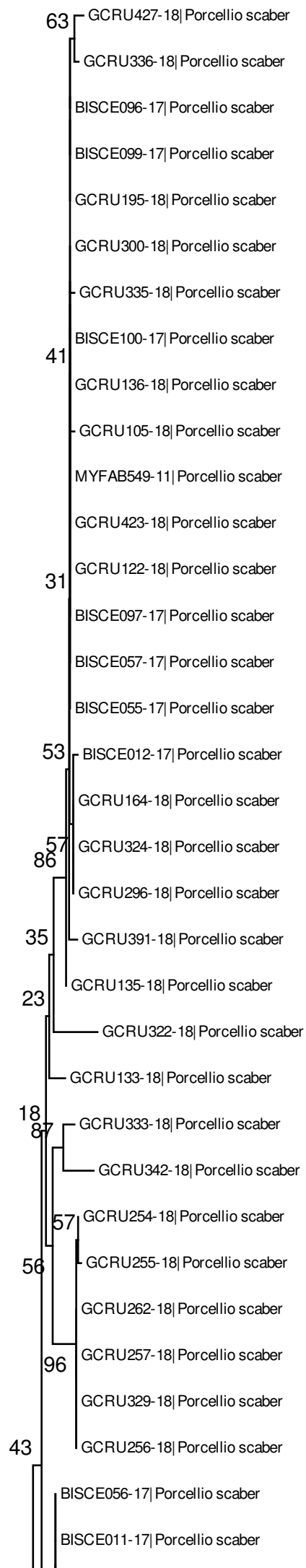

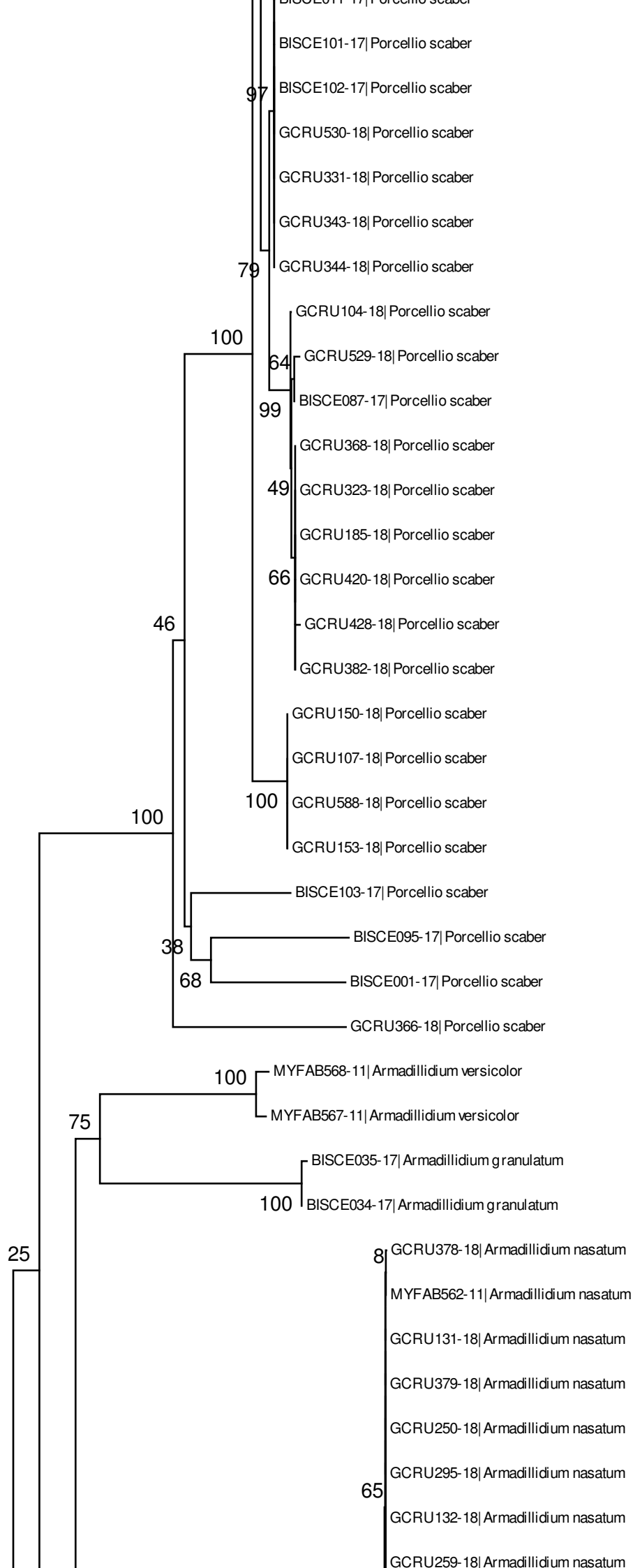

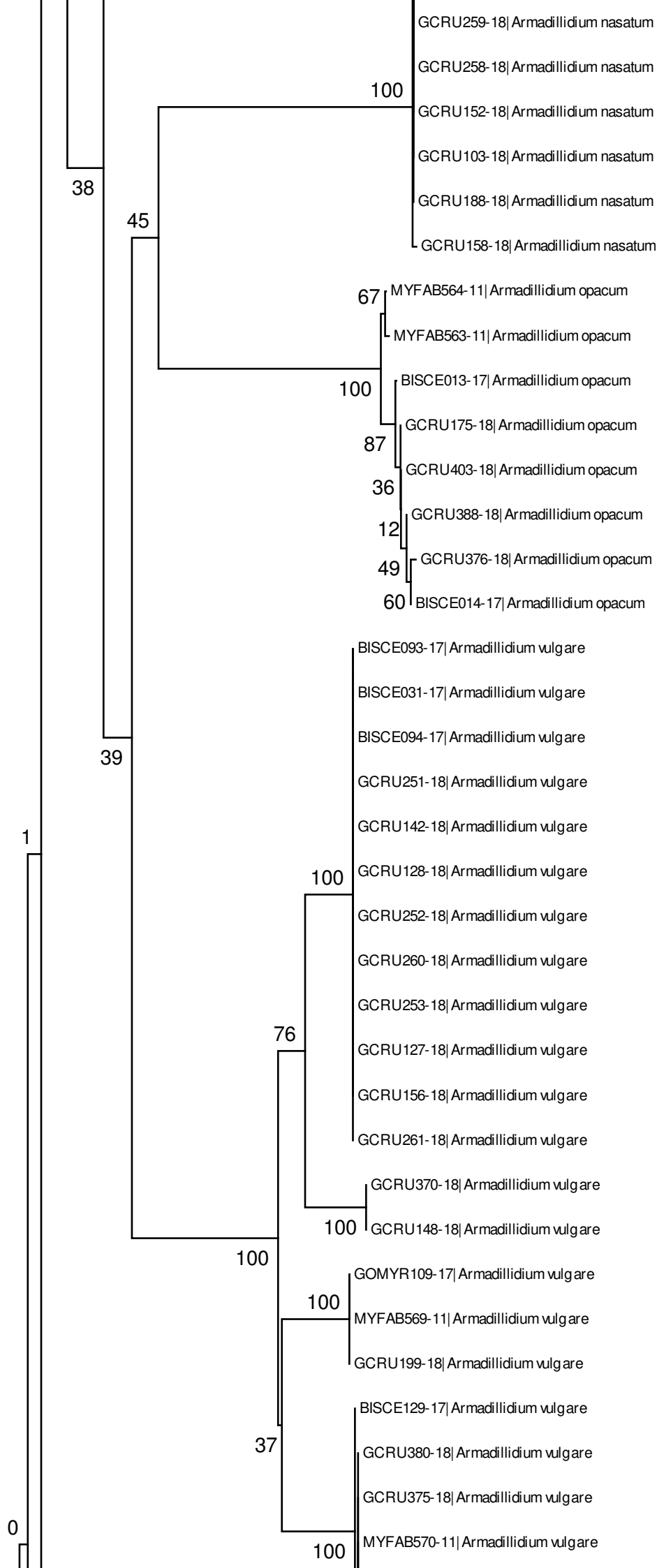

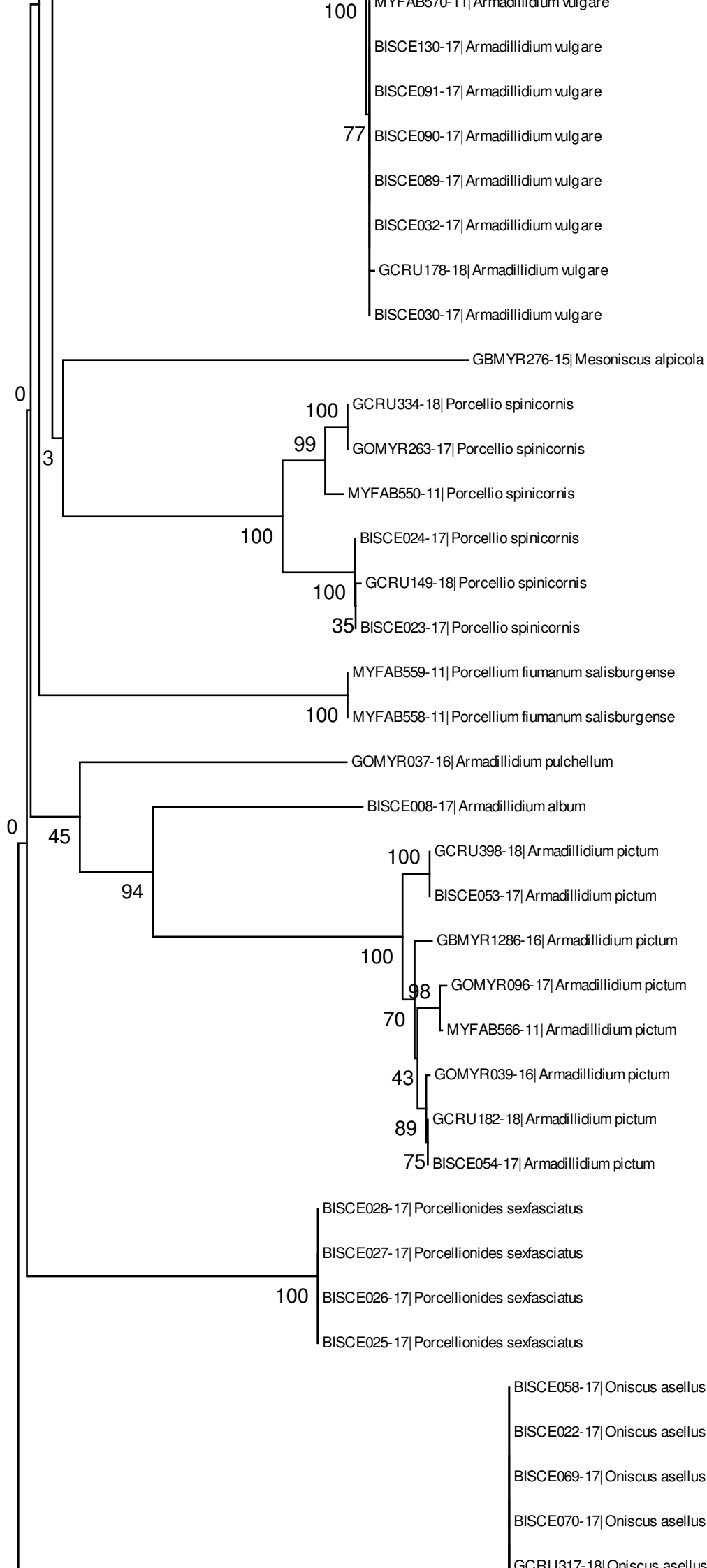

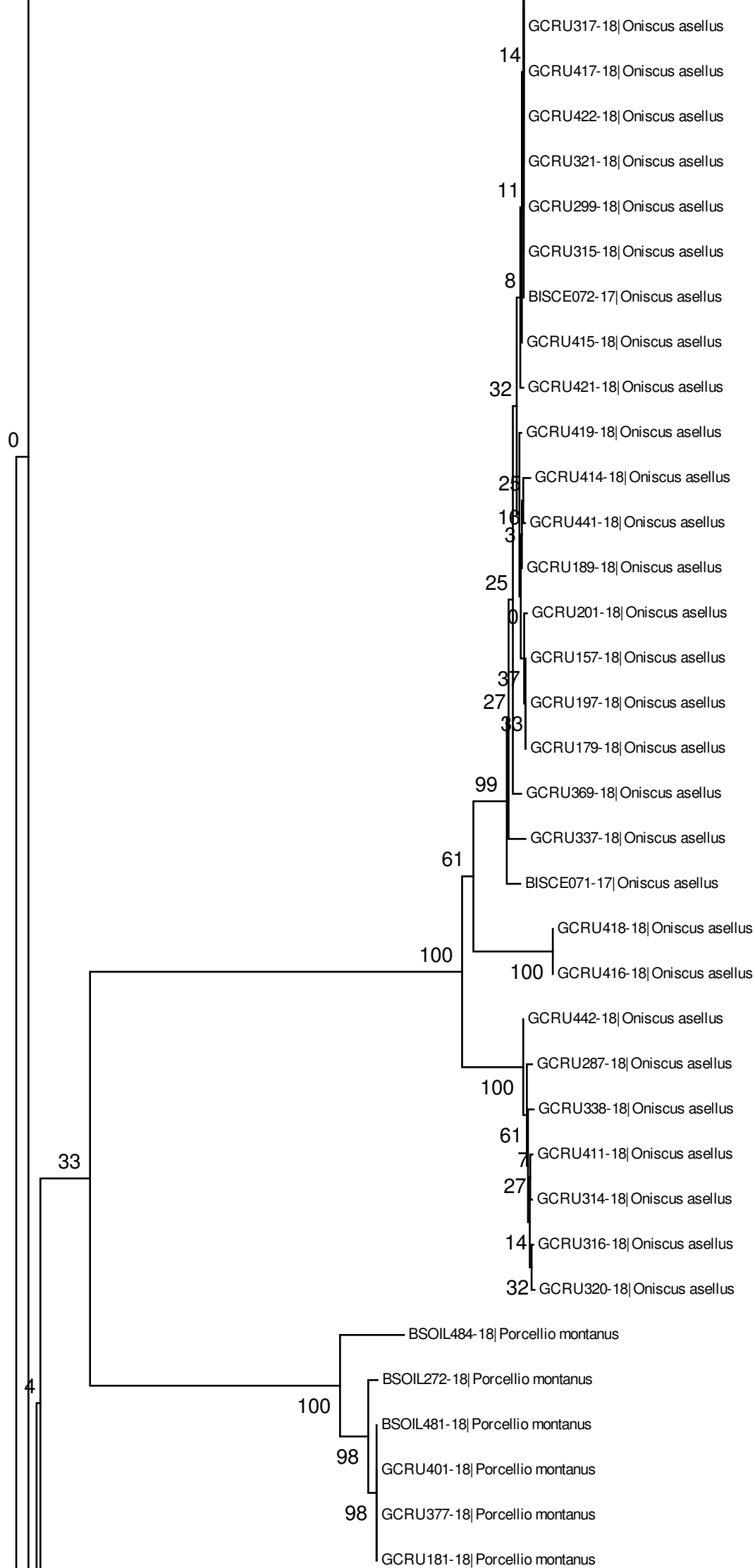

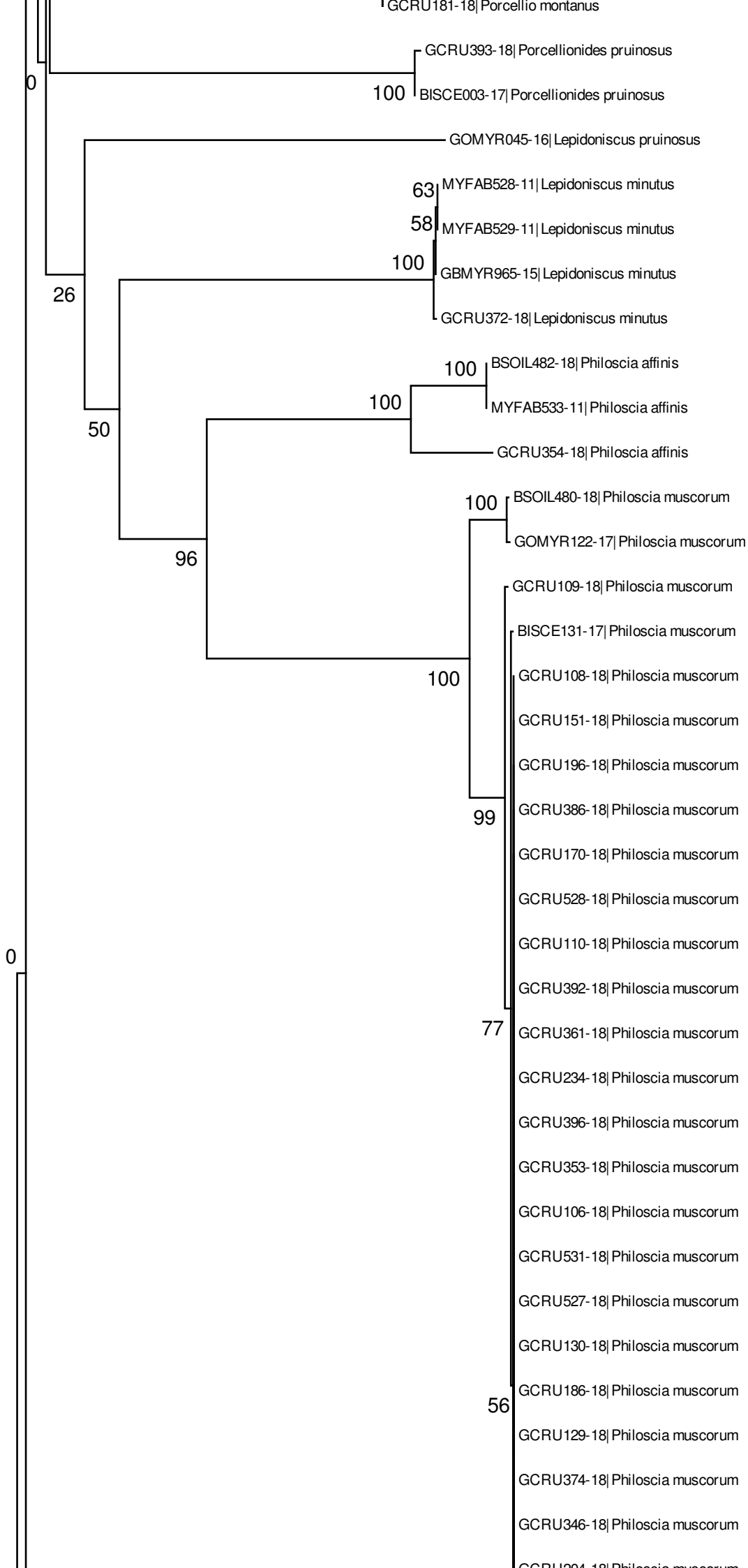

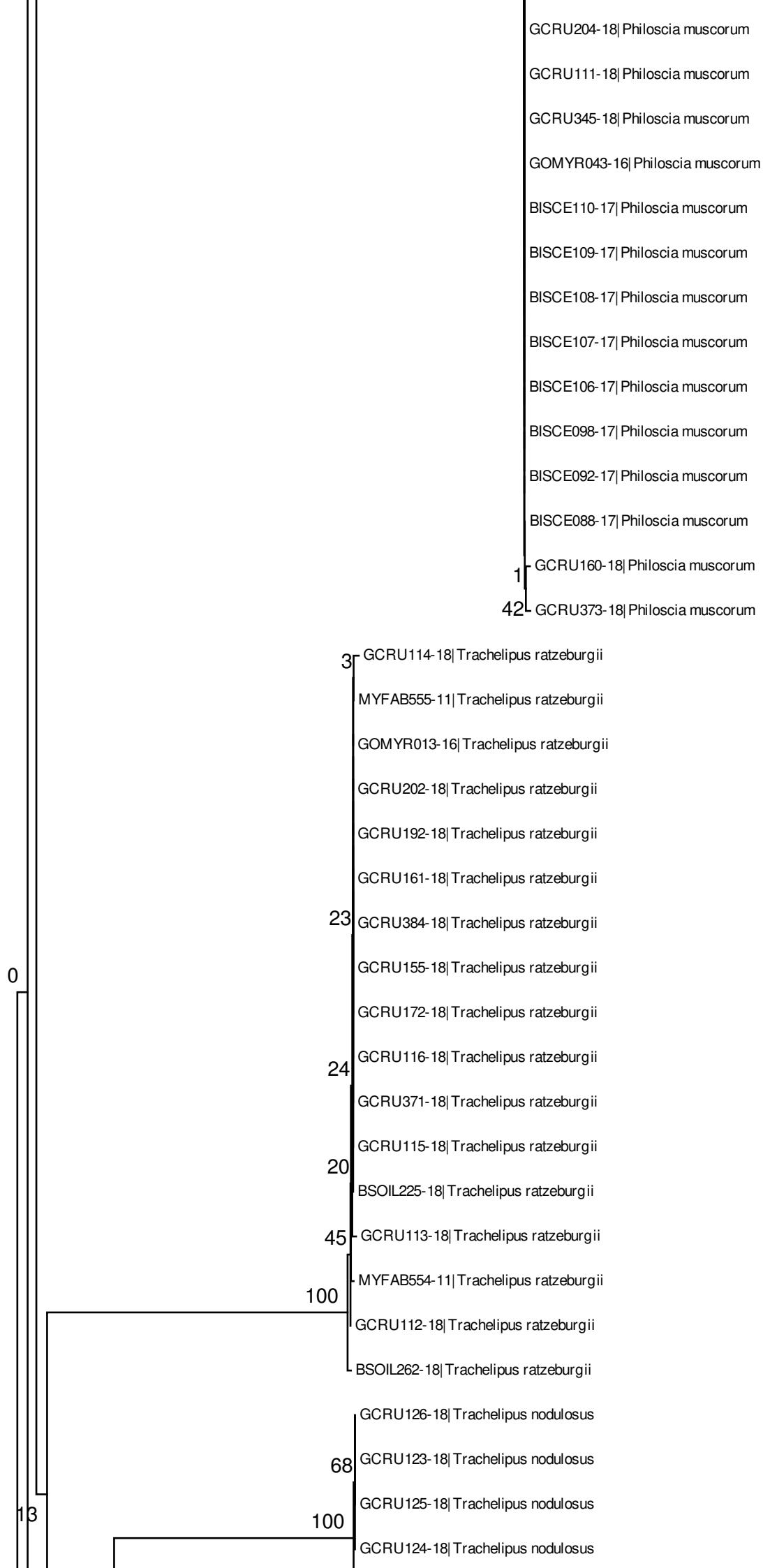

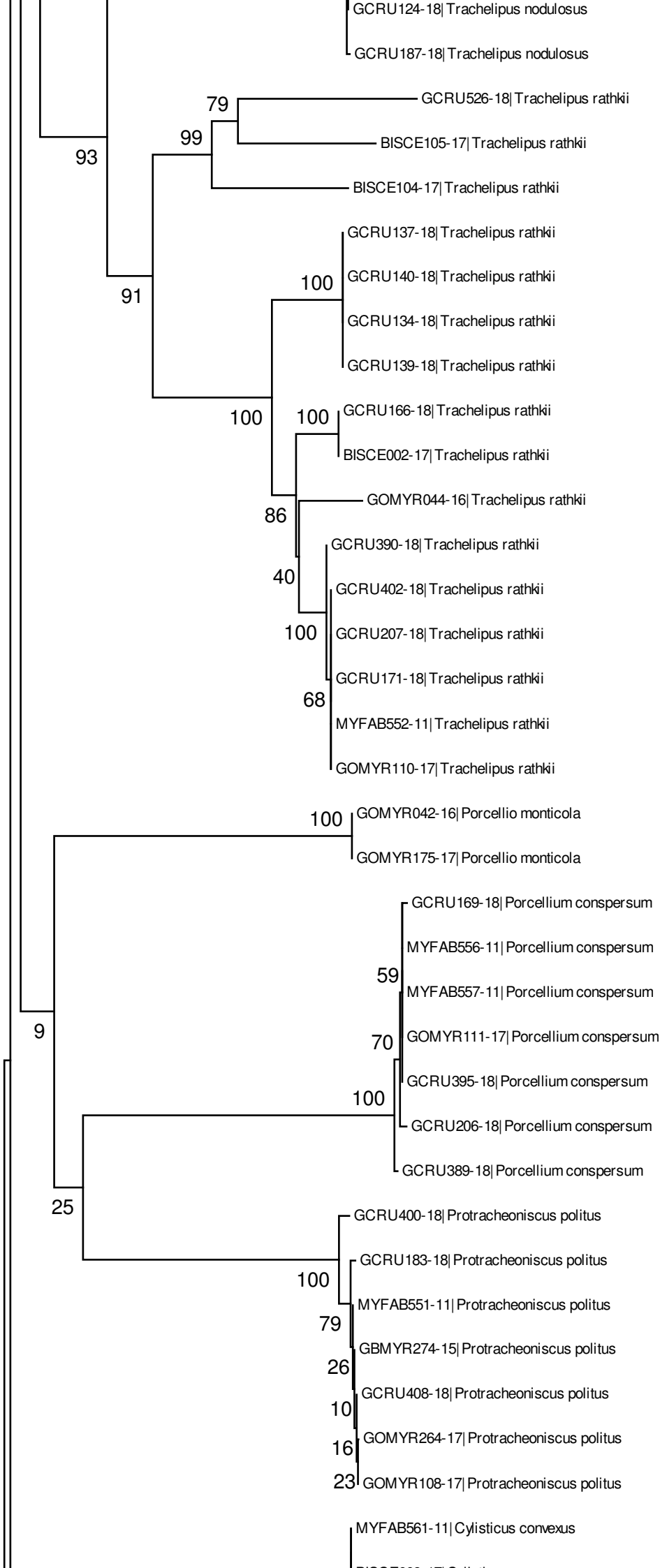

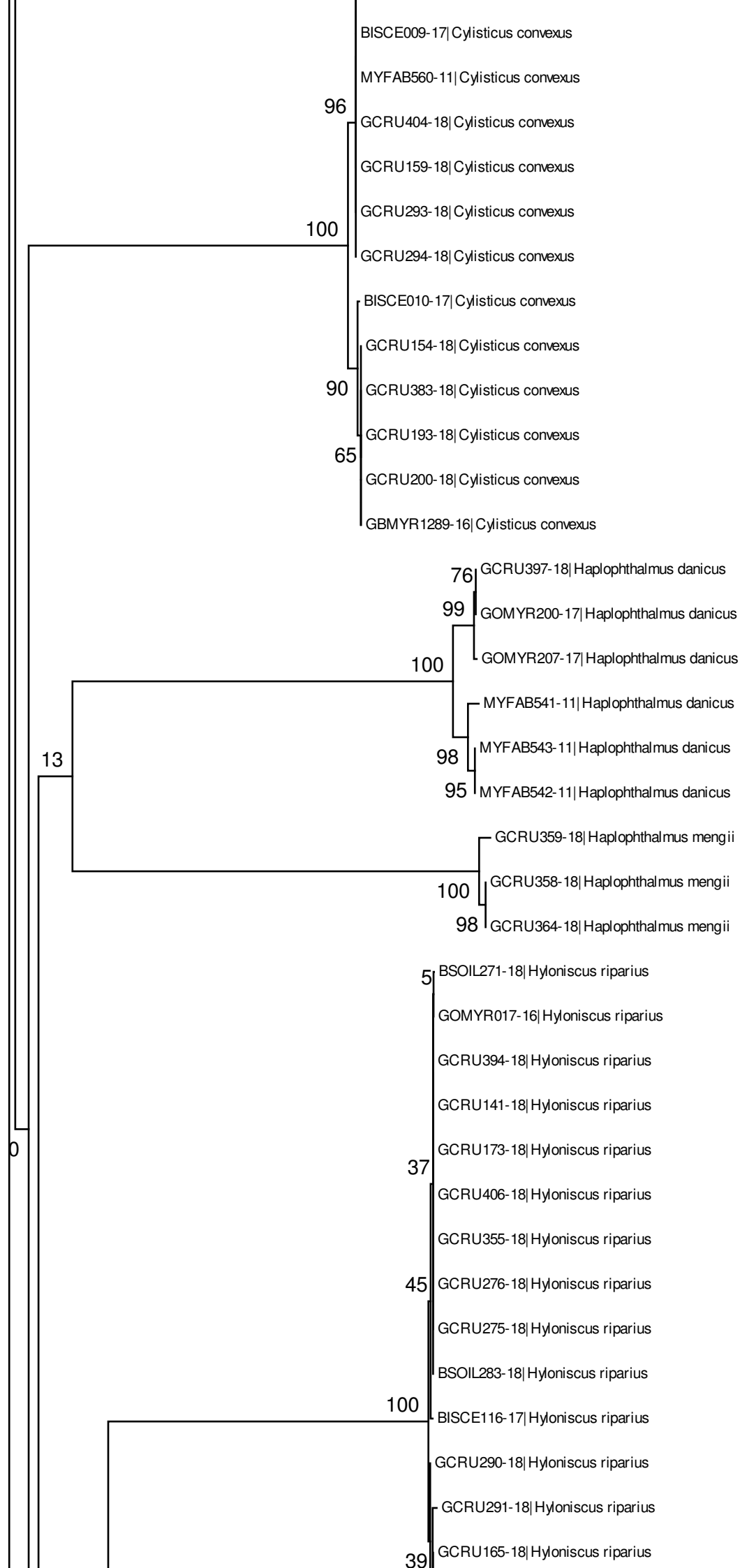

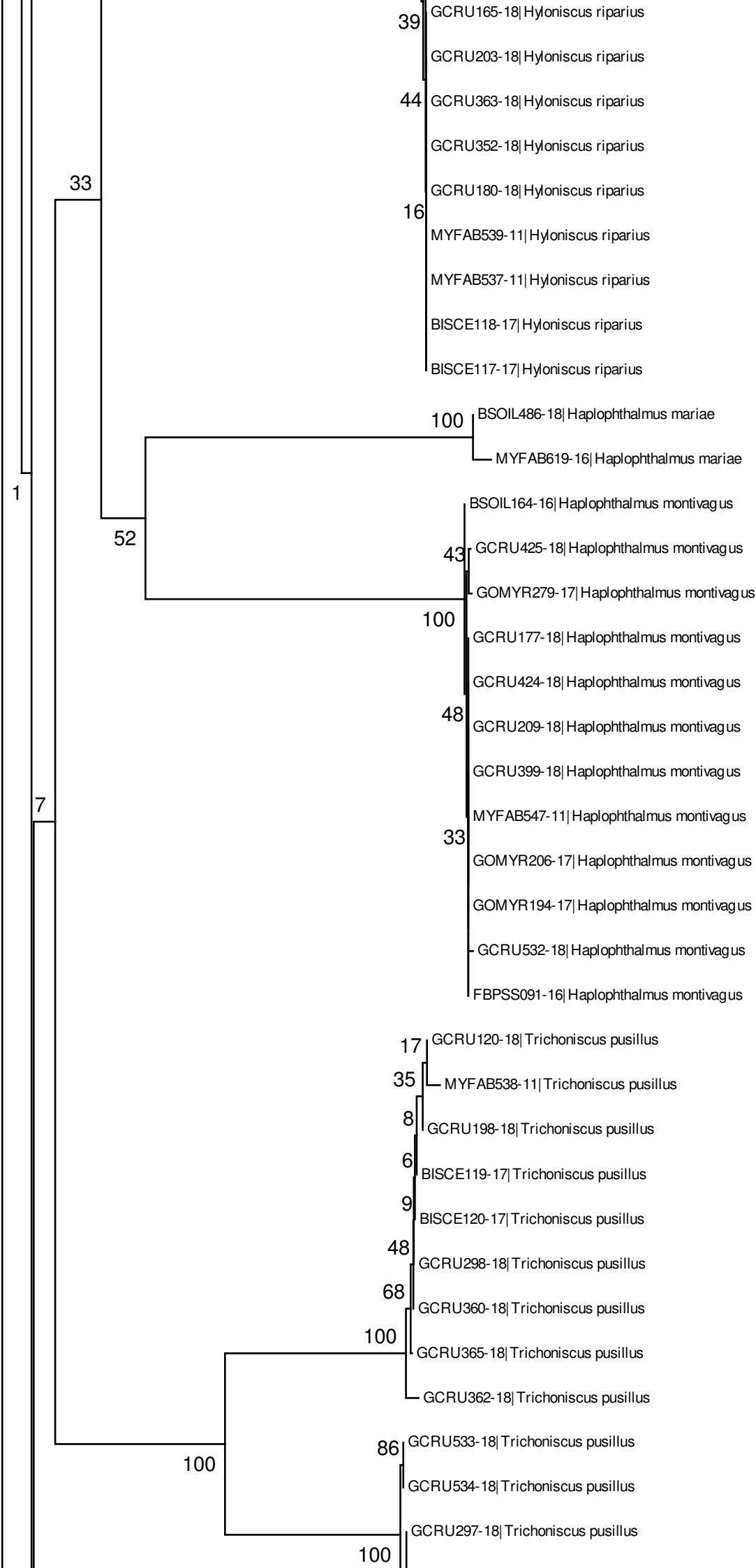

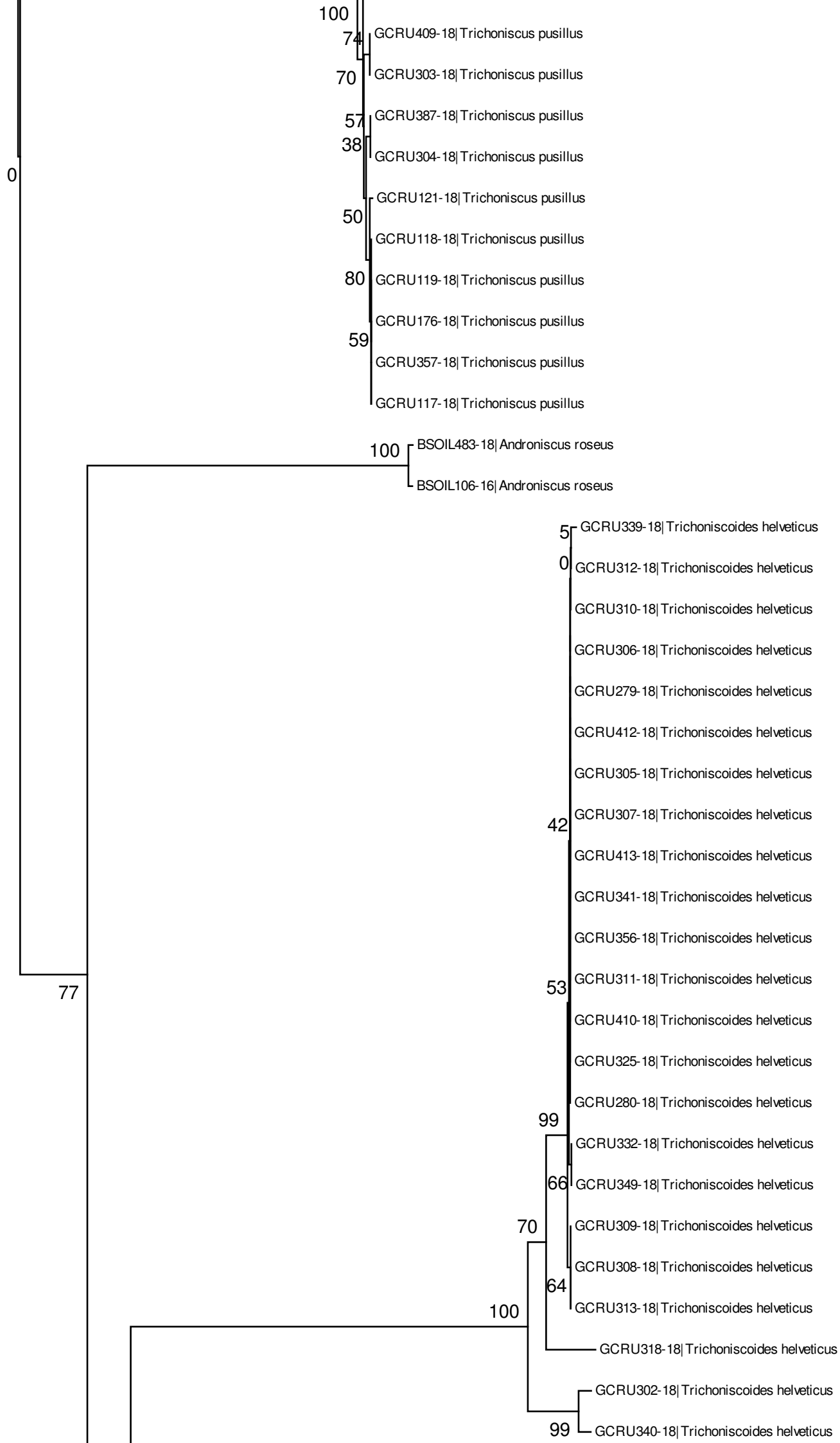

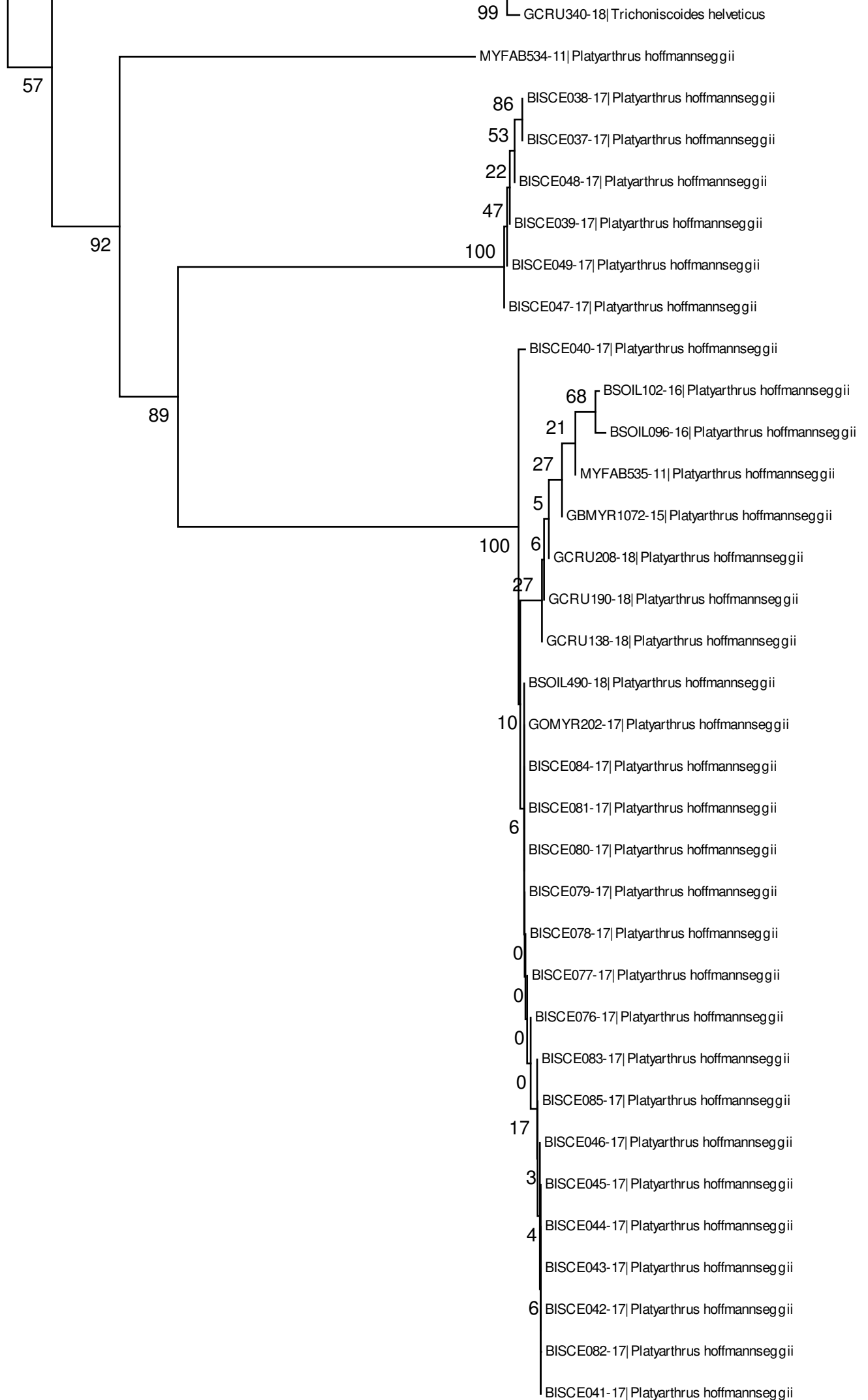

BISCE064-17|Ligia oceanica

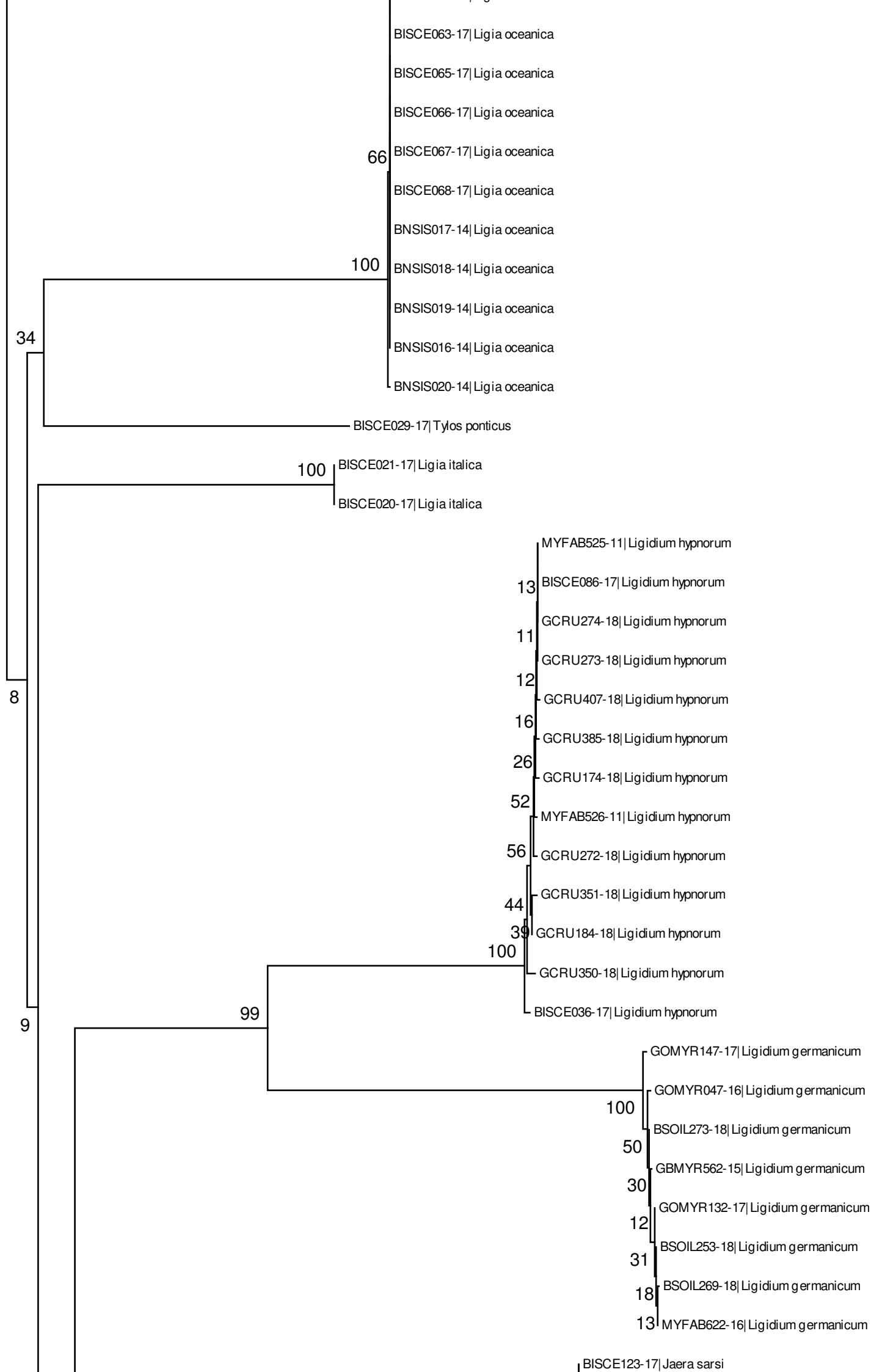

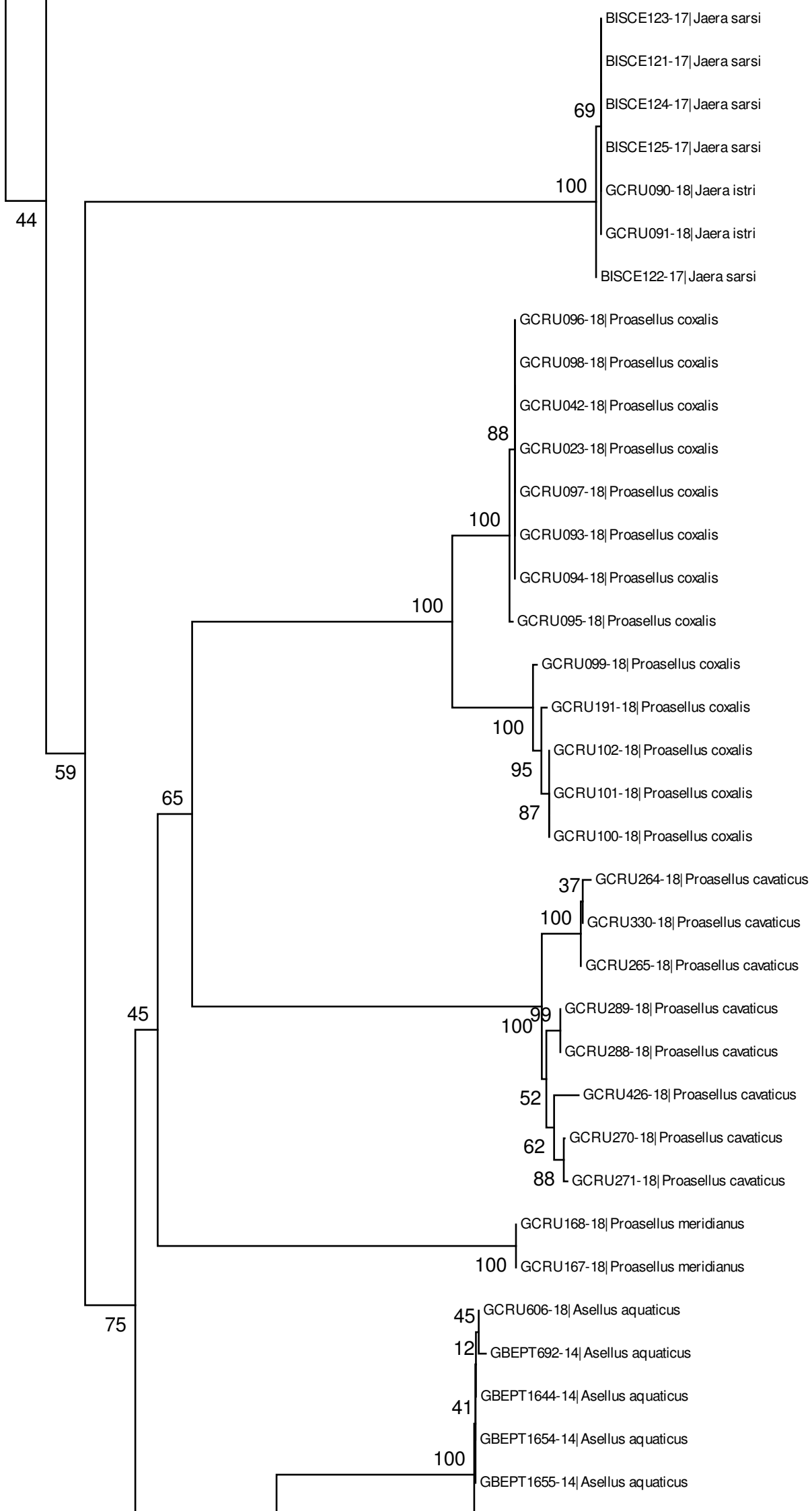

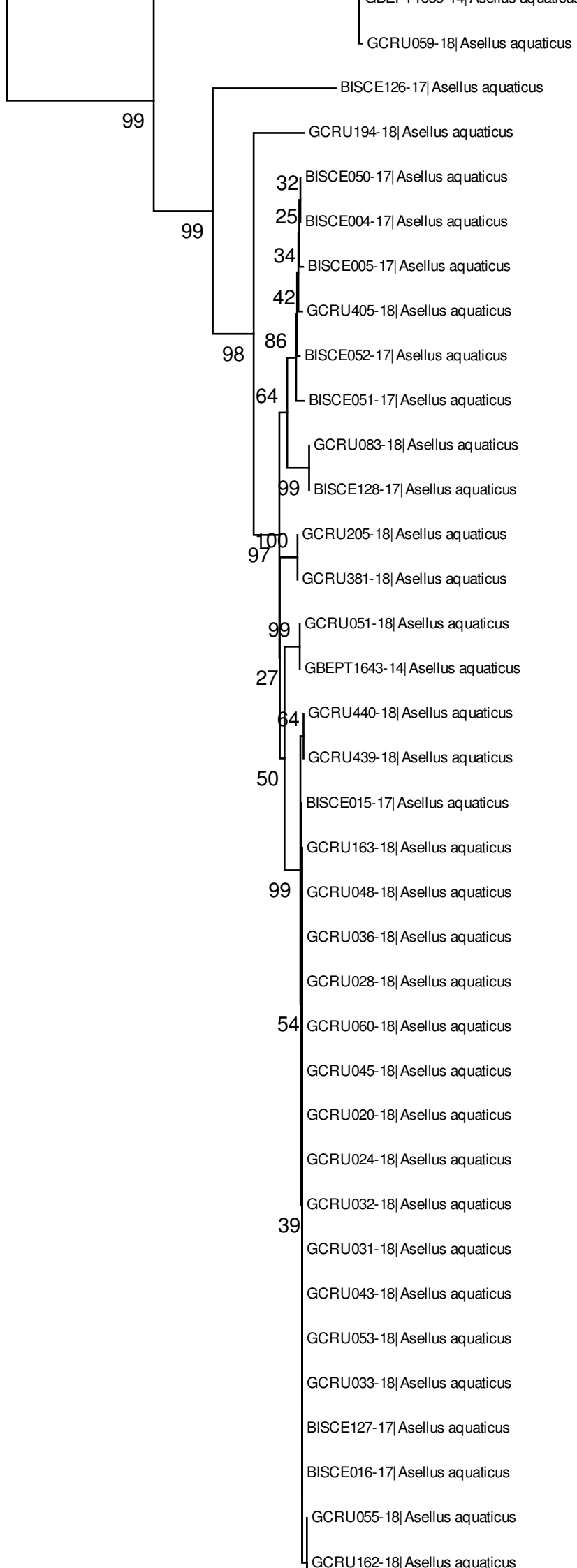

65 | GCRU162-18| *Asellus aquaticus*  
| GBEPT1641-14| *Asellus aquaticus*

0.050

Supplement: Supplementary material 2 — Neighbor-joining topology [file zookeys-1082-103-s002.pdf]
