# Supplementary material for: ﻿Surprisingly high genetic divergence of the mitochondrial DNA barcode fragment (COI) within Central European woodlice species (Crustacea, Isopoda, Oniscidea)
Source: Zookeys. 2022 Jan 20;1082:103–25. doi: 10.3897/zookeys.1082.69851 (PMC8794987; doi:10.3897/zookeys.1082.69851)
Supplement: Supplementary material 3 — Neighbor-joining topology of the BOLD workbench including BIN analysis [file zookeys-1082-103-s003.pdf]

BOLD TaxonID Tree

Title	:	Tree Result - DS-BISCE (518 records selected)
Date	:	07-Jun-2020
Data Type	:	Nucleotide
Distance Model	:	Kimura 2 Parameter
Marker	:	COI-5P
Colourization	:	Barcode Cluster (BIN)

Label	:	Process ID
Label	:	Taxon
Label	:	Country
Label	:	Province/State
Label	:	Region
Label	:	Sector
Label	:	Barcode Cluster (BIN)

Sequence Count	:	518
Species count	:	47
Genus count	:	23
Family count	:	13
Unidentified	:	0

BIN Count	:	76

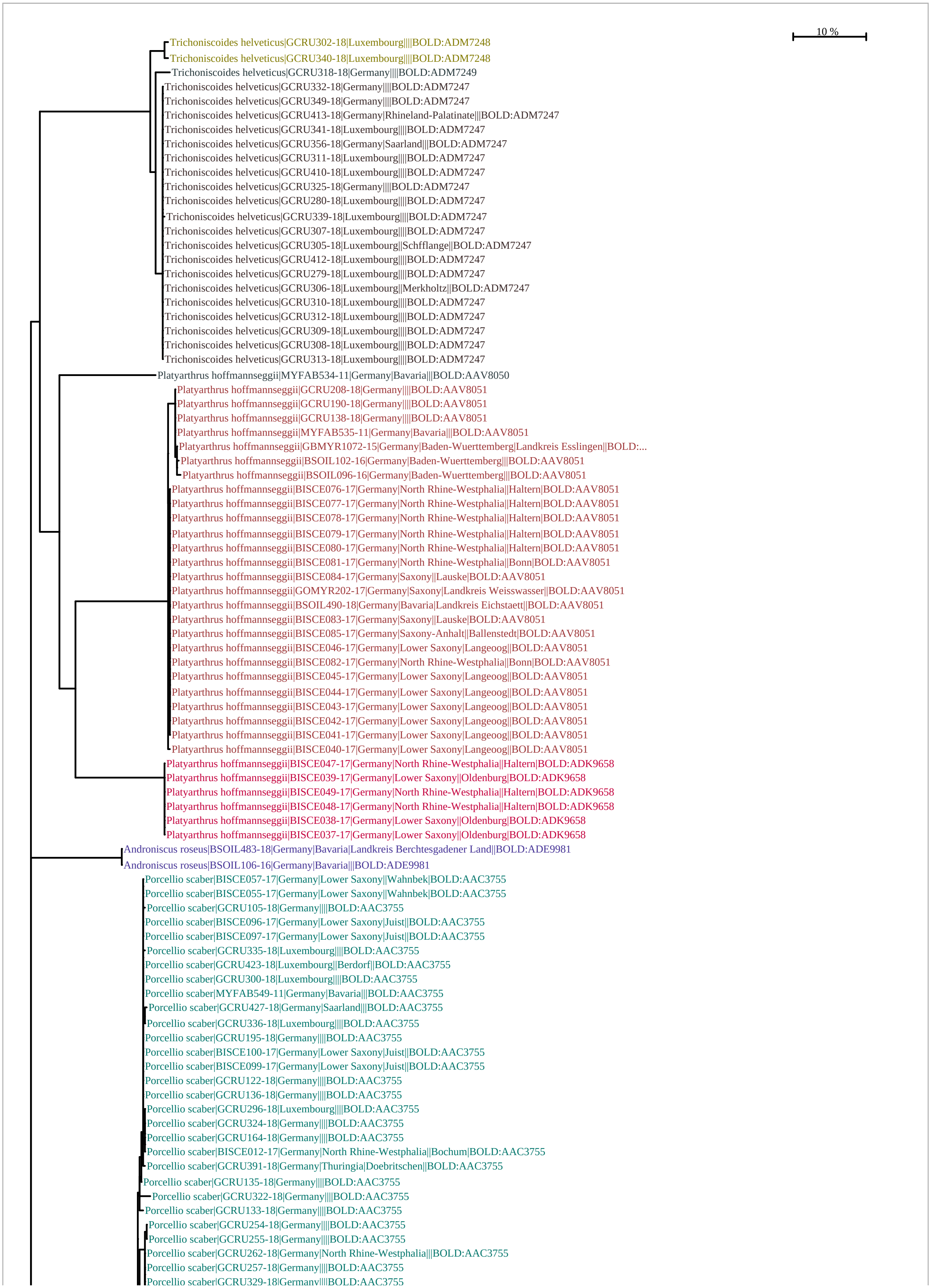
10 %
Trichoniscoides helveticus|GCRU302-18|Luxembourg||||BOLD:ADM7248
Trichoniscoides helveticus|GCRU340-18|Luxembourg||||BOLD:ADM7248
Trichoniscoides helveticus|GCRU318-18|Germany||||BOLD:ADM7249
Trichoniscoides helveticus|GCRU332-18|Germany||||BOLD:ADM7247
Trichoniscoides helveticus|GCRU349-18|Germany||||BOLD:ADM7247
Trichoniscoides helveticus|GCRU413-18|Germany|Rhineland-Palatinate|||BOLD:ADM7247
Trichoniscoides helveticus|GCRU341-18|Luxembourg||||BOLD:ADM7247
Trichoniscoides helveticus|GCRU356-18|Germany|Saarland|||BOLD:ADM7247
Trichoniscoides helveticus|GCRU311-18|Luxembourg||||BOLD:ADM7247
Trichoniscoides helveticus|GCRU410-18|Luxembourg||||BOLD:ADM7247
Trichoniscoides helveticus|GCRU325-18|Germany||||BOLD:ADM7247
Trichoniscoides helveticus|GCRU280-18|Luxembourg||||BOLD:ADM7247
Trichoniscoides helveticus|GCRU339-18|Luxembourg||||BOLD:ADM7247
Trichoniscoides helveticus|GCRU307-18|Luxembourg||||BOLD:ADM7247
Trichoniscoides helveticus|GCRU305-18|Luxembourg||Schfflange||BOLD:ADM7247
Trichoniscoides helveticus|GCRU412-18|Luxembourg||||BOLD:ADM7247
Trichoniscoides helveticus|GCRU279-18|Luxembourg||||BOLD:ADM7247
Trichoniscoides helveticus|GCRU306-18|Luxembourg||Merkholtz||BOLD:ADM7247
Trichoniscoides helveticus|GCRU310-18|Luxembourg||||BOLD:ADM7247
Trichoniscoides helveticus|GCRU312-18|Luxembourg||||BOLD:ADM7247
Trichoniscoides helveticus|GCRU309-18|Luxembourg||||BOLD:ADM7247
Trichoniscoides helveticus|GCRU308-18|Luxembourg||||BOLD:ADM7247
Trichoniscoides helveticus|GCRU313-18|Luxembourg||||BOLD:ADM7247
Platyarthrus hoffmannseggii|MYFAB534-11|Germany|Bavaria|||BOLD:AAV8050
Platyarthrus hoffmannseggii|GCRU208-18|Germany||||BOLD:AAV8051
Platyarthrus hoffmannseggii|GCRU190-18|Germany||||BOLD:AAV8051
Platyarthrus hoffmannseggii|GCRU138-18|Germany||||BOLD:AAV8051
Platyarthrus hoffmannseggii|MYFAB535-11|Germany|Bavaria|||BOLD:AAV8051
Platyarthrus hoffmannseggii|GBMYR1072-15|Germany|Baden-Wuerttemberg|Landkreis Esslingen||BOLD:...
Platyarthrus hoffmannseggii|BSOIL102-16|Germany|Baden-Wuerttemberg|||BOLD:AAV8051
Platyarthrus hoffmannseggii|BSOIL096-16|Germany|Baden-Wuerttemberg|||BOLD:AAV8051
Platyarthrus hoffmannseggii|BISCE076-17|Germany|North Rhine-Westphalia||Haltern|BOLD:AAV8051
Platyarthrus hoffmannseggii|BISCE077-17|Germany|North Rhine-Westphalia||Haltern|BOLD:AAV8051
Platyarthrus hoffmannseggii|BISCE078-17|Germany|North Rhine-Westphalia||Haltern|BOLD:AAV8051
Platyarthrus hoffmannseggii|BISCE079-17|Germany|North Rhine-Westphalia||Haltern|BOLD:AAV8051
Platyarthrus hoffmannseggii|BISCE080-17|Germany|North Rhine-Westphalia||Haltern|BOLD:AAV8051
Platyarthrus hoffmannseggii|BISCE081-17|Germany|North Rhine-Westphalia||Bonn|BOLD:AAV8051
Platyarthrus hoffmannseggii|BISCE084-17|Germany|Saxony||Lauske|BOLD:AAV8051
Platyarthrus hoffmannseggii|GOMYR202-17|Germany|Saxony|Landkreis Weisswasser||BOLD:AAV8051
Platyarthrus hoffmannseggii|BSOIL490-18|Germany|Bavaria|Landkreis Eichstaett||BOLD:AAV8051
Platyarthrus hoffmannseggii|BISCE083-17|Germany|Saxony||Lauske|BOLD:AAV8051
Platyarthrus hoffmannseggii|BISCE085-17|Germany|Saxony-Anhalt||Ballenstedt|BOLD:AAV8051
Platyarthrus hoffmannseggii|BISCE046-17|Germany|Lower Saxony|Langeoog||BOLD:AAV8051
Platyarthrus hoffmannseggii|BISCE082-17|Germany|North Rhine-Westphalia||Bonn|BOLD:AAV8051
Platyarthrus hoffmannseggii|BISCE045-17|Germany|Lower Saxony|Langeoog||BOLD:AAV8051
Platyarthrus hoffmannseggii|BISCE044-17|Germany|Lower Saxony|Langeoog||BOLD:AAV8051
Platyarthrus hoffmannseggii|BISCE043-17|Germany|Lower Saxony|Langeoog||BOLD:AAV8051
Platyarthrus hoffmannseggii|BISCE042-17|Germany|Lower Saxony|Langeoog||BOLD:AAV8051
Platyarthrus hoffmannseggii|BISCE041-17|Germany|Lower Saxony|Langeoog||BOLD:AAV8051
Platyarthrus hoffmannseggii|BISCE040-17|Germany|Lower Saxony|Langeoog||BOLD:AAV8051
Platyarthrus hoffmannseggii|BISCE047-17|Germany|North Rhine-Westphalia||Haltern|BOLD:ADK9658
Platyarthrus hoffmannseggii|BISCE039-17|Germany|Lower Saxony||Oldenburg|BOLD:ADK9658
Platyarthrus hoffmannseggii|BISCE049-17|Germany|North Rhine-Westphalia||Haltern|BOLD:ADK9658
Platyarthrus hoffmannseggii|BISCE048-17|Germany|North Rhine-Westphalia||Haltern|BOLD:ADK9658
Platyarthrus hoffmannseggii|BISCE038-17|Germany|Lower Saxony||Oldenburg|BOLD:ADK9658
Platyarthrus hoffmannseggii|BISCE037-17|Germany|Lower Saxony||Oldenburg|BOLD:ADK9658
Androniscus roseus|BSOIL483-18|Germany|Bavaria|Landkreis Berchtesgadener Land||BOLD:ADE9981
Androniscus roseus|BSOIL106-16|Germany|Bavaria|||BOLD:ADE9981
Porcellio scaber|BISCE057-17|Germany|Lower Saxony||Wahnbek|BOLD:AAC3755
Porcellio scaber|BISCE055-17|Germany|Lower Saxony||Wahnbek|BOLD:AAC3755
Porcellio scaber|GCRU105-18|Germany||||BOLD:AAC3755
Porcellio scaber|BISCE096-17|Germany|Lower Saxony|Juist||BOLD:AAC3755
Porcellio scaber|BISCE097-17|Germany|Lower Saxony|Juist||BOLD:AAC3755
Porcellio scaber|GCRU335-18|Luxembourg||||BOLD:AAC3755
Porcellio scaber|GCRU423-18|Luxembourg||Berdorf||BOLD:AAC3755
Porcellio scaber|GCRU300-18|Luxembourg||||BOLD:AAC3755
Porcellio scaber|MYFAB549-11|Germany|Bavaria|||BOLD:AAC3755
Porcellio scaber|GCRU427-18|Germany|Saarland|||BOLD:AAC3755
Porcellio scaber|GCRU336-18|Luxembourg||||BOLD:AAC3755
Porcellio scaber|GCRU195-18|Germany||||BOLD:AAC3755
Porcellio scaber|BISCE100-17|Germany|Lower Saxony|Juist||BOLD:AAC3755
Porcellio scaber|BISCE099-17|Germany|Lower Saxony|Juist||BOLD:AAC3755
Porcellio scaber|GCRU122-18|Germany||||BOLD:AAC3755
Porcellio scaber|GCRU136-18|Germany||||BOLD:AAC3755
Porcellio scaber|GCRU296-18|Luxembourg||||BOLD:AAC3755
Porcellio scaber|GCRU324-18|Germany||||BOLD:AAC3755
Porcellio scaber|GCRU164-18|Germany||||BOLD:AAC3755
Porcellio scaber|BISCE012-17|Germany|North Rhine-Westphalia||Bochum|BOLD:AAC3755
Porcellio scaber|GCRU391-18|Germany|Thuringia|Doebritschen||BOLD:AAC3755
Porcellio scaber|GCRU135-18|Germany||||BOLD:AAC3755
Porcellio scaber|GCRU322-18|Germany||||BOLD:AAC3755
Porcellio scaber|GCRU133-18|Germany||||BOLD:AAC3755
Porcellio scaber|GCRU254-18|Germany||||BOLD:AAC3755
Porcellio scaber|GCRU255-18|Germany||||BOLD:AAC3755
Porcellio scaber|GCRU262-18|Germany|North Rhine-Westphalia|||BOLD:AAC3755
Porcellio scaber|GCRU257-18|Germany||||BOLD:AAC3755
Porcellio scaber|GCRU329-18|Germany||||BOLD:AAC3755

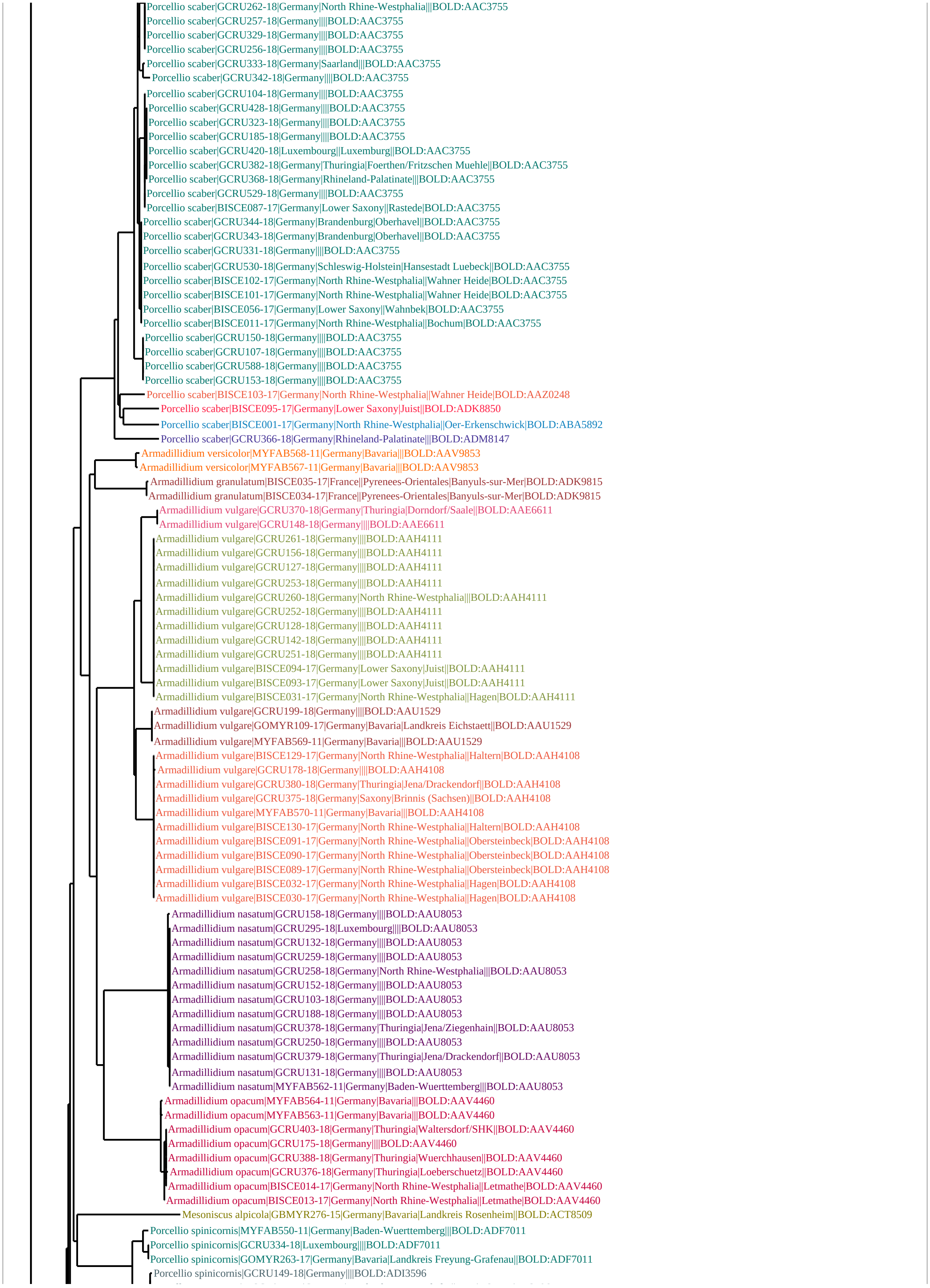
Porcellio scaber|GCRU262-18|Germany|North Rhine-Westphalia|||BOLD:AAC3755
Porcellio scaber|GCRU257-18|Germany||||BOLD:AAC3755
Porcellio scaber|GCRU329-18|Germany||||BOLD:AAC3755
Porcellio scaber|GCRU256-18|Germany||||BOLD:AAC3755
Porcellio scaber|GCRU333-18|Germany|Saarland|||BOLD:AAC3755
Porcellio scaber|GCRU342-18|Germany||||BOLD:AAC3755
Porcellio scaber|GCRU104-18|Germany||||BOLD:AAC3755
Porcellio scaber|GCRU428-18|Germany||||BOLD:AAC3755
Porcellio scaber|GCRU323-18|Germany||||BOLD:AAC3755
Porcellio scaber|GCRU185-18|Germany||||BOLD:AAC3755
Porcellio scaber|GCRU420-18|Luxembourg||Luxemburg||BOLD:AAC3755
Porcellio scaber|GCRU382-18|Germany|Thuringia|Foerthen/Fritzschen Muehle||BOLD:AAC3755
Porcellio scaber|GCRU368-18|Germany|Rhineland-Palatinate|||BOLD:AAC3755
Porcellio scaber|GCRU529-18|Germany||||BOLD:AAC3755
Porcellio scaber|BISCE087-17|Germany|Lower Saxony||Rastede|BOLD:AAC3755
Porcellio scaber|GCRU344-18|Germany|Brandenburg|Oberhavel||BOLD:AAC3755
Porcellio scaber|GCRU343-18|Germany|Brandenburg|Oberhavel||BOLD:AAC3755
Porcellio scaber|GCRU331-18|Germany||||BOLD:AAC3755
Porcellio scaber|GCRU530-18|Germany|Schleswig-Holstein|Hansestadt Luebeck||BOLD:AAC3755
Porcellio scaber|BISCE102-17|Germany|North Rhine-Westphalia||Wahner Heide|BOLD:AAC3755
Porcellio scaber|BISCE101-17|Germany|North Rhine-Westphalia||Wahner Heide|BOLD:AAC3755
Porcellio scaber|BISCE056-17|Germany|Lower Saxony||Wahnbek|BOLD:AAC3755
Porcellio scaber|BISCE011-17|Germany|North Rhine-Westphalia||Bochum|BOLD:AAC3755
Porcellio scaber|GCRU150-18|Germany||||BOLD:AAC3755
Porcellio scaber|GCRU107-18|Germany||||BOLD:AAC3755
Porcellio scaber|GCRU588-18|Germany||||BOLD:AAC3755
Porcellio scaber|GCRU153-18|Germany||||BOLD:AAC3755
Porcellio scaber|BISCE103-17|Germany|North Rhine-Westphalia||Wahner Heide|BOLD:AAZ0248
Porcellio scaber|BISCE095-17|Germany|Lower Saxony|Juist||BOLD:ADK8850
Porcellio scaber|BISCE001-17|Germany|North Rhine-Westphalia||Oer-Erkenschwick|BOLD:ABA5892
Porcellio scaber|GCRU366-18|Germany|Rhineland-Palatinate|||BOLD:ADM8147
Armadillidium versicolor|MYFAB568-11|Germany|Bavaria|||BOLD:AAV9853
Armadillidium versicolor|MYFAB567-11|Germany|Bavaria|||BOLD:AAV9853
Armadillidium granulatum|BISCE035-17|France||Pyrenees-Orientales|Banyuls-sur-Mer|BOLD:ADK9815
Armadillidium granulatum|BISCE034-17|France||Pyrenees-Orientales|Banyuls-sur-Mer|BOLD:ADK9815
Armadillidium vulgare|GCRU370-18|Germany|Thuringia|Dorndorf/Saale||BOLD:AAE6611
Armadillidium vulgare|GCRU148-18|Germany||||BOLD:AAE6611
Armadillidium vulgare|GCRU261-18|Germany||||BOLD:AAH4111
Armadillidium vulgare|GCRU156-18|Germany||||BOLD:AAH4111
Armadillidium vulgare|GCRU127-18|Germany||||BOLD:AAH4111
Armadillidium vulgare|GCRU253-18|Germany||||BOLD:AAH4111
Armadillidium vulgare|GCRU260-18|Germany|North Rhine-Westphalia|||BOLD:AAH4111
Armadillidium vulgare|GCRU252-18|Germany||||BOLD:AAH4111
Armadillidium vulgare|GCRU128-18|Germany||||BOLD:AAH4111
Armadillidium vulgare|GCRU142-18|Germany||||BOLD:AAH4111
Armadillidium vulgare|GCRU251-18|Germany||||BOLD:AAH4111
Armadillidium vulgare|BISCE094-17|Germany|Lower Saxony|Juist||BOLD:AAH4111
Armadillidium vulgare|BISCE093-17|Germany|Lower Saxony|Juist||BOLD:AAH4111
Armadillidium vulgare|BISCE031-17|Germany|North Rhine-Westphalia||Hagen|BOLD:AAH4111
Armadillidium vulgare|GCRU199-18|Germany||||BOLD:AAU1529
Armadillidium vulgare|GOMYR109-17|Germany|Bavaria|Landkreis Eichstaett||BOLD:AAU1529
Armadillidium vulgare|MYFAB569-11|Germany|Bavaria|||BOLD:AAU1529
Armadillidium vulgare|BISCE129-17|Germany|North Rhine-Westphalia||Haltern|BOLD:AAH4108
Armadillidium vulgare|GCRU178-18|Germany||||BOLD:AAH4108
Armadillidium vulgare|GCRU380-18|Germany|Thuringia|Jena/Drackendorf||BOLD:AAH4108
Armadillidium vulgare|GCRU375-18|Germany|Saxony|Brinnis (Sachsen)||BOLD:AAH4108
Armadillidium vulgare|MYFAB570-11|Germany|Bavaria|||BOLD:AAH4108
Armadillidium vulgare|BISCE130-17|Germany|North Rhine-Westphalia||Haltern|BOLD:AAH4108
Armadillidium vulgare|BISCE091-17|Germany|North Rhine-Westphalia||Obersteinbeck|BOLD:AAH4108
Armadillidium vulgare|BISCE090-17|Germany|North Rhine-Westphalia||Obersteinbeck|BOLD:AAH4108
Armadillidium vulgare|BISCE089-17|Germany|North Rhine-Westphalia||Obersteinbeck|BOLD:AAH4108
Armadillidium vulgare|BISCE032-17|Germany|North Rhine-Westphalia||Hagen|BOLD:AAH4108
Armadillidium vulgare|BISCE030-17|Germany|North Rhine-Westphalia||Hagen|BOLD:AAH4108
Armadillidium nasatum|GCRU158-18|Germany||||BOLD:AAU8053
Armadillidium nasatum|GCRU295-18|Luxembourg||||BOLD:AAU8053
Armadillidium nasatum|GCRU132-18|Germany||||BOLD:AAU8053
Armadillidium nasatum|GCRU259-18|Germany||||BOLD:AAU8053
Armadillidium nasatum|GCRU258-18|Germany|North Rhine-Westphalia|||BOLD:AAU8053
Armadillidium nasatum|GCRU152-18|Germany||||BOLD:AAU8053
Armadillidium nasatum|GCRU103-18|Germany||||BOLD:AAU8053
Armadillidium nasatum|GCRU188-18|Germany||||BOLD:AAU8053
Armadillidium nasatum|GCRU378-18|Germany|Thuringia|Jena/Ziegenhain||BOLD:AAU8053
Armadillidium nasatum|GCRU250-18|Germany||||BOLD:AAU8053
Armadillidium nasatum|GCRU379-18|Germany|Thuringia|Jena/Drackendorf||BOLD:AAU8053
Armadillidium nasatum|GCRU131-18|Germany||||BOLD:AAU8053
Armadillidium nasatum|MYFAB562-11|Germany|Baden-Wuerttemberg|||BOLD:AAU8053
Armadillidium opacum|MYFAB564-11|Germany|Bavaria|||BOLD:AAV4460
Armadillidium opacum|MYFAB563-11|Germany|Bavaria|||BOLD:AAV4460
Armadillidium opacum|GCRU403-18|Germany|Thuringia|Waltersdorf/SHK||BOLD:AAV4460
Armadillidium opacum|GCRU175-18|Germany||||BOLD:AAV4460
Armadillidium opacum|GCRU388-18|Germany|Thuringia|Wuerchhausen||BOLD:AAV4460
Armadillidium opacum|GCRU376-18|Germany|Thuringia|Loeberschuetz||BOLD:AAV4460
Armadillidium opacum|BISCE014-17|Germany|North Rhine-Westphalia||Letmathe|BOLD:AAV4460
Armadillidium opacum|BISCE013-17|Germany|North Rhine-Westphalia||Letmathe|BOLD:AAV4460
Mesoniscus alpicola|GBMYR276-15|Germany|Bavaria|Landkreis Rosenheim||BOLD:ACT8509
Porcellio spinicornis|MYFAB550-11|Germany|Baden-Wuerttemberg|||BOLD:ADF7011
Porcellio spinicornis|GCRU334-18|Luxembourg||||BOLD:ADF7011
Porcellio spinicornis|GOMYR263-17|Germany|Bavaria|Landkreis Freyung-Grafenau||BOLD:ADF7011
Porcellio spinicornis|GCRU149-18|Germany||||BOLD:ADI3596

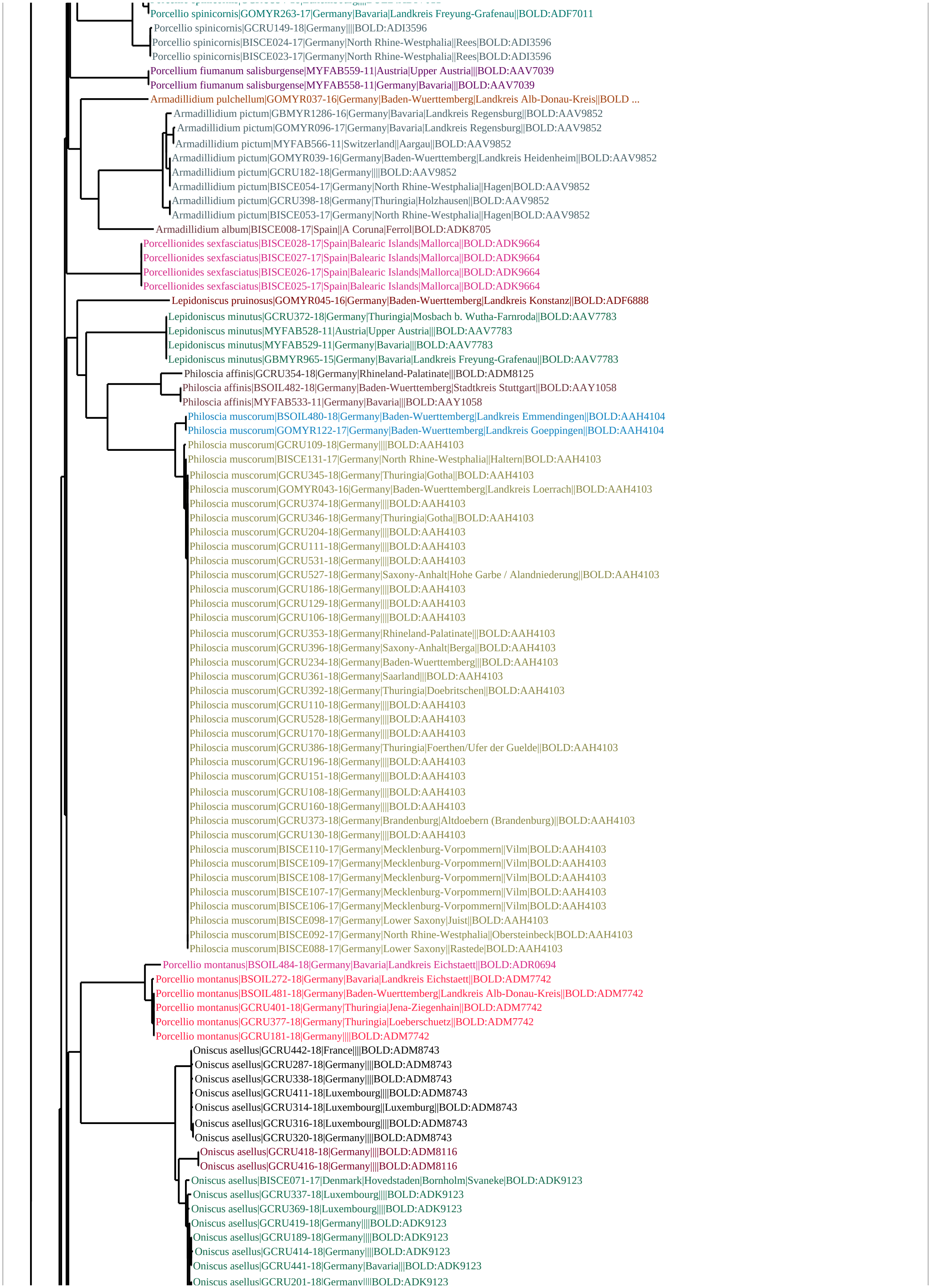
Porcellio spinicornis|GOMYR263-17|Germany|Bavaria|Landkreis Freyung-Grafenau||BOLD:ADF7011
Porcellio spinicornis|GCRU149-18|Germany||||BOLD:ADI3596
Porcellio spinicornis|BISCE024-17|Germany|North Rhine-Westphalia||Rees|BOLD:ADI3596
Porcellio spinicornis|BISCE023-17|Germany|North Rhine-Westphalia||Rees|BOLD:ADI3596
Porcellium fiumanum salisburgense|MYFAB559-11|Austria|Upper Austria|||BOLD:AAV7039
Porcellium fiumanum salisburgense|MYFAB558-11|Germany|Bavaria|||BOLD:AAV7039
Armadillidium pulchellum|GOMYR037-16|Germany|Baden-Wuerttemberg|Landkreis Alb-Donau-Kreis||BOLD ...
Armadillidium pictum|GBMYR1286-16|Germany|Bavaria|Landkreis Regensburg||BOLD:AAV9852
Armadillidium pictum|GOMYR096-17|Germany|Bavaria|Landkreis Regensburg||BOLD:AAV9852
Armadillidium pictum|MYFAB566-11|Switzerland||Aargau||BOLD:AAV9852
Armadillidium pictum|GOMYR039-16|Germany|Baden-Wuerttemberg|Landkreis Heidenheim||BOLD:AAV9852
Armadillidium pictum|GCRU182-18|Germany||||BOLD:AAV9852
Armadillidium pictum|BISCE054-17|Germany|North Rhine-Westphalia||Hagen|BOLD:AAV9852
Armadillidium pictum|GCRU398-18|Germany|Thuringia|Holzhausen||BOLD:AAV9852
Armadillidium pictum|BISCE053-17|Germany|North Rhine-Westphalia||Hagen|BOLD:AAV9852
Armadillidium album|BISCE008-17|Spain||A Coruna|Ferrol|BOLD:ADK8705
Porcellionides sexfasciatus|BISCE028-17|Spain|Balearic Islands|Mallorca||BOLD:ADK9664
Porcellionides sexfasciatus|BISCE027-17|Spain|Balearic Islands|Mallorca||BOLD:ADK9664
Porcellionides sexfasciatus|BISCE026-17|Spain|Balearic Islands|Mallorca||BOLD:ADK9664
Porcellionides sexfasciatus|BISCE025-17|Spain|Balearic Islands|Mallorca||BOLD:ADK9664
Lepidoniscus pruinosus|GOMYR045-16|Germany|Baden-Wuerttemberg|Landkreis Konstanz||BOLD:ADF6888
Lepidoniscus minutus|GCRU372-18|Germany|Thuringia|Mosbach b. Wutha-Farnroda||BOLD:AAV7783
Lepidoniscus minutus|MYFAB528-11|Austria|Upper Austria|||BOLD:AAV7783
Lepidoniscus minutus|MYFAB529-11|Germany|Bavaria|||BOLD:AAV7783
Lepidoniscus minutus|GBMYR965-15|Germany|Bavaria|Landkreis Freyung-Grafenau||BOLD:AAV7783
Philoscia affinis|GCRU354-18|Germany|Rhineland-Palatinate|||BOLD:ADM8125
Philoscia affinis|BSOIL482-18|Germany|Baden-Wuerttemberg|Stadtkreis Stuttgart||BOLD:AAY1058
Philoscia affinis|MYFAB533-11|Germany|Bavaria|||BOLD:AAY1058
Philoscia muscorum|BSOIL480-18|Germany|Baden-Wuerttemberg|Landkreis Emmendingen||BOLD:AAH4104
Philoscia muscorum|GOMYR122-17|Germany|Baden-Wuerttemberg|Landkreis Goeppingen||BOLD:AAH4104
Philoscia muscorum|GCRU109-18|Germany||||BOLD:AAH4103
Philoscia muscorum|BISCE131-17|Germany|North Rhine-Westphalia||Haltern|BOLD:AAH4103
Philoscia muscorum|GCRU345-18|Germany|Thuringia|Gotha||BOLD:AAH4103
Philoscia muscorum|GOMYR043-16|Germany|Baden-Wuerttemberg|Landkreis Loerrach||BOLD:AAH4103
Philoscia muscorum|GCRU374-18|Germany||||BOLD:AAH4103
Philoscia muscorum|GCRU346-18|Germany|Thuringia|Gotha||BOLD:AAH4103
Philoscia muscorum|GCRU204-18|Germany||||BOLD:AAH4103
Philoscia muscorum|GCRU111-18|Germany||||BOLD:AAH4103
Philoscia muscorum|GCRU531-18|Germany||||BOLD:AAH4103
Philoscia muscorum|GCRU527-18|Germany|Saxony-Anhalt|Hohe Garbe / Alandniederung||BOLD:AAH4103
Philoscia muscorum|GCRU186-18|Germany||||BOLD:AAH4103
Philoscia muscorum|GCRU129-18|Germany||||BOLD:AAH4103
Philoscia muscorum|GCRU106-18|Germany||||BOLD:AAH4103
Philoscia muscorum|GCRU353-18|Germany|Rhineland-Palatinate|||BOLD:AAH4103
Philoscia muscorum|GCRU396-18|Germany|Saxony-Anhalt|Berga||BOLD:AAH4103
Philoscia muscorum|GCRU234-18|Germany|Baden-Wuerttemberg|||BOLD:AAH4103
Philoscia muscorum|GCRU361-18|Germany|Saarland|||BOLD:AAH4103
Philoscia muscorum|GCRU392-18|Germany|Thuringia|Doebritschen||BOLD:AAH4103
Philoscia muscorum|GCRU110-18|Germany||||BOLD:AAH4103
Philoscia muscorum|GCRU528-18|Germany||||BOLD:AAH4103
Philoscia muscorum|GCRU170-18|Germany||||BOLD:AAH4103
Philoscia muscorum|GCRU386-18|Germany|Thuringia|Foerthen/Ufer der Guelde||BOLD:AAH4103
Philoscia muscorum|GCRU196-18|Germany||||BOLD:AAH4103
Philoscia muscorum|GCRU151-18|Germany||||BOLD:AAH4103
Philoscia muscorum|GCRU108-18|Germany||||BOLD:AAH4103
Philoscia muscorum|GCRU160-18|Germany||||BOLD:AAH4103
Philoscia muscorum|GCRU373-18|Germany|Brandenburg|Altdoebern (Brandenburg)||BOLD:AAH4103
Philoscia muscorum|GCRU130-18|Germany||||BOLD:AAH4103
Philoscia muscorum|BISCE110-17|Germany|Mecklenburg-Vorpommern||Vilm|BOLD:AAH4103
Philoscia muscorum|BISCE109-17|Germany|Mecklenburg-Vorpommern||Vilm|BOLD:AAH4103
Philoscia muscorum|BISCE108-17|Germany|Mecklenburg-Vorpommern||Vilm|BOLD:AAH4103
Philoscia muscorum|BISCE107-17|Germany|Mecklenburg-Vorpommern||Vilm|BOLD:AAH4103
Philoscia muscorum|BISCE106-17|Germany|Mecklenburg-Vorpommern||Vilm|BOLD:AAH4103
Philoscia muscorum|BISCE098-17|Germany|Lower Saxony|Juist||BOLD:AAH4103
Philoscia muscorum|BISCE092-17|Germany|North Rhine-Westphalia||Obersteinbeck|BOLD:AAH4103
Philoscia muscorum|BISCE088-17|Germany|Lower Saxony||Rastede|BOLD:AAH4103
Porcellio montanus|BSOIL484-18|Germany|Bavaria|Landkreis Eichstaett||BOLD:ADR0694
Porcellio montanus|BSOIL272-18|Germany|Bavaria|Landkreis Eichstaett||BOLD:ADM7742
Porcellio montanus|BSOIL481-18|Germany|Baden-Wuerttemberg|Landkreis Alb-Donau-Kreis||BOLD:ADM7742
Porcellio montanus|GCRU401-18|Germany|Thuringia|Jena-Ziegenhain||BOLD:ADM7742
Porcellio montanus|GCRU377-18|Germany|Thuringia|Loeberschuetz||BOLD:ADM7742
Porcellio montanus|GCRU181-18|Germany||||BOLD:ADM7742
Oniscus asellus|GCRU442-18|France||||BOLD:ADM8743
Oniscus asellus|GCRU287-18|Germany||||BOLD:ADM8743
Oniscus asellus|GCRU338-18|Germany||||BOLD:ADM8743
Oniscus asellus|GCRU411-18|Luxembourg||||BOLD:ADM8743
Oniscus asellus|GCRU314-18|Luxembourg||Luxembourg||BOLD:ADM8743
Oniscus asellus|GCRU316-18|Luxembourg||||BOLD:ADM8743
Oniscus asellus|GCRU320-18|Germany||||BOLD:ADM8743
Oniscus asellus|GCRU418-18|Germany||||BOLD:ADM8116
Oniscus asellus|GCRU416-18|Germany||||BOLD:ADM8116
Oniscus asellus|BISCE071-17|Denmark|Hovedstaden|Bornholm|Svaneke|BOLD:ADK9123
Oniscus asellus|GCRU337-18|Luxembourg||||BOLD:ADK9123
Oniscus asellus|GCRU369-18|Luxembourg||||BOLD:ADK9123
Oniscus asellus|GCRU419-18|Germany||||BOLD:ADK9123
Oniscus asellus|GCRU189-18|Germany||||BOLD:ADK9123
Oniscus asellus|GCRU414-18|Germany||||BOLD:ADK9123
Oniscus asellus|GCRU441-18|Germany|Bavaria|||BOLD:ADK9123
Oniscus asellus|GCRU201-18|Germany||||BOLD:ADK9123

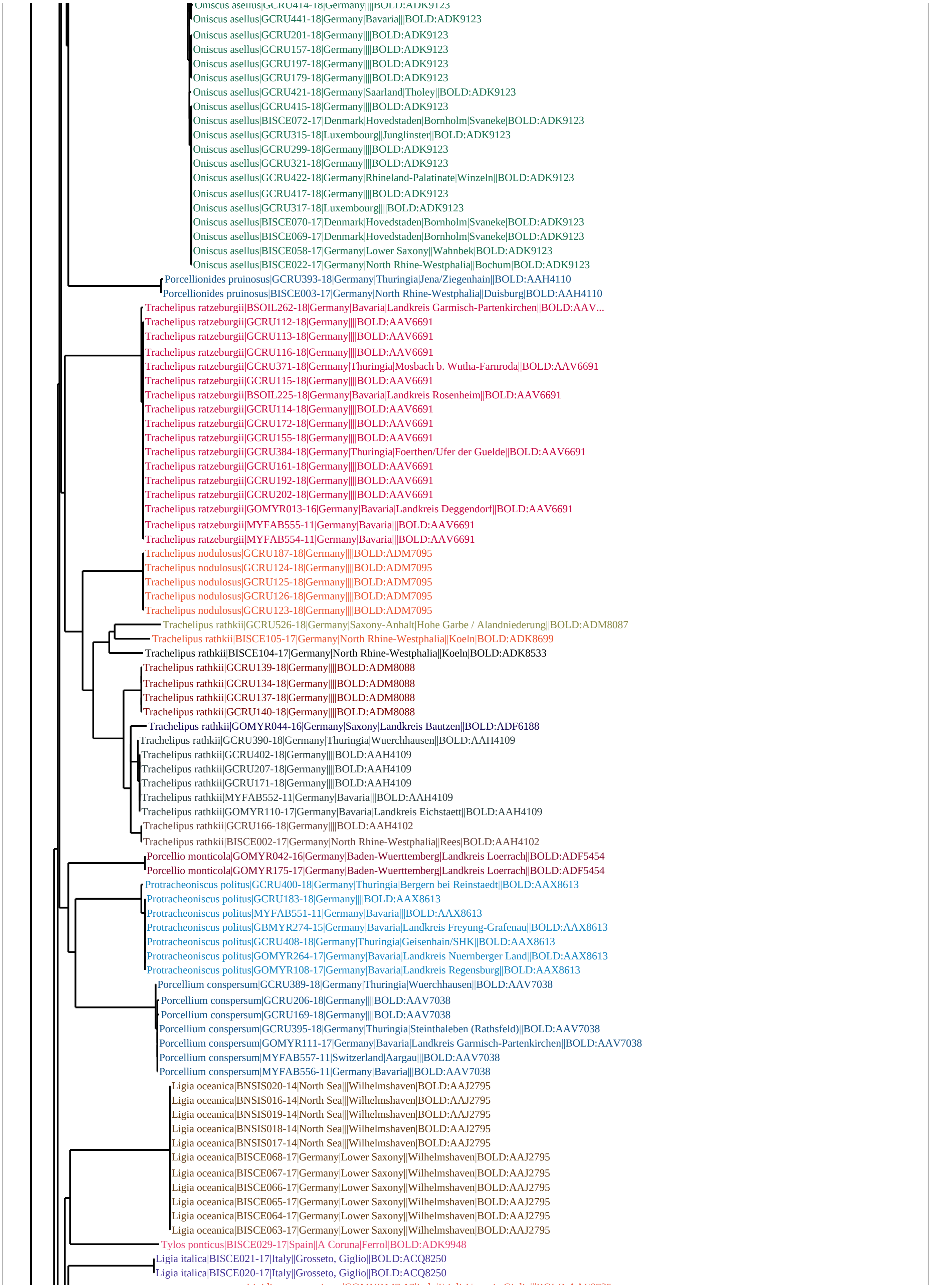

Oniscus asellus|GCRU414-18|Germany||||BOLD:ADK9123
Oniscus asellus|GCRU441-18|Germany|Bavaria|||BOLD:ADK9123
Oniscus asellus|GCRU201-18|Germany||||BOLD:ADK9123
Oniscus asellus|GCRU157-18|Germany||||BOLD:ADK9123
Oniscus asellus|GCRU197-18|Germany||||BOLD:ADK9123
Oniscus asellus|GCRU179-18|Germany||||BOLD:ADK9123
Oniscus asellus|GCRU421-18|Germany|Saarland|Tholey||BOLD:ADK9123
Oniscus asellus|GCRU415-18|Germany||||BOLD:ADK9123
Oniscus asellus|BISCE072-17|Denmark|Hovedstaden|Bornholm|Svaneke|BOLD:ADK9123
Oniscus asellus|GCRU315-18|Luxembourg||Junglinster||BOLD:ADK9123
Oniscus asellus|GCRU299-18|Germany||||BOLD:ADK9123
Oniscus asellus|GCRU321-18|Germany||||BOLD:ADK9123
Oniscus asellus|GCRU422-18|Germany|Rhineland-Palatinate|Winzeln||BOLD:ADK9123
Oniscus asellus|GCRU417-18|Germany||||BOLD:ADK9123
Oniscus asellus|GCRU317-18|Luxembourg||||BOLD:ADK9123
Oniscus asellus|BISCE070-17|Denmark|Hovedstaden|Bornholm|Svaneke|BOLD:ADK9123
Oniscus asellus|BISCE069-17|Denmark|Hovedstaden|Bornholm|Svaneke|BOLD:ADK9123
Oniscus asellus|BISCE058-17|Germany|Lower Saxony||Wahnbek|BOLD:ADK9123
Oniscus asellus|BISCE022-17|Germany|North Rhine-Westphalia||Bochum|BOLD:ADK9123
Porcellionides pruinosus|GCRU393-18|Germany|Thuringia|Jena/Ziegenhain||BOLD:AAH4110
Porcellionides pruinosus|BISCE003-17|Germany|North Rhine-Westphalia||Duisburg|BOLD:AAH4110
Trachelipus ratzeburgii|BSOIL262-18|Germany|Bavaria|Landkreis Garmisch-Partenkirchen||BOLD:AAV...
Trachelipus ratzeburgii|GCRU112-18|Germany||||BOLD:AAV6691
Trachelipus ratzeburgii|GCRU113-18|Germany||||BOLD:AAV6691
Trachelipus ratzeburgii|GCRU116-18|Germany||||BOLD:AAV6691
Trachelipus ratzeburgii|GCRU371-18|Germany|Thuringia|Mosbach b. Wutha-Farnroda||BOLD:AAV6691
Trachelipus ratzeburgii|GCRU115-18|Germany||||BOLD:AAV6691
Trachelipus ratzeburgii|BSOIL225-18|Germany|Bavaria|Landkreis Rosenheim||BOLD:AAV6691
Trachelipus ratzeburgii|GCRU114-18|Germany||||BOLD:AAV6691
Trachelipus ratzeburgii|GCRU172-18|Germany||||BOLD:AAV6691
Trachelipus ratzeburgii|GCRU155-18|Germany||||BOLD:AAV6691
Trachelipus ratzeburgii|GCRU384-18|Germany|Thuringia|Foerthen/Ufer der Guelde||BOLD:AAV6691
Trachelipus ratzeburgii|GCRU161-18|Germany||||BOLD:AAV6691
Trachelipus ratzeburgii|GCRU192-18|Germany||||BOLD:AAV6691
Trachelipus ratzeburgii|GCRU202-18|Germany||||BOLD:AAV6691
Trachelipus ratzeburgii|GOMYR013-16|Germany|Bavaria|Landkreis Deggendorf||BOLD:AAV6691
Trachelipus ratzeburgii|MYFAB555-11|Germany|Bavaria|||BOLD:AAV6691
Trachelipus ratzeburgii|MYFAB554-11|Germany|Bavaria|||BOLD:AAV6691
Trachelipus nodulosus|GCRU187-18|Germany||||BOLD:ADM7095
Trachelipus nodulosus|GCRU124-18|Germany||||BOLD:ADM7095
Trachelipus nodulosus|GCRU125-18|Germany||||BOLD:ADM7095
Trachelipus nodulosus|GCRU126-18|Germany||||BOLD:ADM7095
Trachelipus nodulosus|GCRU123-18|Germany||||BOLD:ADM7095
Trachelipus rathkii|GCRU526-18|Germany|Saxony-Anhalt|Hohe Garbe / Alandniederung||BOLD:ADM8087
Trachelipus rathkii|BISCE105-17|Germany|North Rhine-Westphalia||Koeln|BOLD:ADK8699
Trachelipus rathkii|BISCE104-17|Germany|North Rhine-Westphalia||Koeln|BOLD:ADK8533
Trachelipus rathkii|GCRU139-18|Germany||||BOLD:ADM8088
Trachelipus rathkii|GCRU134-18|Germany||||BOLD:ADM8088
Trachelipus rathkii|GCRU137-18|Germany||||BOLD:ADM8088
Trachelipus rathkii|GCRU140-18|Germany||||BOLD:ADM8088
Trachelipus rathkii|GOMYR044-16|Germany|Saxony|Landkreis Bautzen||BOLD:ADF6188
Trachelipus rathkii|GCRU390-18|Germany|Thuringia|Wuerchhausen||BOLD:AAH4109
Trachelipus rathkii|GCRU402-18|Germany||||BOLD:AAH4109
Trachelipus rathkii|GCRU207-18|Germany||||BOLD:AAH4109
Trachelipus rathkii|GCRU171-18|Germany||||BOLD:AAH4109
Trachelipus rathkii|MYFAB552-11|Germany|Bavaria|||BOLD:AAH4109
Trachelipus rathkii|GOMYR110-17|Germany|Bavaria|Landkreis Eichstaett||BOLD:AAH4109
Trachelipus rathkii|GCRU166-18|Germany||||BOLD:AAH4102
Trachelipus rathkii|BISCE002-17|Germany|North Rhine-Westphalia||Rees|BOLD:AAH4102
Porcellio monticola|GOMYR042-16|Germany|Baden-Wuerttemberg|Landkreis Loerrach||BOLD:ADF5454
Porcellio monticola|GOMYR175-17|Germany|Baden-Wuerttemberg|Landkreis Loerrach||BOLD:ADF5454
Protracheoniscus politus|GCRU400-18|Germany|Thuringia|Bergern bei Reinstaedt||BOLD:AAX8613
Protracheoniscus politus|GCRU183-18|Germany||||BOLD:AAX8613
Protracheoniscus politus|MYFAB551-11|Germany|Bavaria|||BOLD:AAX8613
Protracheoniscus politus|GBMYR274-15|Germany|Bavaria|Landkreis Freyung-Grafenau||BOLD:AAX8613
Protracheoniscus politus|GCRU408-18|Germany|Thuringia|Geisenhain/SHK||BOLD:AAX8613
Protracheoniscus politus|GOMYR264-17|Germany|Bavaria|Landkreis Nuernberger Land||BOLD:AAX8613
Protracheoniscus politus|GOMYR108-17|Germany|Bavaria|Landkreis Regensburg||BOLD:AAX8613
Porcellium conspersum|GCRU389-18|Germany|Thuringia|Wuerchhausen||BOLD:AAV7038
Porcellium conspersum|GCRU206-18|Germany||||BOLD:AAV7038
Porcellium conspersum|GCRU169-18|Germany||||BOLD:AAV7038
Porcellium conspersum|GCRU395-18|Germany|Thuringia|Steinthaleben (Rathsfeld)||BOLD:AAV7038
Porcellium conspersum|GOMYR111-17|Germany|Bavaria|Landkreis Garmisch-Partenkirchen||BOLD:AAV7038
Porcellium conspersum|MYFAB557-11|Switzerland|Aargau|||BOLD:AAV7038
Porcellium conspersum|MYFAB556-11|Germany|Bavaria|||BOLD:AAV7038
Ligia oceanica|BNSIS020-14|North Sea|||Wilhelmshaven|BOLD:AAJ2795
Ligia oceanica|BNSIS016-14|North Sea|||Wilhelmshaven|BOLD:AAJ2795
Ligia oceanica|BNSIS019-14|North Sea|||Wilhelmshaven|BOLD:AAJ2795
Ligia oceanica|BNSIS018-14|North Sea|||Wilhelmshaven|BOLD:AAJ2795
Ligia oceanica|BNSIS017-14|North Sea|||Wilhelmshaven|BOLD:AAJ2795
Ligia oceanica|BISCE068-17|Germany|Lower Saxony||Wilhelmshaven|BOLD:AAJ2795
Ligia oceanica|BISCE067-17|Germany|Lower Saxony||Wilhelmshaven|BOLD:AAJ2795
Ligia oceanica|BISCE066-17|Germany|Lower Saxony||Wilhelmshaven|BOLD:AAJ2795
Ligia oceanica|BISCE065-17|Germany|Lower Saxony||Wilhelmshaven|BOLD:AAJ2795
Ligia oceanica|BISCE064-17|Germany|Lower Saxony||Wilhelmshaven|BOLD:AAJ2795
Ligia oceanica|BISCE063-17|Germany|Lower Saxony||Wilhelmshaven|BOLD:AAJ2795
Tylos ponticus|BISCE029-17|Spain||A Coruna|Ferrol|BOLD:ADK9948
Ligia italica|BISCE021-17|Italy||Grosseto, Giglio||BOLD:ACQ8250
Ligia italica|BISCE020-17|Italy||Grosseto, Giglio||BOLD:ACQ8250

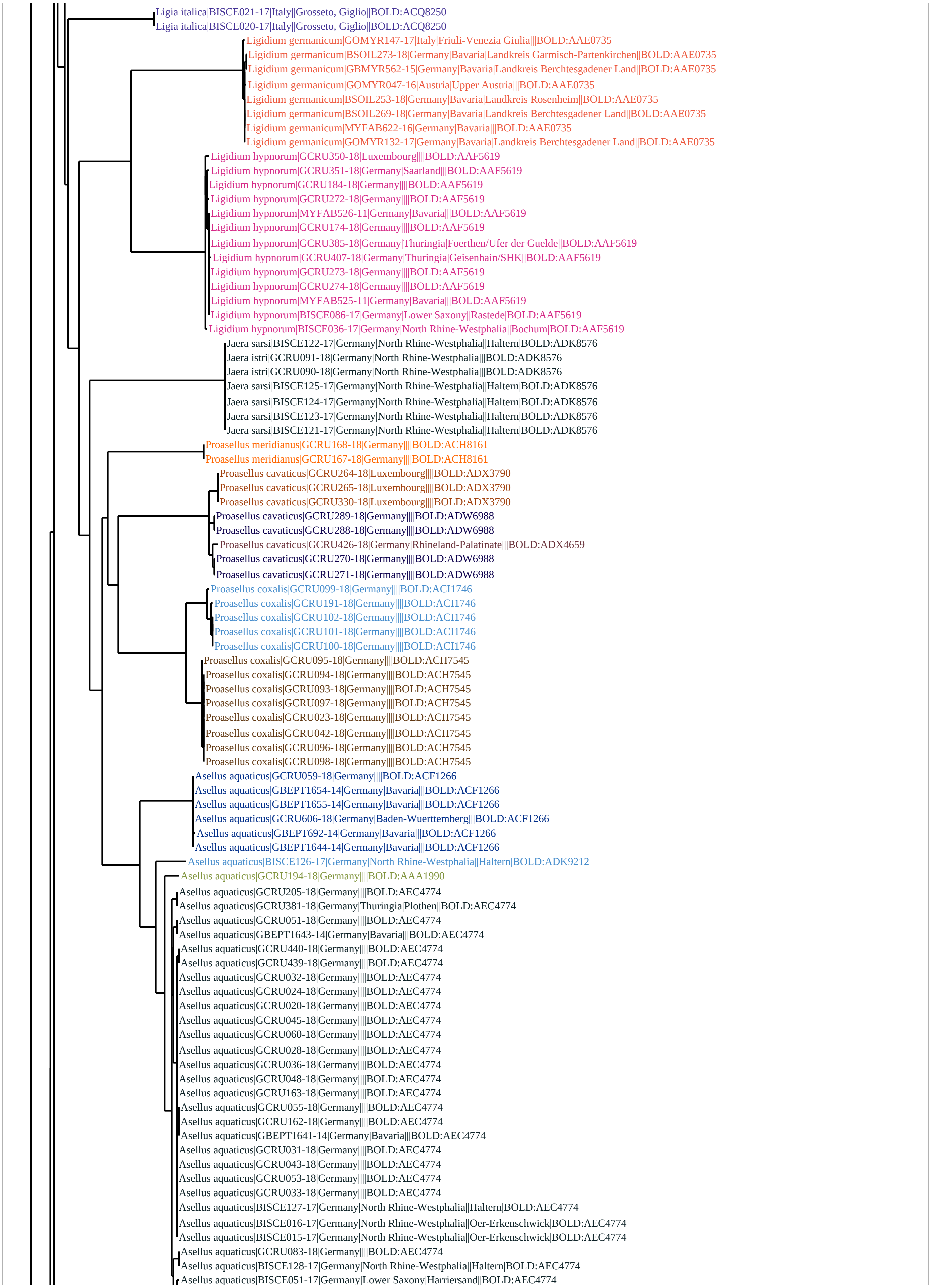
Ligia italica|BISCE021-17|Italy||Grosseto, Giglio||BOLD:ACQ8250
Ligia italica|BISCE020-17|Italy||Grosseto, Giglio||BOLD:ACQ8250
Ligidium germanicum|GOMYR147-17|Italy|Friuli-Venezia Giulia|||BOLD:AAE0735
Ligidium germanicum|BSOIL273-18|Germany|Bavaria|Landkreis Garmisch-Partenkirchen||BOLD:AAE0735
Ligidium germanicum|GBMYR562-15|Germany|Bavaria|Landkreis Berchtesgadener Land||BOLD:AAE0735
Ligidium germanicum|GOMYR047-16|Austria|Upper Austria|||BOLD:AAE0735
Ligidium germanicum|BSOIL253-18|Germany|Bavaria|Landkreis Rosenheim||BOLD:AAE0735
Ligidium germanicum|BSOIL269-18|Germany|Bavaria|Landkreis Berchtesgadener Land||BOLD:AAE0735
Ligidium germanicum|MYFAB622-16|Germany|Bavaria|||BOLD:AAE0735
Ligidium germanicum|GOMYR132-17|Germany|Bavaria|Landkreis Berchtesgadener Land||BOLD:AAE0735
Ligidium hypnorum|GCRU350-18|Luxembourg||||BOLD:AAF5619
Ligidium hypnorum|GCRU351-18|Germany|Saarland|||BOLD:AAF5619
Ligidium hypnorum|GCRU184-18|Germany||||BOLD:AAF5619
Ligidium hypnorum|GCRU272-18|Germany||||BOLD:AAF5619
Ligidium hypnorum|MYFAB526-11|Germany|Bavaria|||BOLD:AAF5619
Ligidium hypnorum|GCRU174-18|Germany||||BOLD:AAF5619
Ligidium hypnorum|GCRU385-18|Germany|Thuringia|Foerthen/Ufer der Guelde||BOLD:AAF5619
Ligidium hypnorum|GCRU407-18|Germany|Thuringia|Geisenhain/SHK||BOLD:AAF5619
Ligidium hypnorum|GCRU273-18|Germany||||BOLD:AAF5619
Ligidium hypnorum|GCRU274-18|Germany||||BOLD:AAF5619
Ligidium hypnorum|MYFAB525-11|Germany|Bavaria|||BOLD:AAF5619
Ligidium hypnorum|BISCE086-17|Germany|Lower Saxony||Rastede|BOLD:AAF5619
Ligidium hypnorum|BISCE036-17|Germany|North Rhine-Westphalia||Bochum|BOLD:AAF5619
Jaera sarsi|BISCE122-17|Germany|North Rhine-Westphalia||Haltern|BOLD:ADK8576
Jaera istri|GCRU091-18|Germany|North Rhine-Westphalia|||BOLD:ADK8576
Jaera istri|GCRU090-18|Germany|North Rhine-Westphalia|||BOLD:ADK8576
Jaera sarsi|BISCE125-17|Germany|North Rhine-Westphalia||Haltern|BOLD:ADK8576
Jaera sarsi|BISCE124-17|Germany|North Rhine-Westphalia||Haltern|BOLD:ADK8576
Jaera sarsi|BISCE123-17|Germany|North Rhine-Westphalia||Haltern|BOLD:ADK8576
Jaera sarsi|BISCE121-17|Germany|North Rhine-Westphalia||Haltern|BOLD:ADK8576
Proasellus meridianus|GCRU168-18|Germany||||BOLD:ACH8161
Proasellus meridianus|GCRU167-18|Germany||||BOLD:ACH8161
Proasellus cavaticus|GCRU264-18|Luxembourg||||BOLD:ADX3790
Proasellus cavaticus|GCRU265-18|Luxembourg||||BOLD:ADX3790
Proasellus cavaticus|GCRU330-18|Luxembourg||||BOLD:ADX3790
Proasellus cavaticus|GCRU289-18|Germany||||BOLD:ADW6988
Proasellus cavaticus|GCRU288-18|Germany||||BOLD:ADW6988
Proasellus cavaticus|GCRU426-18|Germany|Rhineland-Palatinate|||BOLD:ADX4659
Proasellus cavaticus|GCRU270-18|Germany||||BOLD:ADW6988
Proasellus cavaticus|GCRU271-18|Germany||||BOLD:ADW6988
Proasellus coxalis|GCRU099-18|Germany||||BOLD:ACI1746
Proasellus coxalis|GCRU191-18|Germany||||BOLD:ACI1746
Proasellus coxalis|GCRU102-18|Germany||||BOLD:ACI1746
Proasellus coxalis|GCRU101-18|Germany||||BOLD:ACI1746
Proasellus coxalis|GCRU100-18|Germany||||BOLD:ACI1746
Proasellus coxalis|GCRU095-18|Germany||||BOLD:ACH7545
Proasellus coxalis|GCRU094-18|Germany||||BOLD:ACH7545
Proasellus coxalis|GCRU093-18|Germany||||BOLD:ACH7545
Proasellus coxalis|GCRU097-18|Germany||||BOLD:ACH7545
Proasellus coxalis|GCRU023-18|Germany||||BOLD:ACH7545
Proasellus coxalis|GCRU042-18|Germany||||BOLD:ACH7545
Proasellus coxalis|GCRU096-18|Germany||||BOLD:ACH7545
Proasellus coxalis|GCRU098-18|Germany||||BOLD:ACH7545
Asellus aquaticus|GCRU059-18|Germany||||BOLD:ACF1266
Asellus aquaticus|GBEPT1654-14|Germany|Bavaria|||BOLD:ACF1266
Asellus aquaticus|GBEPT1655-14|Germany|Bavaria|||BOLD:ACF1266
Asellus aquaticus|GCRU606-18|Germany|Baden-Wuerttemberg|||BOLD:ACF1266
Asellus aquaticus|GBEPT692-14|Germany|Bavaria|||BOLD:ACF1266
Asellus aquaticus|GBEPT1644-14|Germany|Bavaria|||BOLD:ACF1266
Asellus aquaticus|BISCE126-17|Germany|North Rhine-Westphalia||Haltern|BOLD:ADK9212
Asellus aquaticus|GCRU194-18|Germany||||BOLD:AAA1990
Asellus aquaticus|GCRU205-18|Germany||||BOLD:AEC4774
Asellus aquaticus|GCRU381-18|Germany|Thuringia|Plothen||BOLD:AEC4774
Asellus aquaticus|GCRU051-18|Germany||||BOLD:AEC4774
Asellus aquaticus|GBEPT1643-14|Germany|Bavaria|||BOLD:AEC4774
Asellus aquaticus|GCRU440-18|Germany||||BOLD:AEC4774
Asellus aquaticus|GCRU439-18|Germany||||BOLD:AEC4774
Asellus aquaticus|GCRU032-18|Germany||||BOLD:AEC4774
Asellus aquaticus|GCRU024-18|Germany||||BOLD:AEC4774
Asellus aquaticus|GCRU020-18|Germany||||BOLD:AEC4774
Asellus aquaticus|GCRU045-18|Germany||||BOLD:AEC4774
Asellus aquaticus|GCRU060-18|Germany||||BOLD:AEC4774
Asellus aquaticus|GCRU028-18|Germany||||BOLD:AEC4774
Asellus aquaticus|GCRU036-18|Germany||||BOLD:AEC4774
Asellus aquaticus|GCRU048-18|Germany||||BOLD:AEC4774
Asellus aquaticus|GCRU163-18|Germany||||BOLD:AEC4774
Asellus aquaticus|GCRU055-18|Germany||||BOLD:AEC4774
Asellus aquaticus|GCRU162-18|Germany||||BOLD:AEC4774
Asellus aquaticus|GBEPT1641-14|Germany|Bavaria|||BOLD:AEC4774
Asellus aquaticus|GCRU031-18|Germany||||BOLD:AEC4774
Asellus aquaticus|GCRU043-18|Germany||||BOLD:AEC4774
Asellus aquaticus|GCRU053-18|Germany||||BOLD:AEC4774
Asellus aquaticus|GCRU033-18|Germany||||BOLD:AEC4774
Asellus aquaticus|BISCE127-17|Germany|North Rhine-Westphalia||Haltern|BOLD:AEC4774
Asellus aquaticus|BISCE016-17|Germany|North Rhine-Westphalia||Oer-Erkenschwick|BOLD:AEC4774
Asellus aquaticus|BISCE015-17|Germany|North Rhine-Westphalia||Oer-Erkenschwick|BOLD:AEC4774
Asellus aquaticus|GCRU083-18|Germany||||BOLD:AEC4774
Asellus aquaticus|BISCE128-17|Germany|North Rhine-Westphalia||Haltern|BOLD:AEC4774
Asellus aquaticus|BISCE051-17|Germany|Lower Saxony|Harriersand||BOLD:AEC4774

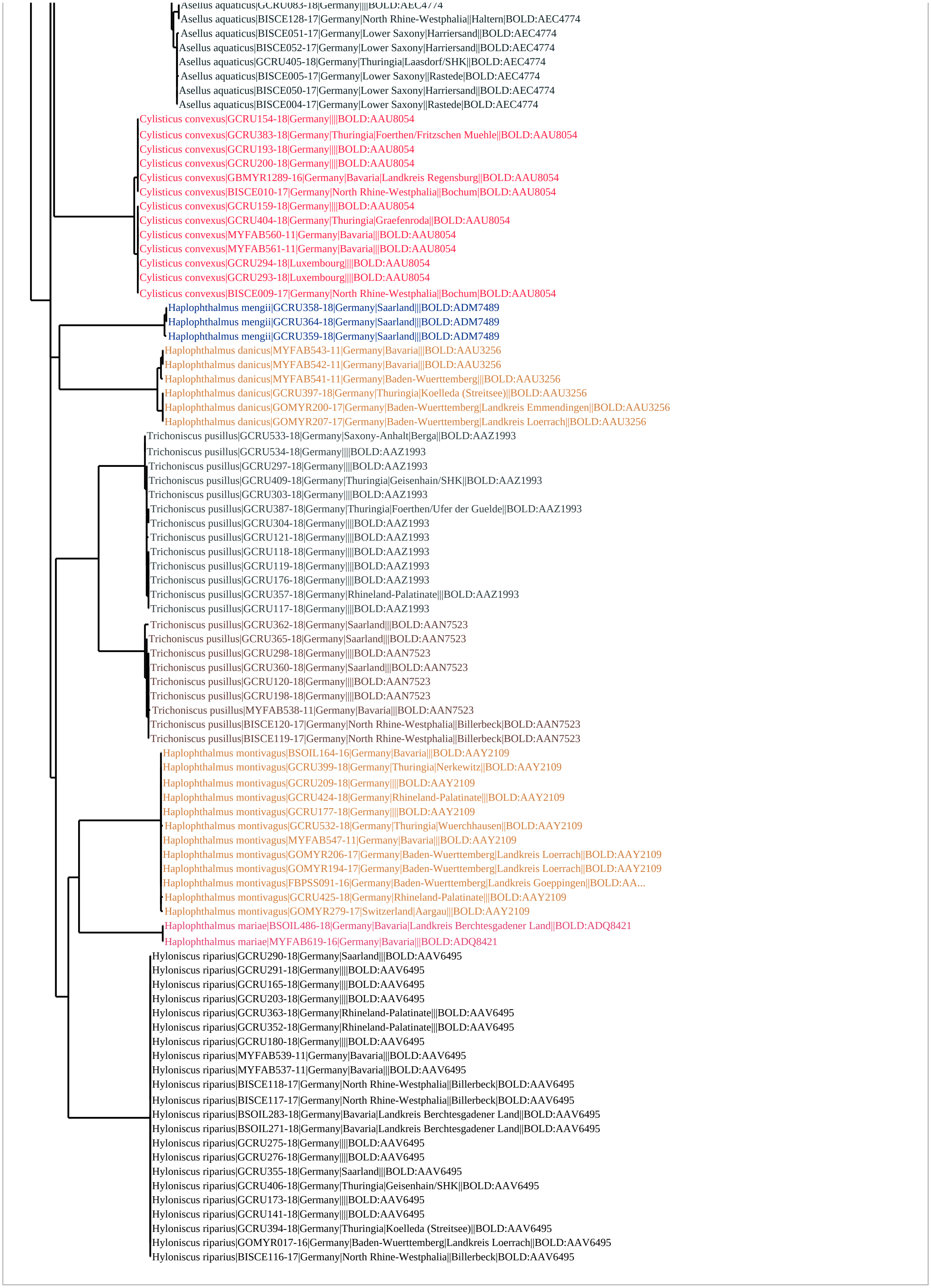
Asellus aquaticus|GCRU083-18|Germany||||BOLD:AEC4774
Asellus aquaticus|BISCE128-17|Germany|North Rhine-Westphalia||Haltern|BOLD:AEC4774
Asellus aquaticus|BISCE051-17|Germany|Lower Saxony|Harriersand||BOLD:AEC4774
Asellus aquaticus|BISCE052-17|Germany|Lower Saxony|Harriersand||BOLD:AEC4774
Asellus aquaticus|GCRU405-18|Germany|Thuringia|Laasdorf/SHK||BOLD:AEC4774
Asellus aquaticus|BISCE005-17|Germany|Lower Saxony||Rastede|BOLD:AEC4774
Asellus aquaticus|BISCE050-17|Germany|Lower Saxony|Harriersand||BOLD:AEC4774
Asellus aquaticus|BISCE004-17|Germany|Lower Saxony||Rastede|BOLD:AEC4774
Cylisticus convexus|GCRU154-18|Germany||||BOLD:AAU8054
Cylisticus convexus|GCRU383-18|Germany|Thuringia|Foerthen/Fritzschen Muehle||BOLD:AAU8054
Cylisticus convexus|GCRU193-18|Germany||||BOLD:AAU8054
Cylisticus convexus|GCRU200-18|Germany||||BOLD:AAU8054
Cylisticus convexus|GBMYR1289-16|Germany|Bavaria|Landkreis Regensburg||BOLD:AAU8054
Cylisticus convexus|BISCE010-17|Germany|North Rhine-Westphalia||Bochum|BOLD:AAU8054
Cylisticus convexus|GCRU159-18|Germany||||BOLD:AAU8054
Cylisticus convexus|GCRU404-18|Germany|Thuringia|Graefenroda||BOLD:AAU8054
Cylisticus convexus|MYFAB560-11|Germany|Bavaria|||BOLD:AAU8054
Cylisticus convexus|MYFAB561-11|Germany|Bavaria|||BOLD:AAU8054
Cylisticus convexus|GCRU294-18|Luxembourg||||BOLD:AAU8054
Cylisticus convexus|GCRU293-18|Luxembourg||||BOLD:AAU8054
Cylisticus convexus|BISCE009-17|Germany|North Rhine-Westphalia||Bochum|BOLD:AAU8054
Haplophthalmus mengii|GCRU358-18|Germany|Saarland|||BOLD:ADM7489
Haplophthalmus mengii|GCRU364-18|Germany|Saarland|||BOLD:ADM7489
Haplophthalmus mengii|GCRU359-18|Germany|Saarland|||BOLD:ADM7489
Haplophthalmus danicus|MYFAB543-11|Germany|Bavaria|||BOLD:AAU3256
Haplophthalmus danicus|MYFAB542-11|Germany|Bavaria|||BOLD:AAU3256
Haplophthalmus danicus|MYFAB541-11|Germany|Baden-Wuerttemberg|||BOLD:AAU3256
Haplophthalmus danicus|GCRU397-18|Germany|Thuringia|Koelleda (Streitsee)||BOLD:AAU3256
Haplophthalmus danicus|GOMYR200-17|Germany|Baden-Wuerttemberg|Landkreis Emmendingen||BOLD:AAU3256
Haplophthalmus danicus|GOMYR207-17|Germany|Baden-Wuerttemberg|Landkreis Loerrach||BOLD:AAU3256
Trichoniscus pusillus|GCRU533-18|Germany|Saxony-Anhalt|Berga||BOLD:AAZ1993
Trichoniscus pusillus|GCRU534-18|Germany||||BOLD:AAZ1993
Trichoniscus pusillus|GCRU297-18|Germany||||BOLD:AAZ1993
Trichoniscus pusillus|GCRU409-18|Germany|Thuringia|Geisenhain/SHK||BOLD:AAZ1993
Trichoniscus pusillus|GCRU303-18|Germany||||BOLD:AAZ1993
Trichoniscus pusillus|GCRU387-18|Germany|Thuringia|Foerthen/Ufer der Guelde||BOLD:AAZ1993
Trichoniscus pusillus|GCRU304-18|Germany||||BOLD:AAZ1993
Trichoniscus pusillus|GCRU121-18|Germany||||BOLD:AAZ1993
Trichoniscus pusillus|GCRU118-18|Germany||||BOLD:AAZ1993
Trichoniscus pusillus|GCRU119-18|Germany||||BOLD:AAZ1993
Trichoniscus pusillus|GCRU176-18|Germany||||BOLD:AAZ1993
Trichoniscus pusillus|GCRU357-18|Germany|Rhineland-Palatinate|||BOLD:AAZ1993
Trichoniscus pusillus|GCRU117-18|Germany||||BOLD:AAZ1993
Trichoniscus pusillus|GCRU362-18|Germany|Saarland|||BOLD:AAN7523
Trichoniscus pusillus|GCRU365-18|Germany|Saarland|||BOLD:AAN7523
Trichoniscus pusillus|GCRU298-18|Germany||||BOLD:AAN7523
Trichoniscus pusillus|GCRU360-18|Germany|Saarland|||BOLD:AAN7523
Trichoniscus pusillus|GCRU120-18|Germany||||BOLD:AAN7523
Trichoniscus pusillus|GCRU198-18|Germany||||BOLD:AAN7523
Trichoniscus pusillus|MYFAB538-11|Germany|Bavaria|||BOLD:AAN7523
Trichoniscus pusillus|BISCE120-17|Germany|North Rhine-Westphalia||Billerbeck|BOLD:AAN7523
Trichoniscus pusillus|BISCE119-17|Germany|North Rhine-Westphalia||Billerbeck|BOLD:AAN7523
Haplophthalmus montivagus|BSOIL164-16|Germany|Bavaria|||BOLD:AAY2109
Haplophthalmus montivagus|GCRU399-18|Germany|Thuringia|Nerkewitz||BOLD:AAY2109
Haplophthalmus montivagus|GCRU209-18|Germany||||BOLD:AAY2109
Haplophthalmus montivagus|GCRU424-18|Germany|Rhineland-Palatinate|||BOLD:AAY2109
Haplophthalmus montivagus|GCRU177-18|Germany||||BOLD:AAY2109
Haplophthalmus montivagus|GCRU532-18|Germany|Thuringia|Wuerchhausen||BOLD:AAY2109
Haplophthalmus montivagus|MYFAB547-11|Germany|Bavaria|||BOLD:AAY2109
Haplophthalmus montivagus|GOMYR206-17|Germany|Baden-Wuerttemberg|Landkreis Loerrach||BOLD:AAY2109
Haplophthalmus montivagus|GOMYR194-17|Germany|Baden-Wuerttemberg|Landkreis Loerrach||BOLD:AAY2109
Haplophthalmus montivagus|FBPSS091-16|Germany|Baden-Wuerttemberg|Landkreis Goeppingen||BOLD:AA...
Haplophthalmus montivagus|GCRU425-18|Germany|Rhineland-Palatinate|||BOLD:AAY2109
Haplophthalmus montivagus|GOMYR279-17|Switzerland|Aargau|||BOLD:AAY2109
Haplophthalmus mariae|BSOIL486-18|Germany|Bavaria|Landkreis Berchtesgadener Land||BOLD:ADQ8421
Haplophthalmus mariae|MYFAB619-16|Germany|Bavaria|||BOLD:ADQ8421
Hyloniscus riparius|GCRU290-18|Germany|Saarland|||BOLD:AAV6495
Hyloniscus riparius|GCRU291-18|Germany||||BOLD:AAV6495
Hyloniscus riparius|GCRU165-18|Germany||||BOLD:AAV6495
Hyloniscus riparius|GCRU203-18|Germany||||BOLD:AAV6495
Hyloniscus riparius|GCRU363-18|Germany|Rhineland-Palatinate|||BOLD:AAV6495
Hyloniscus riparius|GCRU352-18|Germany|Rhineland-Palatinate|||BOLD:AAV6495
Hyloniscus riparius|GCRU180-18|Germany||||BOLD:AAV6495
Hyloniscus riparius|MYFAB539-11|Germany|Bavaria|||BOLD:AAV6495
Hyloniscus riparius|MYFAB537-11|Germany|Bavaria|||BOLD:AAV6495
Hyloniscus riparius|BISCE118-17|Germany|North Rhine-Westphalia||Billerbeck|BOLD:AAV6495
Hyloniscus riparius|BISCE117-17|Germany|North Rhine-Westphalia||Billerbeck|BOLD:AAV6495
Hyloniscus riparius|BSOIL283-18|Germany|Bavaria|Landkreis Berchtesgadener Land||BOLD:AAV6495
Hyloniscus riparius|BSOIL271-18|Germany|Bavaria|Landkreis Berchtesgadener Land||BOLD:AAV6495
Hyloniscus riparius|GCRU275-18|Germany||||BOLD:AAV6495
Hyloniscus riparius|GCRU276-18|Germany||||BOLD:AAV6495
Hyloniscus riparius|GCRU355-18|Germany|Saarland|||BOLD:AAV6495
Hyloniscus riparius|GCRU406-18|Germany|Thuringia|Geisenhain/SHK||BOLD:AAV6495
Hyloniscus riparius|GCRU173-18|Germany||||BOLD:AAV6495
Hyloniscus riparius|GCRU141-18|Germany||||BOLD:AAV6495
Hyloniscus riparius|GCRU394-18|Germany|Thuringia|Koelleda (Streitsee)||BOLD:AAV6495
Hyloniscus riparius|GOMYR017-16|Germany|Baden-Wuerttemberg|Landkreis Loerrach||BOLD:AAV6495
Hyloniscus riparius|BISCE116-17|Germany|North Rhine-Westphalia||Billerbeck|BOLD:AAV6495
